# Hydrazonylthiazole Derivatives as Dual EGFR and ALR2 Inhibitors: Design, Synthesis, and Comprehensive In Vitro and In Silico Evaluation for Potential Anticancer Activity

**DOI:** 10.3390/ph19010050

**Published:** 2025-12-25

**Authors:** Belgin Sever, Cüneyt Türkeş, Yeliz Demir, Khaled M. Elamin, Wadah Osman, Kübra Oral, Selenay Akıncı Genç, Zerrin Cantürk, Takuya Masunaga, Naoki Kishimoto, Shogo Misumi, Masami Otsuka, Mikako Fujita, Halilibrahim Ciftci

**Affiliations:** 1 Department of Pharmaceutical Chemistry, Faculty of Pharmacy, Anadolu University, Eskisehir 26470, Türkiye; belginsever@anadolu.edu.tr (B.S.); kbrorl78@gmail.com (K.O.); selenayakinci@gmail.com (S.A.G.); 2 Medicinal and Biological Chemistry Science Farm Joint Research Laboratory, Faculty of Life Sciences, Kumamoto University, Kumamoto 862-0973, Japan; tmasunaga@kumamoto-u.ac.jp (T.M.); motsuka@gpo.kumamoto-u.ac.jp (M.O.); mfujita@kumamoto-u.ac.jp (M.F.); 3 Department of Biochemistry, Faculty of Pharmacy, Erzincan Binali Yıldırım University, Erzincan 24100, Türkiye; 4 Department of Pharmacy Services, Nihat Delibalta Göle Vocational High School, Ardahan University, Ardahan 75700, Türkiye; yelizdemir@ardahan.edu.tr; 5 Global Center for Natural Products Research, Faculty of Life Sciences, Kumamoto University, 5-1 Oe-honmachi, Chuo-ku, Kumamoto 862-0973, Japan; khaled@kumamoto-u.ac.jp (K.M.E.); naokishi@kumamoto-u.ac.jp (N.K.); misumi@gpo.kumamoto-u.ac.jp (S.M.); 6 Department of Pharmacognosy, Faculty of Pharmacy, Prince Sattam Bin Abdulaziz University, Al-Kharj 11942, Saudi Arabia; w.osman@psau.edu.sa; 7 Department of Pharmaceutical Microbiology, Faculty of Pharmacy, Anadolu University, Eskişehir 26470, Türkiye; zkcanturk@anadolu.edu.tr; 8 Department of Environmental and Molecular Health Sciences, Faculty of Medical and Pharmaceutical Sciences, Kumamoto University, Kumamoto 862-0973, Japan; 9 Department of Drug Discovery, Science Farm Ltd., Kumamoto 862-0976, Japan; 10 Department of Molecular Biology and Genetics, Burdur Mehmet Akif Ersoy University, Istiklal Campus, Burdur 15200, Türkiye

**Keywords:** hydrazonylthiazole, EGFR, ALR2, cytotoxicity, apoptosis, docking, MD

## Abstract

**Background/Objectives**: Signaling imbalances involving epidermal growth factor receptor (EGFR) and aldose reductase (ALR2) are frequently associated with the biology of several solid tumors, including non-small-cell lung cancer (NSCLC) and breast cancer. This work sought to prepare and investigate a small set of hydrazonylthiazole derivatives as potential modulators of both targets with relevance to cancer therapy. **Methods**: Thirteen compounds (**1**–**13**) were synthesized and examined for their effects on A549 (NSCLC), MCF-7 (breast cancer), and Jurkat leukemia cells, together with peripheral blood mononuclear cells (PBMCs) to determine selectivity. The most active molecules were further analyzed through apoptosis studies, EGFR and ALR2 inhibition assays, docking calculations, and 200 ns molecular dynamics (MD) simulations. SwissADME was used to estimate pharmacokinetic and drug-likeness features. **Results**: Among all derivatives, compound **13**, prepared here for the first time, showed the strongest activity on A549 and MCF-7 cells (IC_50_: 1.33 ± 0.41 µM; 1.74 ± 0.38 µM) and displayed a very high selectivity index (SI = 138.9). It also triggered apoptosis in A549 cells and reduced EGFR activity by 74% at 10 µM. In contrast, compound **5** acted as the most efficient ALR2 blocker (*K*_I_ = 0.08 ± 0.01 µM). MD simulations showed that both compounds maintained stable contact patterns with essential residues in the EGFR and ALR2 binding pockets. SwissADME analysis suggested suitable oral absorption and drug-likeness for both molecules. **Conclusions**: Compound **13** behaves as a selective EGFR-directed agent capable of inducing apoptotic cell death in NSCLC, while compound **5** shows strong affinity toward ALR2. These outcomes indicate that both structures may serve as useful starting points for further development of small molecules acting on EGFR- and ALR2-related pathways.

## 1. Introduction

Solid tumors continue to account for most cancer diagnoses worldwide, with epithelial-origin malignancies, most commonly detected in the breast, lung, colon, prostate, and ovary, forming a major portion of the global cancer burden [1]. Data released by the International Agency for Research on Cancer (IARC), a research authority operating under the World Health Organization, indicate that lung cancer currently ranks first in annual incidence with nearly 2.5 million new cases, followed closely by breast cancer with more than 2.3 million newly identified patients each year [2,3,4]. These numbers highlight the persistent challenge posed by these diseases despite decades of therapeutic progress.

Advances in molecular oncology have firmly positioned targeted therapies at the center of modern cancer management. Among the well-studied molecular regulators, the epidermal growth factor receptor (EGFR) plays a central role in the initiation and expansion of tumors, especially in non-small-cell lung cancer (NSCLC). EGFR belongs to the ErbB receptor family, which includes HER-1/EGFR, HER-2, HER-3, and HER-4, and participates in pathways governing cell growth and survival [5,6,7]. Dysregulated EGFR signaling is also an important contributor to breast cancer biology, particularly through its functional relationship with HER-2, which promotes uncontrolled proliferation and therapeutic resistance [8,9].

Several generations of EGFR tyrosine kinase inhibitors (TKIs) have been developed to counter aberrant receptor activity. Reversible first-generation TKIs such as gefitinib, erlotinib, and icotinib block ATP engagement through interactions at the hinge region. Second-generation agents, including afatinib, dacomitinib, vandetanib, and neratinib, form covalent bonds with Cys797 and display more sustained activity. A third wave of covalent inhibitors, osimertinib, avitinib, almonertinib, lazertinib, alflutinib, and rociletinib, was optimized to retain activity against mutation-associated resistance, with osimertinib now widely used as a first-line option for EGFR-mutant NSCLC. More recently, allosteric and non-covalent inhibitors such as EAI045, CH7233163, and BLU-945 have entered development to address resistance that persists despite earlier progress [10,11,12,13,14]. Clinical resistance, inter-patient variability, and limited access to newer TKIs remain substantial obstacles to long-term disease control [15].

EGFR-directed therapy also holds therapeutic relevance beyond lung cancer. Agents designed to suppress HER-2 or the broader ErbB signaling network, such as lapatinib, neratinib, tucatinib, and pyrotinib, have demonstrated clinical benefit in subsets of breast cancer. EGFR overexpression is frequently observed in triple-negative breast cancer (TNBC), where it correlates with aggressive behavior. Nonetheless, clinical responses to EGFR-blocking agents in this subtype have been inconsistent, limiting their broader use [16,17,18].

Aldose reductase (ALR2), a member of the aldo-keto reductase family, catalyzes the first step of the polyol pathway by converting glucose to sorbitol through an NADPH-dependent mechanism. Excessive flux through this route, particularly during chronic hyperglycemia, causes osmotic imbalance and oxidative stress due to disturbed NADPH/NAD^+^ homeostasis, ultimately leading to cellular injury [19,20,21]. Beyond this metabolic function, ALR2 has been implicated in a wide spectrum of diseases, including malignancies of the breast, lung, colon, cervix, and liver, as well as inflammatory and metabolic disorders such as asthma, arthritis, diabetes, atherosclerosis, sepsis, uveitis, and neurodegenerative conditions [22,23,24,25,26]. Recent work by Schwab et al. (2025) demonstrated that blocking the polyol pathway can induce energy failure, DNA damage, and apoptotic death in NSCLC cells [27]. Moreover, the ALR2 inhibitor epalrestat has been reported to restore sensitivity to EGFR TKIs in resistant cell models and delay the emergence of resistance in xenograft systems [28,29].

Our earlier studies involving phthalimide-benzoic acid and chalcone-based ALR2 inhibitors showed that para-halogen substituents and expanded aromatic systems enhance ALR2 affinity through well-defined structure–activity relationships (SARs) [30,31,32,33]. These observations guided the development of hydrazinothiazole frameworks that may affect both EGFR and ALR2 activity. Through biochemical assays, docking studies, and absorption, distribution, metabolism, and excretion (ADME) analyses, we previously found that halogen substitution and extended π-systems play important roles in shaping enzymatic interactions and selectivity [30,31,32,33]. Building on this rationale, the present work integrates these favorable structural motifs into a hydrazonylthiazole core to explore their influence on EGFR-related signaling and ALR2-mediated metabolic stress in cancer.

**Figure 1:**
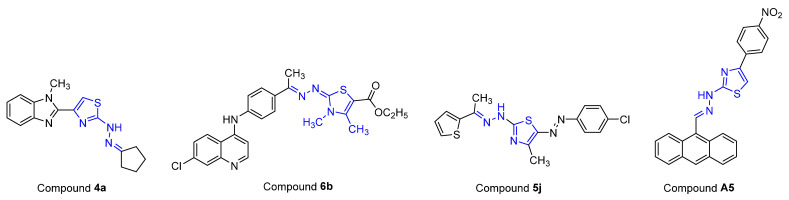
Hydrazonylthiazole derivatives as potential EGFR inhibitors targeting lung and breast cancers: Compounds **4a** [37], **6b** [38], **5j** [40], and **A5** [43].

Thiazole-containing scaffolds are widely used in medicinal chemistry, and several marketed anticancer agents, including dabrafenib, dasatinib, and EAI045, feature thiazole units within their pharmacophores [34,35,36]. Hydrazonylthiazole derivatives have been described in previous reports as EGFR-responsive molecules [36,37,38,39,40,41,42,43] (Figure 1). In addition, benzothiazole analogs such as lidorestat and zopolrestat, along with other thiazole species, have been reported as ALR2 inhibitors [44,45,46].

In this context, a series of hydrazonylthiazole derivatives (**1**–**13**) was rationally designed, synthesized, and evaluated using a panel of biological assays. Guided by previously reported studies and our own earlier findings, the present work focused on the synthesis of para-substituted derivatives. Their cytotoxic effects were assessed in A549 (NSCLC) and MCF-7 (breast cancer) cells, followed by selectivity testing in Jurkat leukemia cells and healthy peripheral blood mononuclear cells (PBMCs). The most active compounds were evaluated for their ability to induce apoptosis and inhibit EGFR. All synthesized molecules were also screened for ALR2 inhibition to assess whether dual modulation of the two pathways could be achieved. To clarify the molecular basis of activity, binding interactions were investigated through molecular docking and extended molecular dynamics (MD) simulations focusing on the ATP-binding region of EGFR and the catalytic cavity of ALR2, with NADPH retained in its native location. Finally, ADME analyses were performed to estimate pharmacokinetic suitability and to support future development of these molecules as multi-target anticancer candidates.

## 2. Results

### 2.1. Chemistry

**Scheme 1:**
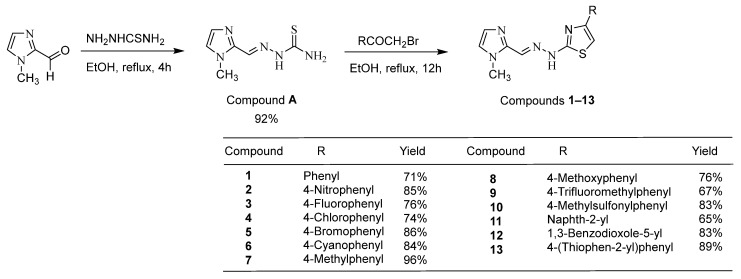
Synthetic pathway for intermediate **A** and its derivatives **1**–**13**, with isolated yields for each compound.

The hydrazonylthiazole derivatives (**1**–**13**) were obtained as outlined in Scheme 1. The synthesis began with the preparation of intermediate **A** [47], formed by condensing 1-methyl-2-imidazolecarboxaldehyde with thiosemicarbazide under refluxing ethanol. This intermediate then reacted with a series of substituted 2-bromo-1-arylethanones, giving the final thiazole products through ring-closure.

Structural verification was carried out using ^1^H NMR, ^13^C NMR and HRMS analyses. All spectroscopic signatures confirmed the successful formation of the expected thiazole scaffolds. While some derivatives (compound **1** [48] and compounds **2**–**8** [49]) matched earlier reports, compounds **9**–**13** were synthesized and structurally defined for the first time in this study. The yields of compounds **1**–**13** were found as 71%, 85%, 76%, 74%, 86%, 84%, 96%, 76%, 67%, 83%, 65%, 83% and 89%, respectively (Scheme 1).

### 2.2. Anticancer Screening and Mechanistic Studies

**Figure 2:**
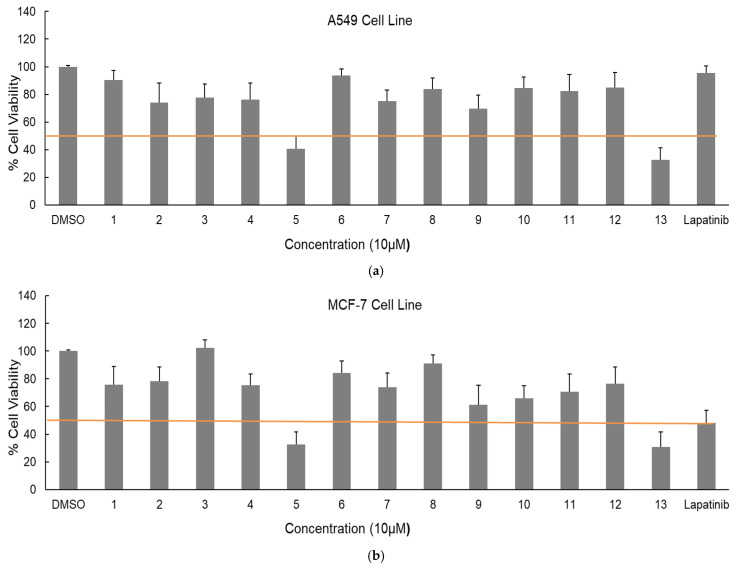
Cytotoxic effects of compounds **1**–**13** in (**a**) A549 and (**b**) MCF-7 cells at 10 µM, compared with lapatinib.

The cytotoxic activity of compounds **1**–**13** was evaluated in A549 (NSCLC) and MCF-7 (breast cancer) cells using the MTT assay, with lapatinib used as a reference. All compounds were tested at 10 µM. Compound **13** produced the greatest reduction in A549 viability (~67%), followed by compound **5** (~59%). Most other derivatives exhibited lower activity (Figure 2a). A similar profile was observed in MCF-7 cells, where compounds **13** and **5** reduced viability by 69% and 67%, respectively (Figure 2b).

Because compounds **5** and **13** reduced viability by more than half at 10 µM, they were selected for IC_50_ determination. Compound **13** showed the strongest activity, with IC_50_ values of 1.33 ± 0.41 µM (A549) and 1.74 ± 0.38 µM (MCF-7) (Table 1). In comparison, lapatinib exhibited considerably higher IC_50_ values (17.95 ± 5.12 µM and 8.43 ± 2.85 µM). Compound **5** also showed meaningful inhibition (A549: 6.87 ± 2.10 µM; MCF-7: 2.78 ± 2.85 µM) (Table 1).

Selectivity evaluation revealed that compound **13** displayed the highest preference for malignant cells, with a SI of 138.90, whereas compound **5** showed a lower but still notable SI value of 37.39 (Table 1). In comparison, lapatinib exhibited markedly weaker selectivity (SI = 7.25), highlighting the superior discrimination of compounds **5** and **13** between healthy PBMCs and Jurkat leukemia cells.

**Figure 3:**
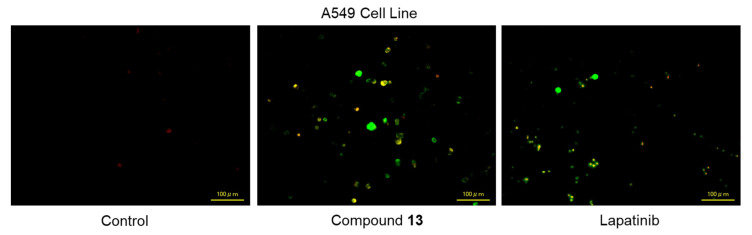
Apoptosis in A549 cells after exposure to compound **13** and lapatinib (24 h).

To explore the mechanism behind its strong cytotoxic effect, compound **13** was examined for apoptosis induction in A549 cells. Annexin V/ethidium homodimer staining showed that compound **13** triggered apoptosis in A549 cells significantly compared with lapatinib (Figure 3). Both treatments produced highly significant increases vs. untreated control.

**Figure 4:**
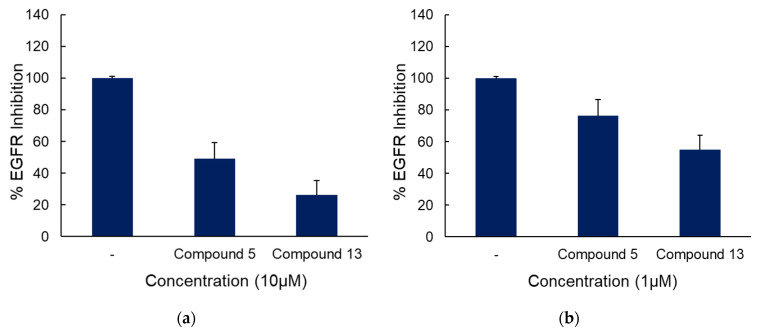
EGFR inhibition by compounds **5** and **13** at (**a**) 10 µM and (**b**) 1 µM.

**Figure 5:**
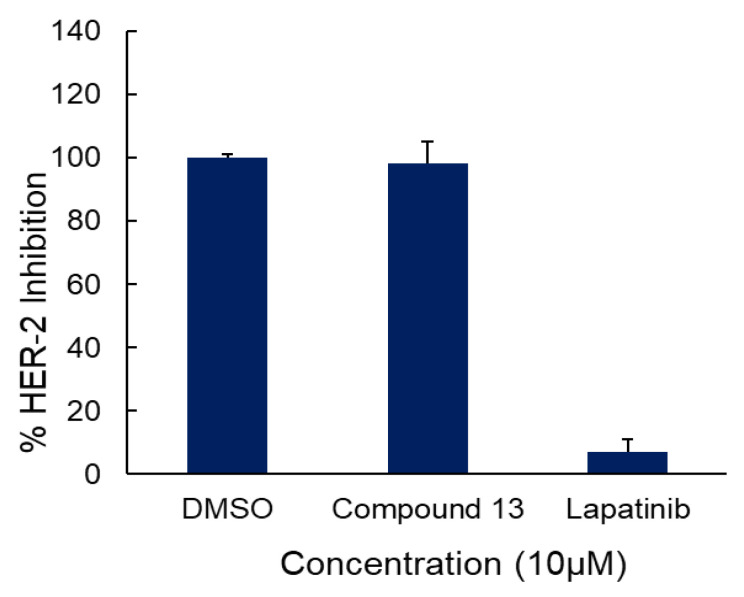
HER-2 inhibition by compound **13** at 10 µM.

Compound **13** strongly reduced EGFR enzymatic activity (74% at 10 µM; 45% at 1 µM). Compound **5** produced moderate inhibition (51% and 23%) (Figure 4a,b). Compound **13** exhibited 1.89% HER-2 inhibition, whereas lapatinib showed 92.96% inhibition (Figure 5), demonstrating a clear selectivity toward EGFR.

To characterize the ALR2 inhibitory potential of the hydrazinothiazole series, inhibition constants (*K*_I_) were determined for all derivatives **1**–**13** using ALR2 and DL-glyceraldehyde as the substrate (Table 2). Under standardized assay conditions, the *K*_I_ values spanned a low micromolar window, ranging from 0.08 to 2.64 µM. This indicates that the scaffold is capable of engaging productively with the ALR2 active site. The clinical reference inhibitor epalrestat (EPR) displayed a *K*_I_ of 0.86 ± 0.06 µM, thereby providing an internal benchmark for comparing the newly synthesized compounds.

Within the series, compound **5**, bearing a para-bromophenyl substituent, emerged as the most potent inhibitor (*K*_I_ = 0.08 ± 0.01 µM), displaying approximately an order-of-magnitude stronger inhibition than the reference drug EPR (*K*_I_ = 0.86 ± 0.06 µM). Submicromolar inhibitory activity was also observed for compounds **4** (4-chlorophenyl), **6** (4-cyanophenyl), **7** (4-methylphenyl), **8** (4-methoxyphenyl), and **10** (4-(methylsulfonyl)phenyl), with *K*_I_ values between 0.21 and 0.28 µM, indicating that the para position of the terminal phenyl ring accommodates both moderately electron-withdrawing and electron-donating substituents without compromising binding affinity. In contrast, the unsubstituted phenyl derivative **1**, the strongly electron-withdrawing 4-nitrophenyl analog **2**, and the more polar or sterically demanding derivatives **11**–**13** exhibited only modest inhibitory activity (*K*_I_ ≥ 1.83 µM), suggesting that excessive polarity or bulk at the distal aromatic ring may hinder optimal accommodation within the ALR2 binding pocket (Table 2). Overall, the SAR analysis highlights the importance of hydrophobic and halogen-associated interactions at the para-substituted phenyl ring, with the superior performance of compound **5** being consistent with stabilizing interactions within the ALR2 catalytic tunnel, as further supported by molecular docking and MD simulations.

### 2.3. In Silico Studies

**Figure 6:**
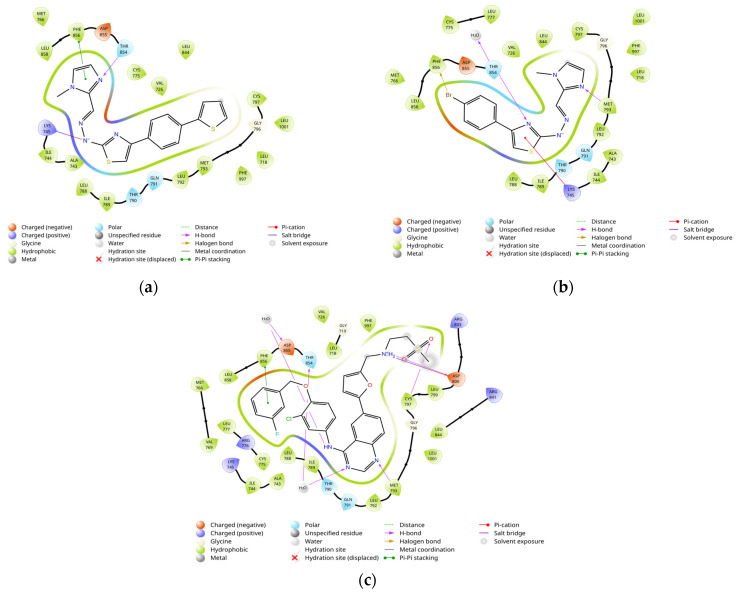
The 2D IFD interaction diagrams of compounds (**a**) **13**, (**b**) **5** and (**c**) lapatinib bound to EGFR.

**Figure 7:**
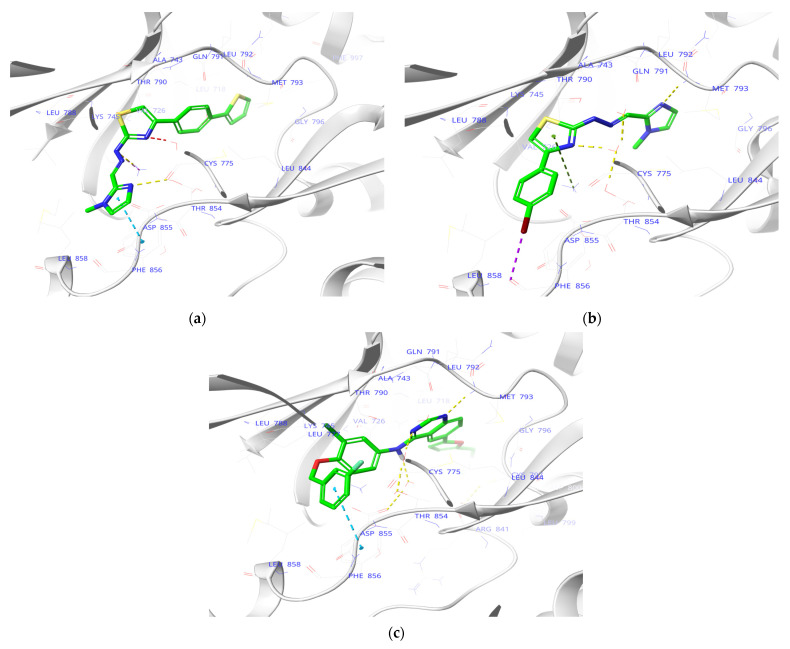
The 3D IFD interaction diagrams of compounds (**a**) **13**, (**b**) **5** and (**c**) lapatinib bound to EGFR.

To characterize the binding behavior of the most active derivatives, induced fit docking (IFD) calculations were performed for compounds **13** and **5** within the ATP-binding region of EGFR (PDB ID: 3POZ) [50] using the Maestro workflow [51]. In these simulations, compound **13** adopted a stable pose in the binding cavity and engaged in several key interactions, including a hydrogen bond with Thr854, a salt-bridge interaction with Lys745, and π-π stacking with Phe856 (Figure 6a and Figure 7a). Compound **5** showed a related pattern of contacts, forming hydrogen bonds with Met793 and Thr854, a π-cation interaction with Lys745, and a halogen-associated contact involving Phe856 (Figure 6b and Figure 7b). For context, lapatinib bound through a more extensive interaction network, involving hydrogen bonds with Asp855, Cys797, Met793, and Thr854, a π-cation interaction with Lys745, and π-π stacking with Phe856. The reference inhibitor also formed additional hydrogen-bond and salt-bridge contacts with Asp800 (Figure 6c and Figure 7c). Taken together, these observations indicate that although compounds **13** and **5** engage fewer residues than lapatinib, they still adopt well-positioned orientations supported by consistent stabilizing interactions at the ATP-binding pocket.

Docking scores and MM-GBSA calculations offered a clearer view of how the compounds behaved inside the EGFR pocket (Table 3). As anticipated, lapatinib produced the most negative MM-GBSA value (−90.38 kcal/mol) and the lowest docking score (−13.54 kcal/mol), reflecting its established inhibitory strength. Compounds **13** and **5** displayed MM-GBSA energies of −41.75 and −44.11 kcal/mol, respectively, together with docking scores of −10.92 and −10.68 kcal/mol. Although these energies were less negative than those of lapatinib, the docking scores show that both molecules adopt well-fitted poses and maintain coherent interaction patterns within the EGFR cavity. The IFD calculations were consistent with these results. The IFD energies obtained for compound **13** (−628.31 kcal/mol) and compound **5** (−627.47 kcal/mol) were close to the value calculated for lapatinib (−638.87 kcal/mol). These energetic trends indicate that compounds **13** and **5** adapt smoothly to local structural rearrangements within the binding site and retain stable orientations, supporting their suitability for EGFR-directed activity.

The Table S1 also reports the energetic details underlying Δ*G*_bind_ with the full Prime MM-GBSA energetic decomposition, including the Coulombic, covalent, hydrogen-bonding, lipophilic, packing, self-contact, solvation (GB), and van der Waals (vdW) contributions for the co-crystallized ligand, compounds **5** and **13**. As implemented in Prime MM-GBSA, Δ*G*_bind_ is obtained as the exact numerical summation of these physically meaningful interaction energy components, which together satisfy the thermodynamic relationship:

$$\Delta G_{\mathrm{bind}}=E_{\mathrm{complex}}-\left(E_{\mathrm{protein}}+E_{\mathrm{ligand}}\right)$$

Accordingly, although Prime does not explicitly output absolute values of *E*_complex_, *E*_protein_, and *E*_ligand_ separately, the reported Δ*G*_bind_ values are directly reproduced by the summation of the listed energetic components, ensuring full computational transparency and reproducibility.

**Figure 8:**
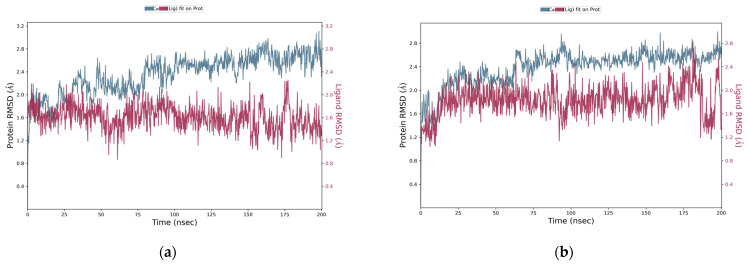
RMSD trajectories of EGFR complexes with compounds (**a**) **13** and (**b**) **5** over 200 ns.

MD simulations were carried out to examine how each ligand behaved within EGFR over time. The root mean square deviation (RMSD) plots demonstrated that the protein-compound **13** complex stabilized shortly after the simulation began, with backbone deviations narrowing to 2.4–2.8 Å. The ligand displayed RMSD values of 1.2–1.8 Å, indicating that its orientation in the ATP pocket remained steady throughout the run and showed no evidence of drifting away from the binding region (Figure 8a). The simulation profile for the compound **5** complex followed the same pattern. EGFR reached a stable state with backbone RMSD values of 2.2–2.6 Å, while the ligand RMSD fluctuated between 1.2 and 2.0 Å. These brief shifts reflected routine positional adjustments rather than loss of binding. Overall, the data show that both ligands maintained well-defined and persistent binding geometries during the full simulation period (Figure 8b).

**Figure 9:**
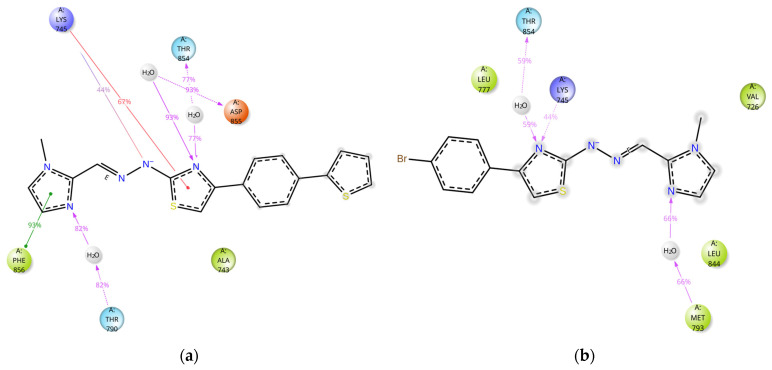
Interaction fractions between ligand atoms of compounds (**a**) **13** and (**b**) **5** and EGFR residues.

Residue-level analyses across the 200 ns trajectory showed that each ligand formed long-lasting contacts with specific amino acids in the EGFR pocket. For compound **13**, the most persistent interactions were observed with Phe856 and Asp855, each maintained for roughly 93% of the simulation. Additional contacts were noted with Thr790 (82%), Thr854 (77%), and Lys745 (two interaction modes recorded at 67% and 44%) (Figure 9a). In comparison, compound **5** consistently engaged Met793 (66%), Thr854 (59%), and Lys745 (44%), demonstrating that the ligand remained firmly positioned within the binding cavity throughout the simulation (Figure 9b).

**Figure 10:**
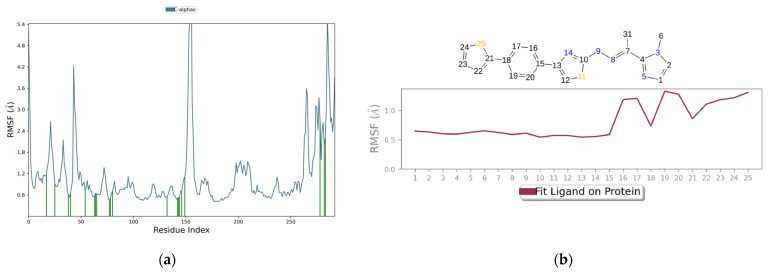
(**a**) Protein RMSF and (**b**) Ligand RMSF plots of compound **13** with EGFR during 200 ns MD simulation.

Root mean square fluctuation (RMSF) analysis offered additional information about the MD behavior of the EGFR-compound **13** complex. Most residues displayed fluctuations below 1.5 Å, reflecting a stable backbone conformation throughout the simulation (Figure 10a). Slightly elevated motion was observed around residues 40, 150, and 280–300, which correspond to loop segments or solvent-accessible regions. The ligand atoms also showed low mobility, with RMSF values generally remaining under 1.0 Å (Figure 10b), indicating limited internal flexibility and a firmly maintained position inside the binding cavity.

**Figure 11:**
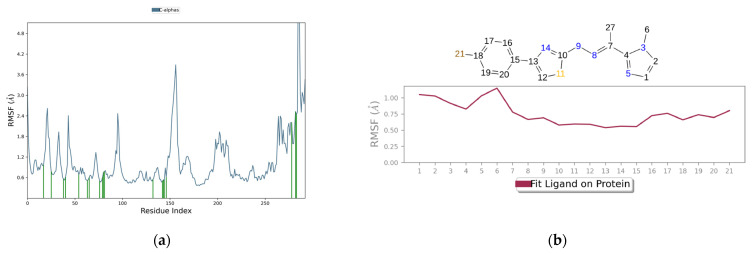
(**a**) Protein RMSF and (**b**) Ligand RMSF plots of compound **5** with EGFR during 200 ns MD simulation.

In the complex formed with compound **5**, the EGFR backbone also exhibited limited movement, with most residues fluctuating below 1.5 Å apart from several flexible loop regions (Figure 11a). The ligand showed similarly restrained motion, with RMSF values generally remaining under 1.0 Å, and only the outermost substituents displaying slightly increased mobility (Figure 11b).

**Figure 12:**
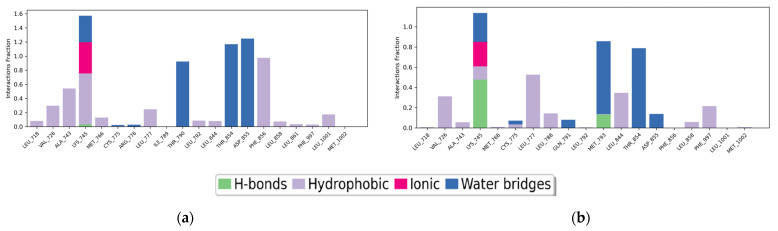
Protein-ligand interaction profiles of compounds (**a**) **13** and (**b**) **5** within the ATP-binding site of EGFR.

Analysis of the interaction-fraction diagrams demonstrated clear differences in how the two ligands occupied the EGFR pocket. Compound **13** (Figure 12a) contacted a wide array of residues and formed a mixture of hydrophobic, hydrogen-bonding, ionic, and water-assisted interactions. The most persistent contacts involved Lys745, Ala743, Val726, Thr790, Asp855, and Phe856, indicating a well-distributed interaction network that aligns with its strong inhibitory effect. In contrast, compound **5** (Figure 12b) engaged in a more limited but still robust set of interactions, primarily involving Lys745, Met793, Thr854, and several hydrophobic residues such as Val726 and Leu844, reflecting a tighter but less extensive binding footprint.

**Figure 13:**
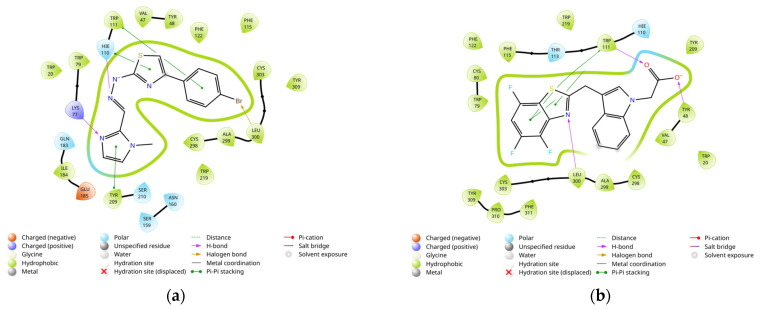
The 2D IFD interaction diagrams of (**a**) compound **5** and (**b**) lidorestat with ALR2.

**Figure 14:**
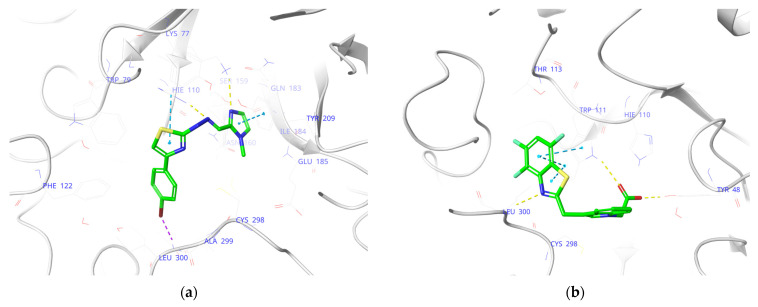
The 3D IFD interaction diagrams of (**a**) compound **5** and (**b**) lidorestat with ALR2.

Given the strong ALR2 inhibition observed experimentally, compound **5** was explored in more detail within the ALR2 catalytic region (PDB ID: 1Z3N) [52] while keeping NADPH in its cofactor position. The docking analysis revealed several key contacts that stabilized the complex, most notably with Leu300, Trp111, His110, Lys77, and Tyr209. These interactions were dominated by hydrogen bonds and π-π stacking (Figure 13a and Figure 14a). The overall interaction pattern resembled that of lidorestat (Figure 13b and Figure 14b), which served as the reference ligand, lending additional support to the predicted binding orientation.

From an energetic standpoint, compound **5** generated a docking score of −10.25 kcal/mol and an MM-GBSA energy of −35.76 kcal/mol, both consistent with a strong binding tendency (Table 3 and Table S1). Its IFD energy (−691.12 kcal/mol) became even more favorable once receptor flexibility was incorporated, indicating enhanced stabilization after local structural adjustment (Table 3).

**Figure 15:**
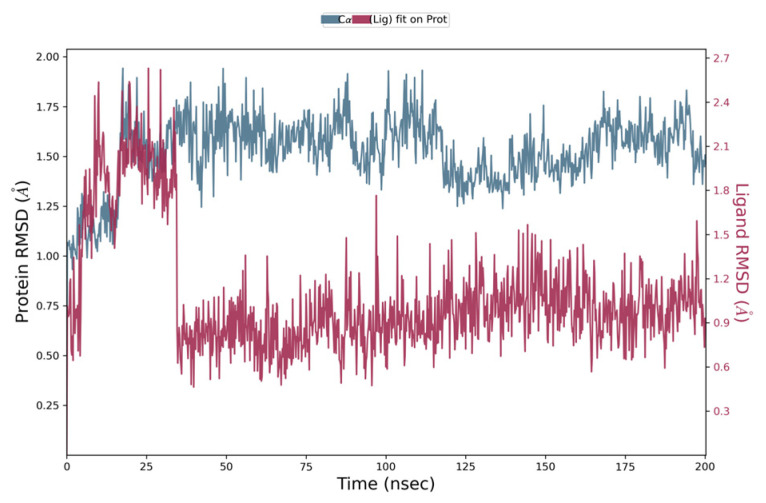
RMSD plot for compound **5** in complex with ALR2 over a 200 ns simulation period.

The MD run for the ALR2-compound **5** complex indicated that the protein backbone fluctuated only within 1.5–1.8 Å, while the ligand remained within 0.5–1.2 Å. These values show that the ligand held a secure position in the active site for the entire 200 ns simulation (Figure 15).

**Figure 16:**
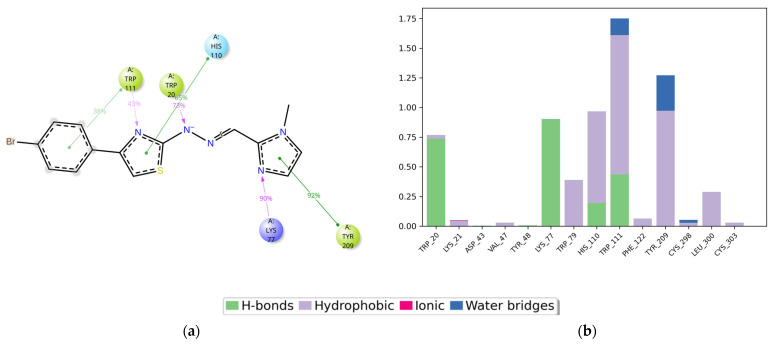
Schematic representation of the detailed interactions between the ligand atoms of compound **5** and the corresponding protein residues (**a**); protein-ligand interaction profile of compound **5** at the active site of ALR2 in the presence of bound NADPH (**b**).

Residue-based interaction profiling showed that several amino acids remained engaged with compound **5** for most of the trajectory. The most frequent contacts were observed with Tyr209 (93%), Lys77 (90%), Trp20 (73%), His110 (65%), and Trp111 (43%/39%) (Figure 16a,b). Among these, Tyr209 and Trp111 are well recognized in the ALR2 literature as key positions that help stabilize high-affinity inhibitors within the catalytic pocket.

**Figure 17:**
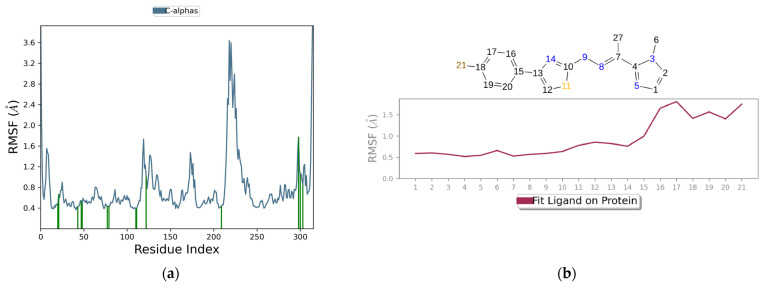
Protein RMSF (**a**) and Ligand RMSF (**b**) plots of compound **5** with ALR2 during 200 ns MD simulation.

RMSF evaluation showed that the ALR2 binding pocket remained largely rigid during its interaction with compound **5** (Figure 17a,b). Most amino acids fluctuated only slightly, whereas regions around residues −110 and −210 displayed the higher mobility expected for flexible loops. The ligand exhibited RMSF values below 1.0 Å for nearly all heavy atoms, with only solvent-exposed substituents showing modest additional motion.

Key pharmacokinetic and drug-likeness properties of compounds **5** and **13** were assessed with SwissADME [53]. The predictions indicated that both molecules fall within Lipinski’s guidelines, exhibit high estimated gastrointestinal (GI) absorption, display moderate lipophilicity, and are not expected to act as P-gp substrates. Compound **13** showed comparatively reduced aqueous solubility, consistent with its more extended aromatic structure. Both molecules were predicted to inhibit several CYP450 enzymes (CYP1A2, CYP2C19, CYP2C9, CYP3A4), whereas neither was predicted to block CYP2D6. No PAINS-related alerts were detected, and the synthetic accessibility values were within acceptable ranges, showing only minor deviations from standard lead-likeness profiles (Table 4).

## 3. Discussion

Advances in targeted anticancer therapy have led to the approval of numerous kinase-directed agents; however, clinical outcomes remain limited by resistance, heterogeneous tumor biology, and incomplete inhibition of compensatory signaling networks [54]. This ongoing gap highlights the demand for chemical scaffolds capable of influencing multiple pathways that shape malignant behavior. EGFR and ALR2 represent two such regulatory nodes, as both contribute to proliferative and stress-associated processes relevant to NSCLC and breast malignancies. The hydrazonylthiazole library developed in this work was created with this therapeutic rationale in mind.

Although a subset of molecules within the series has appeared in previous studies for unrelated purposes such as the Zn(II) complex of compound **1** described for photo-oxidative behavior [48], and compounds **2**–**8** reported as cholinesterase inhibitors [49], the remaining analogs (**9**–**13**) are newly disclosed. Importantly, none of the compounds had been assessed for anticancer activity prior to this work, making this study the first systematic examination of this series in cancer-relevant studies. The results therefore expand the biological scope of these derivatives beyond their earlier functional assignments.

Cytotoxicity screening identified two molecules, compounds **5** and **13**, as the most active across A549 and MCF-7 cells, while preserving minimal effects on PBMCs. This selective behavior suggests that the scaffold interacts preferentially with signaling features enriched in malignant cells. Compound **13** showed a pronounced effect in NSCLC cells by increasing apoptotic signals and inhibiting EGFR significantly, with no meaningful suppression of HER-2. Structural analyses supported this profile: docking and IFD revealed compact alignment within the EGFR ATP pocket, stabilized by persistent interactions with Thr790, Thr854, Asp855, Phe856, and Lys745. Extended MD simulations confirmed that its heterocyclic core remained tightly positioned inside the catalytic region with low conformational drift.

In the literature, hydrazinyl thiazole derivatives have been extensively investigated for their EGFR inhibition-associated anticancer activity against lung and breast cancer cells. Srour et al. (2020) [37] reported the synthesis of thiazole-, benzimidazole-, and hydrazine-based derivatives and evaluated their EGFR inhibitory activity along with antiproliferative effects against MCF-7 cancer cells. Among these compounds, the cyclopentyl-substituted analog **4a** (Figure 1) exhibited pronounced anticancer activity against MCF-7 cells (IC_50_ = 6.30 µM) together with strong EGFR inhibition (IC_50_ = 109.71 nM). In a related study, quinoline-thiazole hydrazone derivatives were designed, synthesized, and evaluated for their in vitro anticancer activity against the MCF-7 cell line. Among them, compound **6b** (Figure 1) demonstrated the most potent antiproliferative activity (IC_50_ = 5.35 µM) and exhibited notable EGFR inhibition with an IC_50_ value of 9.34 ng/mL [38].

El-Naggar et al. (2022) [40] synthesized a series of hydrazinyl thiazole derivatives and assessed their in vitro cytotoxic activity against various cancer cell lines, including MCF-7. Among these, compound **5j** (Figure 1) showed significant cytotoxicity toward MCF-7 cells with an IC_50_ value of 10.87 µM and inhibited EGFR with an IC_50_ of 82.8 nM. In another study, hydrazinyl anthracene-based thiazoles were synthesized and evaluated against both A549 and MCF-7 cell lines. Compound **A5** (Figure 1) demonstrated potent cytotoxic effects against MCF-7 and A549 cells, with IC_50_ values of 7.00 µM and 8.51 µM, respectively, and showed EGFR inhibition with an IC_50_ value of 0.88 µM [43].

When compared with these literature reports on hydrazinyl thiazole derivatives exhibiting EGFR inhibitory activity and anti-breast or anti-lung cancer effects, most compounds were primarily evaluated against MCF-7 cells and generally displayed weaker antiproliferative activity than compound **13** reported in the present study. However, several of these derivatives exhibited stronger EGFR inhibitory potency. This discrepancy may be attributed not only to the common hydrazinyl thiazole core but also to substantial structural differences in the peripheral substituents, including variations in aromatic systems, lipophilicity, and steric bulk, which can markedly influence both EGFR binding affinity and cellular antiproliferative responses.

Compound **5** displayed a different mechanistic pattern. It showed moderate activity toward EGFR yet produced strong ALR2 inhibition, in line with structural trends highlighting the influence of para-substituted aryl residues on ALR2 recognition. The 4-bromophenyl moiety in compound **5** aligned well with the hydrophobic architecture of the ALR2 active region, enabling stable interactions with Trp111, Tyr209, Lys77, His110, and Trp20, all of which maintained high contact frequencies during MD simulations. This stable binding mode mirrors the interaction network observed for the benchmark inhibitor lidorestat, further validating ALR2 as a relevant target for this scaffold.

Structure-activity tendencies across the library indicate that small electronic or steric adjustments at the distal aryl position can shift the balance toward either EGFR-focused or ALR2-focused behavior, illustrating that the hydrazonylthiazole core is highly tunable. Computational predictions via SwissADME revealed suitable drug-likeness for both lead compounds, including good oral bioavailability scores, moderate lipophilicity, and high predicted intestinal passage without P-gp-mediated efflux. Solubility differences reflected variations in aromatic surface area, and neither compound raised PAINS alerts.

Taken together, these findings show that hydrazonylthiazole scaffolds can be adapted to influence two clinically significant cancer-related pathways. Compound **13** operates as a potent EGFR-directed agent with clear apoptotic effects in NSCLC cells, while compound **5** functions as a high-affinity ALR2 inhibitor with preserved antiproliferative potential. Concordance between experimental assays and computational models reinforces the value of this chemical architecture as a platform for multitarget anticancer design.

## 4. Materials and Methods

### 4.1. Chemistry

All reagents and solvents were purchased from standard commercial suppliers and were used as received unless otherwise specified. Melting points were measured on a Mettler Toledo MP90 digital apparatus (Mettler-Toledo, LLC, Columbus, OH, USA) and are provided without correction. Reaction progress and compound purity were monitored by thin-layer chromatography (TLC) using Merck silica gel 60 F254 plates (Darmstadt, Germany). NMR spectra (^1^H and ^13^C) were obtained on a Bruker NMR spectrometer (Bruker, Billerica, MA, USA), and HRMS data were recorded using a Shimadzu LCMS-IT-TOF instrument (Shimadzu, Kyoto, Japan).

#### 4.1.1. Synthesis of Compound **A**

A mixture of 1-methyl-2-imidazolecarboxaldehyde (0.025 mol) and thiosemicarbazide (0.025 mol) was dissolved in ethanol (40 mL) and heated under reflux for 12 h. Upon completion of the reaction, the mixture was cooled to room temperature, leading to the formation of a solid precipitate. The resulting material was collected by filtration, washed, and dried under reduced pressure. Final purification was achieved through recrystallization from ethanol, affording intermediate **A** in analytically pure form, consistent with previously published procedures [45].

(E)-2-((1-Methyl-1*H*-imidazol-2-yl)methylene)hydrazine-1-carbothioamide (**A**) [47]: Yield: 92%. M.p. 228–230 °C. ^1^H NMR (500 MHz, DMSO-*d*_6_) *δ* (ppm): 3.89 (s, 3H), 7.03 (s, 1H), 7.31 (s, 1H), 7.52 (s, 1H), 8.11 (s, 1H), 8.33 (s, 1H), 11.48 (s, 1H). ^13^C NMR (125 MHz, DMSO-*d*_6_) *δ* (ppm): 35.9 (CH_3_), 125.6 (CH), 129.0 (CH), 135.9 (C), 141.0 (CH), 177.9 (C=S). (Spectral Data: Supplementary Information. Figures S1 and S2).

#### 4.1.2. Synthesis of Compounds **1**–**13**

Compound **A** (0.001 mol) was combined with the appropriate 2-bromo-1-arylethanone derivative (0.001 mol) in ethanol (20 mL), and the reaction mixture was heated under reflux for 6 h. After the mixture was allowed to cool to room temperature, a solid product formed, which was isolated by filtration. The crude material was subsequently purified by recrystallization from ethanol, yielding the corresponding thiazole derivative in analytically pure form, consistent with previously described procedures [45].

1-[(4-Phenyl-1,3-thiazol-2-yl)amino]-2-[(1-methyl-1*H*-imidazol-2-yl)methylidene]hydrazine (**1**) [48]: Yield: 71%. For crystallization: 0.329 g compound, 10 mL ethanol. M.p. 223–224 °C. ^1^H NMR (500 MHz, DMSO-*d*_6_) *δ* (ppm): 4.01 (s, 3H), 7.33 (t, *J* = 7.5 Hz, 1H), 7.44 (t, *J* = 7.7 Hz, 2H), 7.52 (s, 1H), 7.74 (s, 1H), 7.82 (s, 1H), 7.88 (d, *J* = 7.4 Hz, 2H), 8.18 (s, 1H), 13.11 (s, 1H). ^13^C NMR (125 MHz, DMSO-*d*_6_) *δ* (ppm): 36.0 (CH_3_), 105.8 (CH), 119.9 (CH), 125.4 (CH), 125.7 (3CH), 127.8 (C), 128.9 (2CH), 134.1 (C), 139.2 (CH), 150.8 (C), 167.4 (C). HRMS (ESI) calcd. for C_14_H_13_N_5_S [M+H]^+^: (*m*/*z*) = 284.0964; found: 284.0966. (Spectral Data: Supplementary Information. Figures S3–S6).

1-[(4-(4-Nitrophenyl)-1,3-thiazol-2-yl)amino]-2-[(1-methyl-1*H*-imidazol-2-yl)methylidene]hydrazine (**2**) [49]: Yield: 85%. For crystallization: 0.458 g compound, 15 mL ethanol. M.p. 256–257 °C. ^1^H NMR (500 MHz, DMSO-*d*_6_) *δ* (ppm): 3.99 (s, 3H), 7.74–7.77 (m, 1H), 7.82 (s, 1H), 7.88 (s, 1H), 8.09 (t, *J* = 8.8 Hz, 2H), 8.17 (s, 1H), 8.26 (d, *J* = 9.2 Hz, 2H), 13.21 (s, 1H). ^13^C NMR (125 MHz, DMSO-*d*_6_) *δ* (ppm): 36.5 (CH_3_), 110.5 (CH), 119.9 (CH), 124.1 (2CH), 124.8 (CH), 125.5 (C), 126.4 (2CH), 138.8 (C), 140.3 (CH, C), 146.6 (C), 167.4 (C). HRMS (ESI) calcd. for C_14_H_12_N_6_O_2_S [M+H]^+^: (*m*/*z*) = 329.0815; found: 329.0815. (Spectral Data: Supplementary Information. Figures S7–S10).

1-[(4-(4-Fluorophenyl)-1,3-thiazol-2-yl)amino]-2-[(1-methyl-1*H*-imidazol-2-yl)methylidene]hydrazine (**3**) [49]: Yield: 76%. For crystallization: 0.375 g compound, 12 mL ethanol. M.p. 245–246 °C. ^1^H NMR (500 MHz, DMSO-*d*_6_) *δ* (ppm): 4.03 (s, 3H), 7.27 (t, *J* = 8.9 Hz, 2H), 7.5 (s, 1H), 7.74–7.93 (m, 4H), 8.15 (s, 1H), 13.13 (s, 1H). ^13^C NMR (125 MHz, DMSO-*d*_6_) *δ* (ppm): 36.3 (CH_3_), 105.4 (CH), 115.6 (d, *J* = 86.5 Hz, 2CH), 119.7 (CH), 125.6 (CH), 127.6 (d, *J* = 35.8 Hz, 2CH), 129.9 (C), 139.3 (CH, C), 157.8 (2C), 162.7 (C). HRMS (ESI) calcd. for C_14_H_12_FN_5_S [M+H]^+^: (*m*/*z*) = 320.0870; found: 320.0880 (Spectral Data: Supplementary Information. Figures S11–S14).

1-[(4-(4-Chlorophenyl)-1,3-thiazol-2-yl)amino]-2-[(1-methyl-1*H*-imidazol-2-yl)methylidene]hydrazine (**4**) [49]: Yield: 74%. For crystallization: 0.386 g compound, 14 mL ethanol. M.p. 253–254 °C. ^1^H NMR (500 MHz, DMSO-*d*_6_) *δ* (ppm): 4.01 (s, 3H), 7.49 (d, *J* = 8.4 Hz, 2H), 7.59 (s, 1H), 7.75 (s, 1H), 7.82 (s, 1H), 7.89 (d, *J* = 8.6 Hz, 2H), 8.15 (s, 1H), 13.15 (s, 1H). ^13^C NMR (125 MHz, DMSO-*d*_6_) *δ* (ppm): 36.2 (CH_3_), 106.5 (CH), 119.9 (CH), 124.9 (CH), 125.7 (C), 127.4 (2CH), 128.8 (2CH), 132.3 (CH, C), 139.2 (C), 145.2 (C), 167.4 (C). HRMS (ESI) calcd. for C_14_H_12_ClN_5_S [M+H]^+^: (*m*/*z*) = 318.0575; found: 318.0575 (Spectral Data: Supplementary Information. Figures S15–S18).

1-[(4-(4-Bromophenyl)-1,3-thiazol-2-yl)amino]-2-[(1-methyl-1*H*-imidazol-2-yl)methylidene]hydrazine (**5**) [49]: Yield: 86%. For crystallization: 0.510 g compound, 18 mL ethanol. M.p. 266–267 °C. ^1^H NMR (500 MHz, DMSO-*d*_6_) *δ* (ppm): 4.00 (s, 3H), 7.62 (t, *J* = 6.7 Hz, 3H), 7.75 (s, 1H), 7.83 (d, *J* = 7.9 Hz, 3H), 8.15 (s, 1H), 13.16 (s, 1H). ^13^C NMR (125 MHz, DMSO-*d*_6_) *δ* (ppm): 36.6 (CH_3_), 106.7 (CH), 119.9 (CH), 121.4 (CH), 125.9 (C), 127.8 (2CH), 131.9 (2CH), 133.4 (CH), 139.2 (C), 154.9 (C), 163.5 (C), 166.3 (C). HRMS (ESI) calcd. for C_14_H_12_BrN_5_S [M+H]^+^: (*m*/*z*) = 362.0070; found: 362.0074 (Spectral Data: Supplementary Information. Figures S19–S22).

1-[(4-(4-Cyanophenyl)-1,3-thiazol-2-yl)amino]-2-[(1-methyl-1*H*-imidazol-2-yl)methylidene]hydrazine (**6**) [49]: Yield: 84%. For crystallization: 0.434 g compound, 16 mL ethanol. M.p. 289–290 °C. ^1^H NMR (500 MHz, DMSO-*d*_6_) *δ* (ppm): 3.96 (s, 3H), 7.32 (s, 1H), 7.79–7.83 (m, 4H), 7.98 (d, *J* = 8.1 Hz, 2H), 8.19 (s, 1H), 13.15 (s, 1H). ^13^C NMR (125 MHz, DMSO-*d*_6_) *δ* (ppm): 36.4 (CH_3_), 109.5 (C), 109.8 (CH), 118.9 (C), 119.4 (CH), 124.6 (CH), 125.5 (C), 126.2 (2CH), 132.6 (2CH), 137.9 (C), 138.8 (CH), 148.9 (C), 167.2 (C). HRMS (ESI) calcd. for C_15_H_12_N_6_S [M+H]^+^: (*m*/*z*) = 309.0917; found: 309.0917 (Spectral Data: Supplementary Information. Figures S23–S26).

1-[(4-(4-Methylphenyl)-1,3-thiazol-2-yl)amino]-2-[(1-methyl-1*H*-imidazol-2-yl)methylidene]hydrazine (**7**) [49]: Yield: 96%. For crystallization: 0.467 g compound, 17 mL ethanol. M.p. 232–234 °C. ^1^H NMR (500 MHz, DMSO-*d*_6_) *δ* (ppm): 2.33 (s, 3H), 4.00 (s, 3H), 7.22 (d, *J* = 9.1 Hz, 2H), 7.41 (s, 1H), 7.73 (t, *J* = 7.5 Hz, 3H), 7.81 (s, 1H), 8.17 (s, 1H), 13.04 (s, 1H). ^13^C NMR (125 MHz, DMSO-*d*_6_) *δ* (ppm): 20.7 (CH_3_), 36.5 (CH_3_), 104.7 (CH), 120.0 (CH), 122.9 (C), 125.9 (3CH), 129.7 (2CH), 131.7 (C), 137.0 (C), 139.2 (CH), 157.3 (C), 167.4 (C). HRMS (ESI) calcd. for C_15_H_15_N_5_S [M+H]^+^: (*m*/*z*) = 298.1121; found: 298.1121 (Spectral Data: Supplementary Information. Figures S27–S30).

1-[(4-(4-Methoxyphenyl)-1,3-thiazol-2-yl)amino]-2-[(1-methyl-1*H*-imidazol-2-yl)methylidene]hydrazine (**8**) [49]: Yield: 76%. For crystallization: 0.389 g compound, 14 mL ethanol. M.p. 246–248 °C. ^1^H NMR (500 MHz, DMSO-*d*_6_) *δ* (ppm): 3.80 (s, 3H), 3.99 (s, 3H), 6.99 (d, *J* = 9.0 Hz, 2H), 7.34 (s, 1H), 7.74 (s, 1H), 7.80 (d, *J* = 9.0 Hz, 3H), 8.15 (s, 1H), 13.11 (s, 1H). ^13^C NMR (125 MHz, DMSO-*d*_6_) *δ* (ppm): 36.0 (CH_3_), 55.0 (OCH_3_), 103.5 (CH), 114.2 (2CH), 119.6 (CH), 125.7 (2C), 126.9 (3CH), 138.9 (C, CH), 158.9 (C), 168.3 (C). HRMS (ESI) calcd. for C_15_H_15_N_5_OS [M+H]^+^: (*m*/*z*) = 314.1070; found: 314.1071 (Spectral Data: Supplementary Information. Figures S31–S34).

1-[(4-(4-Trifluoromethylphenyl)-1,3-thiazol-2-yl)amino]-2-[(1-methyl-1*H*-imidazol-2-yl)methylidene]hydrazine (**9**): Yield: 67%. For crystallization: 0.386 g compound, 14 mL ethanol. M.p. 260–262 °C. ^1^H NMR (500 MHz, DMSO-*d*_6_) *δ* (ppm): 3.97 (s, 3H), 7.46–7.76 (m, 4H), 7.84 (s, 1H), 8.06 (d, *J* = 8.5 Hz, 2H), 8.21 (s, 1H), 13.22 (s, 1H). ^13^C NMR (125 MHz, DMSO-*d*_6_) *δ* (ppm): 36.3 (CH_3_), 108.5 (CH), 119.4 (CH), 123.2 (C), 124.8 (2CH), 125.3 (CH), 125.4 (CH), 125.6 (CH), 126.1 (2C), 137.8 (C), 138.8 (CH), 149.3 (C), 167.2 (C). HRMS (ESI) calcd. for C_15_H_12_F_3_N_5_S [M+H]^+^: (*m*/*z*) = 352.0838; found: 352.0839 (Spectral Data: Supplementary Information. Figures S35–S38).

1-[(4-(4-Methylsulfonylphenyl)-1,3-thiazol-2-yl)amino]-2-[(1-methyl-1*H*-imidazol-2-yl)methylidene]hydrazine (**10**): Yield: %83. For crystallization: 0.491 g compound, 18 mL ethanol. M.p. 270–271 °C. ^1^H NMR (400 MHz, DMSO-*d*_6_) *δ* (ppm): 3.25 (s, 3H), 3.99 (s, 3H), 7.72–7.84 (m, 3H), 7.95–7.99 (m, 2H), 8.09–8.13 (m, 3H), 13.25 (s, 1H). ^13^C NMR (125 MHz, DMSO-*d*_6_) *δ* (ppm): 36.2 (CH_3_), 43.9 (SO_2_CH_3_), 109.1 (CH), 119.5 (CH), 124.8 (CH), 125.5 (C), 126.2 (2CH), 127.7 (2CH), 138.6 (C), 138.8 (CH), 139.5 (C), 150.2 (C), 167.3 (C). HRMS (ESI) calcd. for C_15_H_15_N_5_O_2_S_2_ [M+H]^+^: (*m*/*z*) = 362.0740; found: 362.0749 (Spectral Data: Supplementary Information. Figures S39–S42).

1-[(4-(Naphthalen-2-yl)-1,3-thiazol-2-yl)amino]-2-[(1-methyl-1*H*-imidazol-2-yl)methylidene]hydrazine (**11**): Yield: 65%. For crystallization: 0.355 g compound, 13 mL ethanol. M.p. 250–251 °C. ^1^H NMR (500 MHz, DMSO-*d*_6_) *δ* (ppm): 3.99 (s, 3H), 7.51 (p, *J* = 8.3 Hz, 7.1 Hz, 2H), 7.66 (s, 1H), 7.74 (s, 1H), 7.81 (s, 1H), 7.91 (d, *J* = 7.7 Hz, 1H), 7.94–7.97 (m, 2H), 8.02 (d, *J* = 8.4 Hz, 1H), 8.18 (m, 1H), 8.39 (s, 1H), 13.18 (s, 1H). ^13^C NMR (125 MHz, DMSO-*d*_6_) *δ* (ppm): 36.6 (CH_3_), 106.4 (CH), 119.3 (CH), 123.0 (CH), 123.8 (C), 124.3 (CH), 125.4 (CH), 126.2 (CH), 126.5 (CH), 127.6 (CH), 128.1 (CH), 128.2 (CH), 131.6 (C), 132.5 (CH), 133.1 (C), 138.9 (C), 150.7 (C), 167.1 (C). HRMS (ESI) calcd. for C_18_H_15_N_5_S [M+H]^+^: (*m*/*z*) = 334.1121; found: 334.1124 (Spectral Data: Supplementary Information. Figures S43–S46).

1-[(4-(Benzo[d][1,3]dioxol-5-yl)-1,3-thiazol-2-yl)amino]-2-[(1-methyl-1*H*-imidazol-2-yl)methylidene]hydrazine (**12**): Yield: 83%. For crystallization: 0.445 g compound, 16 mL ethanol. M.p. 257–258 °C. ^1^H NMR (500 MHz, DMSO-*d*_6_) *δ* (ppm): 4.01 (s, 3H), 6.07 (s, 2H), 6.97 (d, *J* = 9.1 Hz, 1H), 7.38–7.42 (m, 3H), 7.73 (s, 1H), 7.81 (s, 1H), 8.14 (s, 1H), 13.09 (s, 1H). ^13^C NMR (125 MHz, DMSO-*d*_6_) *δ* (ppm): 36.2 (CH_3_), 101.2 (CH), 102.9 (CH), 105.9 (CH), 108.7 (CH), 119.7 (CH), 123.6 (CH), 125.3 (2CH), 126.6 (C), 138.9 (C), 147.0 (CH), 148.1 (2C), 165.9 (C). HRMS (ESI) calcd. for C_15_H_13_N_5_O_2_S [M+H]^+^: (*m*/*z*) = 328.0863; found: 328.0866 (Spectral Data: Supplementary Information. Figures S47–S50).

1-[(4-(4-(Thiophen-2-yl)phenyl)-1,3-thiazol-2-yl)amino]-2-[(1-methyl-1*H*-imidazol-2-yl)methylidene]hydrazine (**13**): Yield: 89%. For crystallization: 0.532 g compound, 19 mL ethanol. M.p. 265–267 °C. ^1^H NMR (500 MHz, DMSO-*d*_6_) *δ* (ppm): 3.99 (s, 3H), 7.16–7.17 (m, 1H), 7.56–7.59 (m, 3H), 7.72–7.76 (m, 3H), 7.83 (s, 1H), 7.90–7.93 (m, 2H), 8.15 (s, 1H), 13.15 (s, 1H). ^13^C NMR (125 MHz, DMSO-*d*_6_) *δ* (ppm): 36.5 (CH_3_), 105.7 (CH), 119.5 (CH), 123.8 (CH), 125.4 (CH), 125.6 (2CH), 125.8 (CH), 126.3 (2CH), 128.7 (CH), 133.0 (2C), 139.0 (C), 142.8 (C), 143.0 (CH), 151.1 (C), 167.4 (C). HRMS (ESI) calcd. for C_18_H_15_N_5_S_2_ [M+H]^+^: (*m*/*z*) = 366.0842; found: 366.0852 (Spectral Data: Supplementary Information. Figures S51–S54).

### 4.2. Biologic Activity

#### 4.2.1. Cytotoxicity

A549 (ATCC, Manassas, VA, USA) and MCF-7 (ATCC, VA, USA) cells were grown in Dulbecco’s Modified Eagle Medium (DMEM; Wako, Osaka, Japan) containing 10% fetal bovine serum (FBS; Equitech-Bio, Kerrville, TX, USA). Jurkat T-cells (ATCC, VA, USA) were maintained in RPMI-1640 medium (Wako Pure Chemical Industries, Osaka, Japan) supplemented with 10% FBS, while PBMCs (Precision Bioservices, Frederick, MD, USA) were expanded in the same medium supplemented with 10% human AB serum (Gemini, Woodland, CA, USA). Streptomycin was added to all culture media at a final concentration of 89 µg/mL (Meiji Seika Pharma, Tokyo, Japan). All cells were kept at 37 °C under humidified conditions with 5% CO_2_. For viability measurements, A549 and MCF-7 cells were seeded into 24-well plates, whereas PBMCs were dispensed into 96-well plates (Iwaki, Asahi Glass, Chiba, Japan) at initial densities of 4 × 10^4^ cells/mL and 5 × 10^5^ cells/mL, respectively. After a 72 h pre-incubation period, cultures were treated with the synthesized compounds or with lapatinib (Sigma-Aldrich, St. Louis, MO, USA), included as the reference agent. Concentrated stock solutions were prepared in dimethyl sulfoxide (DMSO; Wako Pure Chemical Industries) and diluted with culture medium to final assay concentrations ranging from 0.1 to 10 mM. The DMSO content was kept constant at 1% in all wells. Cell viability was assessed using the MTT colorimetric assay (Dojindo, Kumamoto, Japan), following established procedures with minor protocol refinements previously reported in the literature [4,55].

#### 4.2.2. Apoptosis

A549 cells were treated with compound **13** at its IC_50_ concentration and incubated for 24 h. After exposure, cells were detached using 0.05% trypsin, rinsed twice with 1× binding buffer, and prepared for fluorescence-based apoptotic assessment. The resulting pellet was suspended in a staining mixture consisting of 50 µL of 1× binding buffer, along with 4 µL each of FITC-Annexin V, ethidium homodimer III, and Hoechst 33342. The suspension was incubated for 20 min at room temperature in the dark to ensure proper labeling. Following staining, cells were washed again with binding buffer, fixed in 2% paraformaldehyde, and briefly rinsed with phosphate-buffered saline (PBS). Apoptotic and necrotic populations were visualized and recorded using a Biorevo BZ-9000 fluorescence microscope (Keyence, Osaka, Japan), following procedures adapted from previously reported workflows [4,55].

#### 4.2.3. EGFR Inhibition

EGFR kinase activity was monitored using the Promega EGFR TK kit (Cat. No. V3831) at final test concentrations of 1 µM and 10 µM. HER-2 activity was assessed under analogous conditions using the Promega HER-2 TK kit (Cat. No. V9381) at 10 µM, in accordance with earlier studies [4,55]. For each assay, the enzyme and its substrate were diluted in a mixture containing 95 µL of 2.5× kinase buffer and 15 µL of 100 µM ATP. Reactions were assembled in 384-well plates by dispensing 4 µL of each compound solution (0.1–100 µM) into wells containing 8 µL of the enzyme/ATP/substrate preparation. Plates were incubated for 1.5 h at room temperature, after which ADP formation, reflecting kinase turnover, was quantified using the ADP-Glo™ detection system (Promega, Madison, WI, USA). Luminescence was measured with an Infinite M1000 reader (Tecan, Grödig, Austria). The inhibitory profiles of compounds **5** and **13** were compared with lapatinib, which was tested under the same conditions at 1 µM and 10 µM.

#### 4.2.4. In Vitro ALR2 Inhibition Assay

The ALR2 inhibitory properties of compounds **1**–**13** were assessed using a UV-visible assay tracking the decline in NADPH absorbance during the enzymatic reduction of DL-glyceraldehyde. The protocol followed earlier steady-state kinetic studies with minor adjustments [56,57,58]. Measurements were performed at 37 °C in 10 mM sodium phosphate buffer (pH 7.4) in 1 mL quartz cuvettes (1 cm path length). Each reaction mixture contained 0.11 mM NADPH and DL-glyceraldehyde at final concentrations between 0.071 and 0.376 mM. Test compounds were introduced from DMSO stock solutions, and the total DMSO content was kept below 1% (*v*/*v*).

Prior to substrate addition, the enzyme and inhibitor were combined and left on ice for 1 min to allow initial complex formation. The reaction was initiated by adding DL-glyceraldehyde, and the decrease in absorbance at 340 nm (ε_340_ = 6.22 mM^−1^ cm^−1^) was recorded for 180 s using a calibrated UV-Vis instrument. EPR served as the reference ALR2 blocker and was tested under identical conditions. All values represent the mean of triplicate determinations, and the entire experiment was repeated on three separate days (n = 3). Reaction rates were calculated from the linear segment of each kinetic trace.

**Figure 18:**
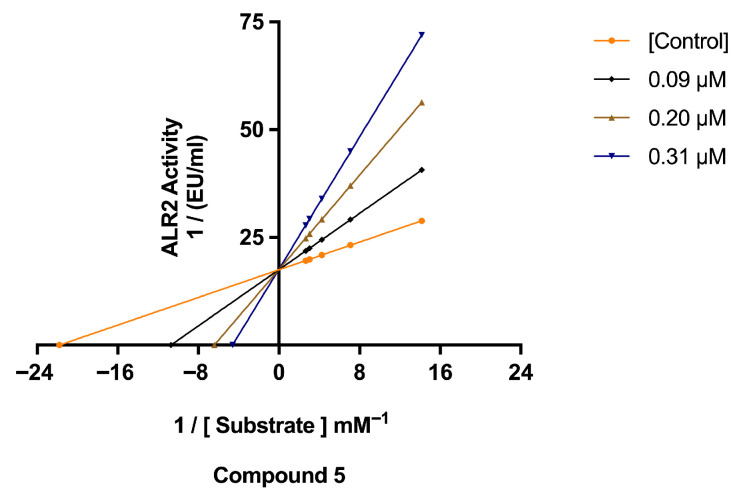
Lineweaver–Burk plots (1/v vs. 1/[S]) illustrating ALR2-catalyzed reduction of DL-glyceraldehyde in the absence of inhibitor and in the presence of compound **5** at three increasing concentrations (I_1_–I_3_). The set of lines intersecting along the *y*-axis reflects a competitive inhibition profile toward DL-glyceraldehyde.

For selected compounds, including compound **5**, extended kinetic analyses were conducted by varying both substrate and inhibitor levels. The resulting Lineweaver-Burk plots (1/v vs. 1/[S]) were used to interpret the inhibition pattern (Figure 18). Secondary replots were used to extract *K*_I_ values by linear regression [59,60]. Data processing and curve fitting were performed in GraphPad Prism (v10.0), and kinetic parameters are expressed as mean ± SEM.

### 4.3. In Silico Studies

#### 4.3.1. Protein and Ligand Preparation

The crystallographic coordinates used in the computational workflow were retrieved from the Protein Data Bank: EGFR (PDB ID: 3POZ) [50] and ALR2 (PDB ID: 1Z3N) [52]. Both structures were processed with the Protein Preparation Wizard in Maestro (Schrödinger Release 2025-1). The refinement protocol consisted of three sequential steps: structural correction, optimization of protonation states, and restrained minimization. During the initial phase, missing residues and incomplete side chains were rebuilt, hydrogen atoms were added, and bond orders were verified. Water molecules located more than 5 Å from the native ligand or from catalytically relevant residues were discarded, whereas waters positioned near the binding region were retained when they were likely to mediate interactions. Ionizable residues were assigned protonation states appropriate for physiological conditions (pH 7.4 ± 2.0). The hydrogen-bonding network was then optimized, followed by a restrained minimization using the OPLS4 force field until heavy-atom deviations stabilized near 0.30 Å, ensuring local strain relief while preserving the backbone geometry [61].

Ligands were processed using the LigPrep module (Schrödinger Release 2025-1). Protonation and tautomeric forms were generated with Epik v7.1 at pH 7.4 ± 2.0. Counterions were removed, salts were neutralized, and stereochemical variants were limited to one configuration per chiral center. All structures were then minimized under the OPLS4 force field to obtain low-energy conformers for downstream modeling.

#### 4.3.2. Receptor Grid Generation and Molecular Docking

Grid generation was carried out in Maestro using the Glide receptor-grid module (Schrödinger Release 2025-1). The binding region was centered on the crystallographic ligand from each structure (lapatinib in 3POZ and lidorestat in 1Z3N) to ensure alignment with the experimentally determined active sites. A cubic grid encompassing residues within ~10 Å of the bound ligand was generated, and the outer box dimensions (~20 × 20 × 20 Å) were defined to enable broad sampling during docking. To account for modest protein flexibility, the van der Waals radii of nonpolar atoms were scaled uniformly (scaling factor 1.0; partial-charge cutoff 0.25). After the pocket was defined, the co-crystallized ligand was removed but its orientation was retained as a geometric reference.

Docking simulations were performed with Glide in Extra-Precision (XP) mode. The receptor was kept rigid, while ligands were fully flexible. No geometric or energetic constraints were applied. All docking poses were refined through Glide’s post-docking minimization routine and scored using the GlideScore function [62,63].

#### 4.3.3. Binding Free Energy Calculations (Prime MM-GBSA)

Prime MM-GBSA calculations (Schrödinger Release 2025-1) were applied to estimate the binding free energies of the best-ranked docking poses. The methodology described in [64] was followed. Energies were computed for the minimized complex, the isolated receptor, and the free ligand using the OPLS4 force field. Solvent contributions were modeled with the VSGB 2.0 implicit solvent model. Before energy evaluation, partial minimization of the complex was performed to relax the ligand and the surrounding residues within the binding region.

Binding free energy (Δ*G*_bind_) was calculated as:

$$\Delta G_{\mathrm{bind}}=E_{\mathrm{complex}}-\left(E_{\mathrm{protein}}+E_{\mathrm{ligand}}\right)$$

The resulting Δ*G*_bind_ values supplemented docking results by offering thermodynamic insight into the predicted binding affinities.

#### 4.3.4. IFD

IFD (Schrödinger Release 2025-1) commenced from the top-ranked Glide XP pose of each ligand. Residues located within 5 Å of the ligand were refined using Prime, permitting side-chain reorientation and limited backbone displacement to alleviate steric conflicts. During this adjustment step, ligand coordinates remained fixed. After refinement, ligands were redocked into the adapted active site using Glide XP without applying positional constraints.

Each solution was scored using the standard IFD scoring function, which integrates GlideScore and Prime energy components to evaluate both ligand accommodation and protein reorganization [65]. Generated poses were examined for improvements relative to rigid docking, with emphasis on altered residue interactions and conformational compatibility.

#### 4.3.5. MD Simulations

MD simulations were performed using the Desmond (Schrödinger Release 2025-1). Each ligand-protein complex was embedded in an orthorhombic simulation box solvated with TIP3P water molecules. Charge neutrality was achieved using Na^+^ or Cl^−^ counterions, followed by adjustment to physiological ionic strength. Initial minimization was performed to remove steric clashes. Production simulations were run under NPT conditions at 300 K and 1 atm, parameterized by the OPLS4 force field. Trajectory analysis included backbone RMSD, residue-level RMSF, and interaction-frequency mapping, offering detailed information on conformational stability and persistent residue contacts over the simulation course [66].

#### 4.3.6. In Silico Pharmacokinetic Studies

Pharmacokinetic and drug-likeness properties of compounds **5** and **13** were predicted using SwissADME [53]. Calculated descriptors included lipophilicity, aqueous solubility, passive permeability, cytochrome P450 inhibition profiles, P-gp substrate status, and estimated oral bioavailability. These parameters provided an initial assessment of the suitability of both molecules as drug-like small-molecule candidates.

## 5. Conclusions

In this work, a series of hydrazonylthiazole derivatives (**1**–**13**) was synthesized and examined to determine their ability to interfere with EGFR- and ALR2-linked mechanisms that contribute to NSCLC and breast cancer biology. Among these molecules, compounds **5** and **13** repeatedly ranked as the most active, suppressing the growth of A549 and MCF-7 cells while exerting limited effects on PBMCs, indicating a favorable level of selectivity. Compound **13**, disclosed here for the first time, produced a clear apoptotic response in A549 cells and showed strong suppression of EGFR activity. These outcomes were in line with the computational results: IFD and extended MD simulations all pointed to a tightly accommodated ligand, persistent contacts with essential residues, and a binding arrangement well-suited to the EGFR pocket. Compound **5**, in contrast, demonstrated modest influence on EGFR yet proved to be the most efficient ALR2 binder of the series. Its interaction pattern within ALR2, supported by IFD and MD analyses, showed a stable orientation and consistent engagement with catalytic residues.

## Figures and Tables

**Table 1:** IC_50_ values and selectivity indices for compounds **5** and **13**.

Compound	IC_50_ Values (μM)				SI *
	A549 Cells	MCF-7 Cells	Jurkat Cells	PBMCs	
**5**	6.87 ± 2.10	2.78 ± 2.85	1.94 ± 0.65	72.53 ± 14.02	37.39
**13**	1.33 ± 0.41	1.74 ± 0.38	1.65 ± 0.40	229.18 ± 26.16	138.90
Lapatinib	17.95 ± 5.12	8.43 ± 2.85	1.35 ± 0.41	9.79 ± 2.69	7.25

* Selectivity Index (SI) = IC_50_ for PBMC/IC_50_ for Jurkat cell line.

**Table 2:** ALR2 inhibition (*K*_I_) for compounds **1**–**13**; mean ±SEM (*n* = 3). EPR as reference drug.

Compound	*K*_I_ (µM)	*R* ^2^
**1**	2.64 ± 0.16	0.9879
**2**	1.83 ± 0.11	0.9881
**3**	0.44 ± 0.09	0.9882
**4**	0.28 ± 0.03	0.9883
**5**	0.08 ± 0.01	0.9884
**6**	0.21 ± 0.02	0.9881
**7**	0.28 ± 0.03	0.9891
**8**	0.21 ± 0.02	0.9889
**9**	0.91 ± 0.05	0.9881
**10**	0.24 ± 0.02	0.9877
**11**	1.91 ± 0.10	0.9878
**12**	2.01 ± 0.10	0.9882
**13**	2.48 ± 0.14	0.9883
**EPR** *	0.86 ± 0.06	0.9853

* EPR: Epalrestat.

**Table 3:** Docking scores, IFD scores, and MM-GBSA energies for compounds **5**, **13**, and lapatinib in the EGFR and ALR2 active sites.

Compound	EGFR			ALR2		
	Docking Score (kcal/mol)	IFD-Score (kcal/mol)	MM-GBSA Δ*G*_bind_ (kcal/mol)	Docking Score (kcal/mol)	IFD-Score (kcal/mol)	MM-GBSA Δ*G*_bind_ (kcal/mol)
**5**	−10.68	−627.47	−44.11	−10.25	−691.12	−35.76
**13**	−10.92	−628.31	−41.75	-	-	-
Lapatinib	−13.54	−638.87	−90.38	-	-	-

**Table 4:** SwissADME-predicted physicochemical and pharmacokinetic properties of compounds **5** and **13**.

Parameter	Compound 5	Compound 13
Topological Polar Surface Area (TPSA, Å^2^)	83.34	111.58
Consensus Log P_o/w_	3.14	3.87
Solubility (Log S, ESOL)	−4.70	−5.13
Solubility (Log S, SILICOS-IT)	−5.68	−6.61
GI Absorption	High	High
BBB Permeant	No	No
P-gp Substrate	No	No
Inhibition		
CYP1A2	Yes	Yes
CYP2C19	Yes	Yes
CYP2C9	Yes	Yes
CYP2D6	No	Yes
CYP3A4	Yes	Yes
Lipinski Rule	0	0
Bioavailability Score	0.55	0.55
PAINS Alert	0	0
Synthetic Accessibility	3.11	3.40

## Data Availability

Data are contained within the article and Supplementary Materials.

## Supplementary Materials

The following supporting information can be downloaded at: https://www.mdpi.com/article/10.3390/ph19010050/s1, Figure S1. ^1^H NMR Spectrum of **A**; Figure S2. ^13^C NMR Spectrum of **A**; Figure S3. ^1^H NMR Spectrum of compound **1** (0–15 ppm); Figure S4. ^1^H NMR Spectrum of compound **1** (7–9 ppm); Figure S5. ^13^C NMR Spectrum of compound **1**; Figure S6. Mass Spectrum of compound **1**; Figure S7. ^1^H NMR Spectrum of compound **2** (0–15 ppm); Figure S8. ^1^H NMR Spectrum of compound **2** (7–9 ppm); Figure S9. ^13^C NMR Spectrum of compound **2**; Figure S10. Mass Spectrum of compound **2**; Figure S11. ^1^H NMR Spectrum of compound **3** (0–15 ppm); Figure S12. ^1^H NMR Spectrum of compound **3** (7–9 ppm); Figure S13. ^13^C NMR Spectrum of compound **3**; Figure S14. Mass Spectrum of compound **3**; Figure S15. ^1^H NMR Spectrum of compound **4** (0–15 ppm); Figure S16. ^1^H NMR Spectrum of compound **4** (7–9 ppm); Figure S17. ^13^C NMR Spectrum of compound **4**; Figure S18. Mass Spectrum of compound **4**; Figure S19. ^1^H NMR Spectrum of compound **5** (0–15 ppm); Figure S20. ^1^H NMR Spectrum of compound **5** (7–9 ppm); Figure S21. ^13^C NMR Spectrum of compound **5**; Figure S22. Mass Spectrum of compound **5**; Figure S23. ^1^H NMR Spectrum of compound **6** (0–15 ppm); Figure S24. ^1^H NMR Spectrum of compound **6** (7–9 ppm); Figure S25. ^13^C NMR Spectrum of compound **6**; Figure S26. Mass Spectrum of compound **6**; Figure S27. ^1^H NMR Spectrum of compound **7** (0–15 ppm); Figure S28. ^1^H NMR Spectrum of compound **7** (7–9 ppm); Figure S29. ^13^C NMR Spectrum of compound **7**; Figure S30. Mass Spectrum of compound **7**; Figure S31. ^1^H NMR Spectrum of compound **8** (0–15 ppm); Figure S32. ^1^H NMR Spectrum of compound **8** (7–9 ppm); Figure S33. ^13^C NMR Spectrum of compound **8**; Figure S34. Mass Spectrum of compound **8**; Figure S35. ^1^H NMR Spectrum of compound **9** (0–15 ppm); Figure S36. ^1^H NMR Spectrum of compound **9** (7–9 ppm); Figure S37. ^13^C NMR Spectrum of compound **9**; Figure S38. Mass Spectrum of compound **9**; Figure S39: ^1^H NMR Spectrum of compound **10** (0–15 ppm); Figure S40: ^1^H NMR Spectrum of compound **10** (7–9 ppm); Figure S41. ^13^C NMR Spectrum of compound **10**; Figure S42. Mass Spectrum of compound **10**; Figure S43. ^1^H NMR Spectrum of compound **11** (0–15 ppm); Figure S44. ^1^H NMR Spectrum of compound **11** (7–9 ppm); Figure S45. ^13^C NMR Spectrum of compound **11**; Figure S46. Mass Spectrum of compound **11**; Figure S47. ^1^H NMR Spectrum of compound **12** (0–15 ppm); Figure S48. ^1^H NMR Spectrum of compound **12** (7–9 ppm); Figure S49. ^13^C NMR Spectrum of compound **12**; Figure S50. Mass Spectrum of compound **12**; Figure S51. ^1^H NMR Spectrum of compound **13** (0–15 ppm); Figure S52. ^1^H NMR Spectrum of compound **13** (7–9 ppm); Figure S53. ^13^C NMR Spectrum of compound **13**; Figure S54. Mass Spectrum of compound **13**; Table S1. Prime MM-GBSA binding free energy (Δ*G*_bind_) and energetic decomposition (kcal/mol) at the EGFR and AR binding sites.
