# Supplementary material for: Hydrazonylthiazole Derivatives as Dual EGFR and ALR2 Inhibitors: Design, Synthesis, and Comprehensive In Vitro and In Silico Evaluation for Potential Anticancer Activity
Source: Pharmaceuticals (Basel). 2025 Dec 25;19(1):50. doi: 10.3390/ph19010050 (PMC12845312; doi:10.3390/ph19010050)
Supplement: Supplementary file 1 [file pharmaceuticals-19-00050-s001.zip › pharmaceuticals-4039624-supplementary.pdf]

Supplementary Information

Hydrazonylthiazole Derivatives as Dual EGFR and ALR2 Inhibitors: Design, Synthesis, and Comprehensive In Vitro and In Silico Evaluation for Potential Anticancer Activity

Belgin Sever [1,2], Cüneyt Türkeş [3,*], Yeliz Demir [4], Khaled M. Elamin [5], Wadah Osman [6], Kübra Oral [1], Selenay Akıncı Genç [1], Zerrin Cantürk [7], Takuya Masunaga [2], Naoki Kishimoto [5,8], Shogo Misumi [5,8], Masami Otsuka [2,9], Mikako Fujita [2] and Halilibrahim Ciftci [2,9,10,*]

[1] Department of Pharmaceutical Chemistry, Faculty of Pharmacy, Anadolu University, Eskisehir 26470, Türkiye; belginsever@anadolu.edu.tr (B.S.); kbrorl78@gmail.com (K.O.); selenayakinci@gmail.com (S.A.G.)
[2] Medicinal and Biological Chemistry Science Farm Joint Research Laboratory, Faculty of Life Sciences, Kumamoto University, Kumamoto 862-0973, Japan; tmasunaga@kumamoto-u.ac.jp (T.M.); motsuka@gpo.kumamoto-u.ac.jp (M.O.); mfujita@kumamoto-u.ac.jp (M.F.)
[3] Department of Biochemistry, Faculty of Pharmacy, Erzincan Binali Yıldırım University, Erzincan 24100, Türkiye; cuneyt.turkes@erzincan.edu.tr (C.T.)
[4] Department of Pharmacy Services, Nihat Delibalta Göle Vocational High School, Ardahan University, Ardahan 75700, Türkiye; yelizdemir@ardahan.edu.tr (Y.D.)
[5] Global Center for Natural Products Research, Faculty of Life Sciences, Kumamoto University, 5-1 Oe-honmachi, Chuo-ku, Kumamoto 862-0973, Japan; khaled@kumamoto-u.ac.jp (K.M.E.); naokishi@kumamoto-u.ac.jp (N.K.); misumi@gpo.kumamoto-u.ac.jp (S.M.)
[6] Department of Pharmacognosy, Faculty of Pharmacy, Prince Sattam Bin Abdulaziz University, Al-kharj 11942, Saudi Arabia, w.osman@psau.edu.sa (W.O.)
[7] Department of Pharmaceutical Microbiology, Faculty of Pharmacy, Anadolu University, Eskişehir 26470, Türkiye; zkcanturk@anadolu.edu.tr (Z.C.)
[8] Department of Environmental and Molecular Health Sciences, Faculty of Medical and Pharmaceutical Sciences, Kumamoto University, Kumamoto 862-0973, Japan
[9] Department of Drug Discovery, Science Farm Ltd., Kumamoto 862-0976, Japan
[10] Department of Molecular Biology and Genetics, Burdur Mehmet Akif Ersoy University, Istiklal Campus, Burdur 15200, Türkiye; hciftci@mehmetakif.edu.tr (H.C.)
* Correspondence: hciftci@mehmetakif.edu.tr (H.C.); cuneyt.turkes@erzincan.edu.tr (C.T.)

Supplementary Figures

Figure S1. ^{1}H NMR Spectrum of **A**

Figure S2. ^{13}C NMR Spectrum of **A**

Figure S3. ^{1}H NMR Spectrum of compound **1** (0-15 ppm)

Figure S4. ^{1}H NMR Spectrum of compound **1** (7-9 ppm)

Figure S5. ^{13}C NMR Spectrum of compound **1**

Figure S6. Mass Spectrum of compound **1**

Figure S7. ^{1}H NMR Spectrum of compound **2** (0-15 ppm)

Figure S8. ^{1}H NMR Spectrum of compound **2** (7-9 ppm)

Figure S9. ^{13}C NMR Spectrum of compound **2**

Figure S10. Mass Spectrum of compound **2**

Figure S11. ^{1}H NMR Spectrum of compound **3** (0-15 ppm)

Figure S12. ^{1}H NMR Spectrum of compound **3** (7-9 ppm)

Figure S13.^{13}C NMR Spectrum of compound **3**

Figure S14. Mass Spectrum of compound **3**

Figure S15. ^{1}H NMR Spectrum of compound **4** (0-15 ppm)

Figure S16. ^{1}H NMR Spectrum of compound **4** (7-9 ppm)

Figure S17. ^{13}C NMR Spectrum of compound **4**

Figure S18. Mass Spectrum of compound **4**

Figure S19. ^{1}H NMR Spectrum of compound **5** (0-15 ppm)

Figure S20. ^{1}H NMR Spectrum of compound **5** (7-9 ppm)

Figure S21. ^{13}C NMR Spectrum of compound **5**

Figure S22. Mass Spectrum of compound **5**

Figure S23. ^{1}H NMR Spectrum of compound **6** (0-15 ppm)

Figure S24. ^{1}H NMR Spectrum of compound **6** (7-9 ppm)

Figure S25. ^{13}C NMR Spectrum of compound **6**

Figure S26. Mass Spectrum of compound **6**

Figure S27. ^{1}H NMR Spectrum of compound **7** (0-15 ppm)

Figure S28. ^{1}H NMR Spectrum of compound **7** (7-9 ppm)

Figure S29. ^{13}C NMR Spectrum of compound **7**

Figure S30. Mass Spectrum of compound **7**

Figure S31. ^{1}H NMR Spectrum of compound **8** (0-15 ppm)

Figure S32. ^{1}H NMR Spectrum of compound **8** (7-9 ppm)

Figure S33. ^{13}C NMR Spectrum of compound **8**

Figure S34. Mass Spectrum of compound **8**

Figure S35. ^{1}H NMR Spectrum of compound **9** (0-15 ppm)

Figure S36. ^{1}H NMR Spectrum of compound **9** (7-9 ppm)

Figure S37. ^{13}C NMR Spectrum of compound **9**

Figure S38. Mass Spectrum of compound **9**

Figure S39: ^{1}H NMR Spectrum of compound **10** (0-15 ppm)

Figure S40: ^{1}H NMR Spectrum of compound **10** (7-9 ppm)

Figure S41. ^{13}C NMR Spectrum of compound **10**

Figure S42. Mass Spectrum of compound **10**

Figure S43. ^{1}H NMR Spectrum of compound **11** (0-15 ppm)

Figure S44. ^{1}H NMR Spectrum of compound **11** (7-9 ppm)

Figure S45. ^{13}C NMR Spectrum of compound **11**

Figure S46. Mass Spectrum of compound **11**

Figure S47. ^{1}H NMR Spectrum of compound **12** (0-15 ppm)

Figure S48. ^{1}H NMR Spectrum of compound **12** (7-9 ppm)

Figure S49. ^{13}C NMR Spectrum of compound **12**

Figure S50. Mass Spectrum of compound **12**

Figure S51. ^{1}H NMR Spectrum of compound **13** (0-15 ppm)

Figure S52. ^{1}H NMR Spectrum of compound **13** (7-9 ppm)

Figure S53. ^{13}C NMR Spectrum of compound **13**

Figure S54. Mass Spectrum of compound **13**

Supplementary Table

Table S1. Prime MM-GBSA binding free energy (ΔG_{bind}) and energetic decomposition (kcal/mol) at the EGFR and AR binding sites

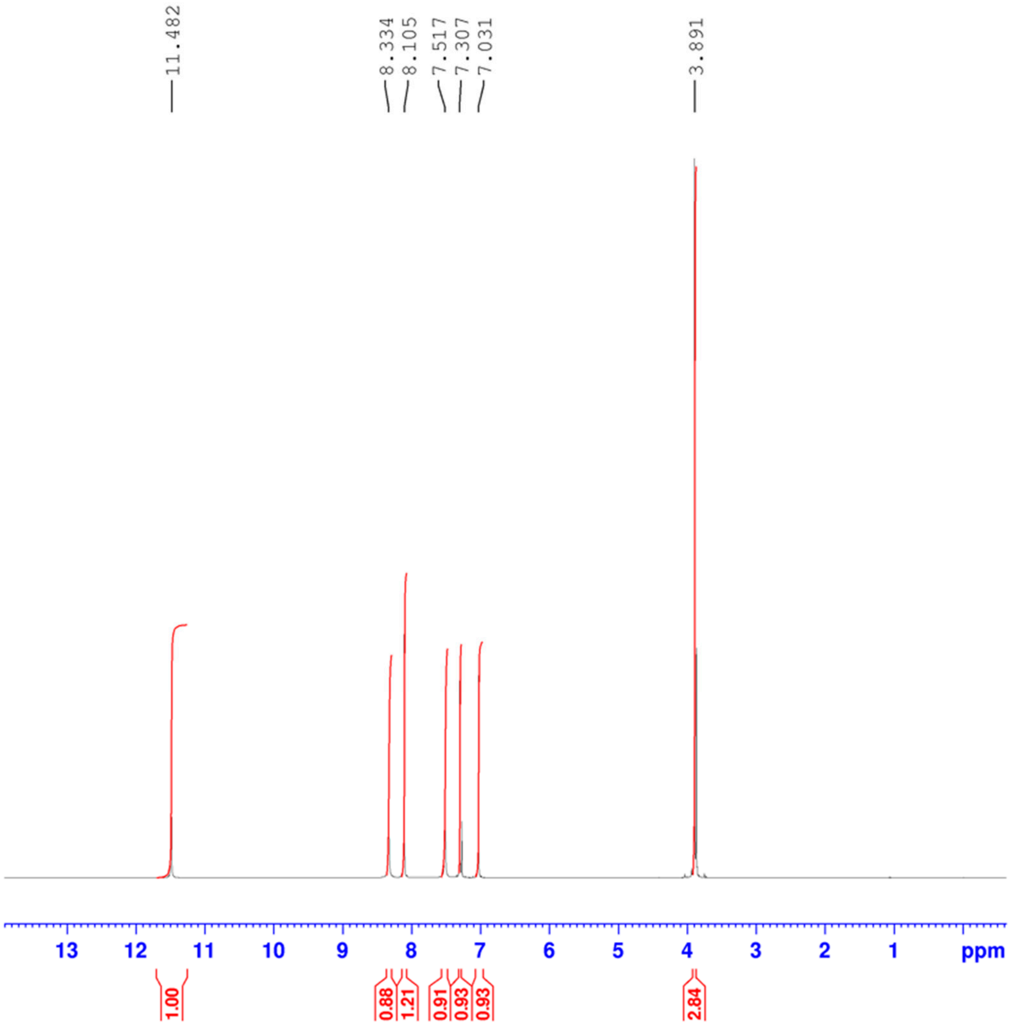

Figure S1. ^{1}H NMR Spectrum of **A**

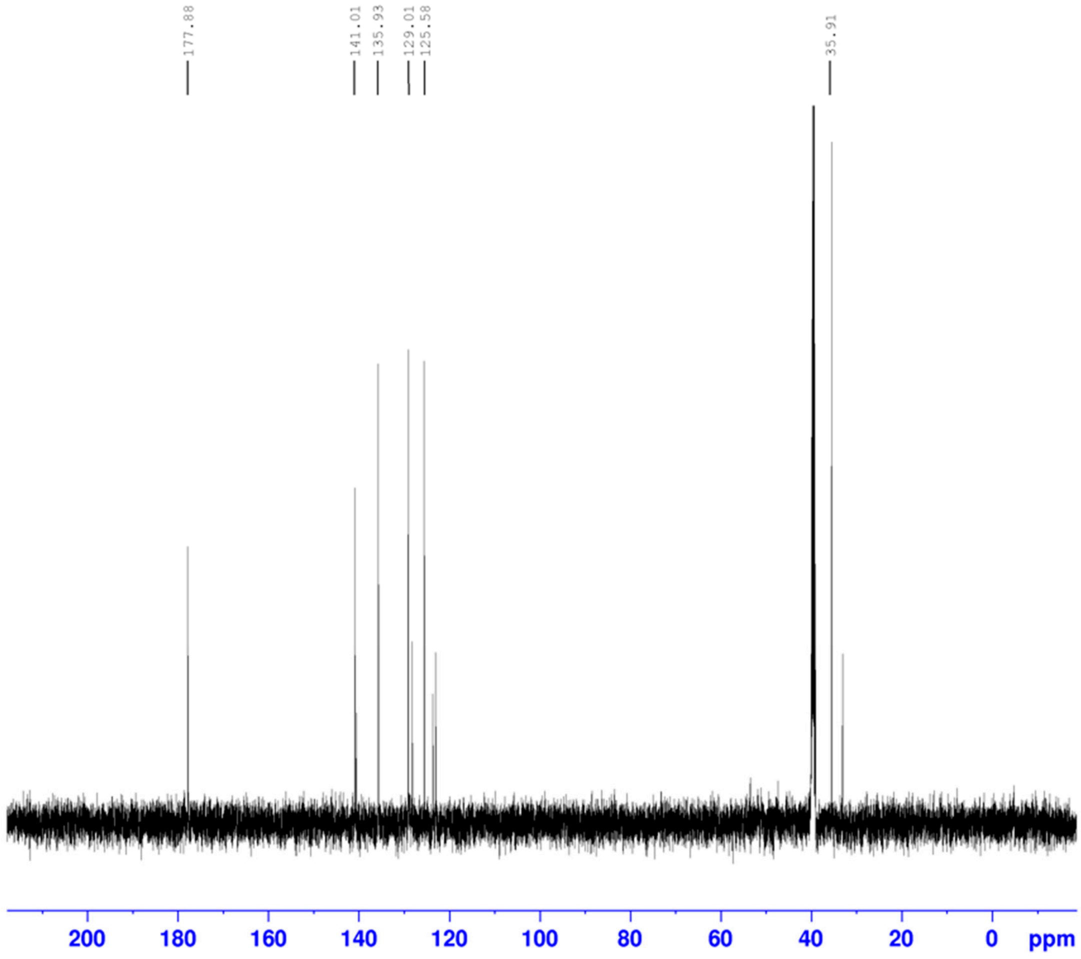

Figure S2. ^{13}C NMR Spectrum of **A**

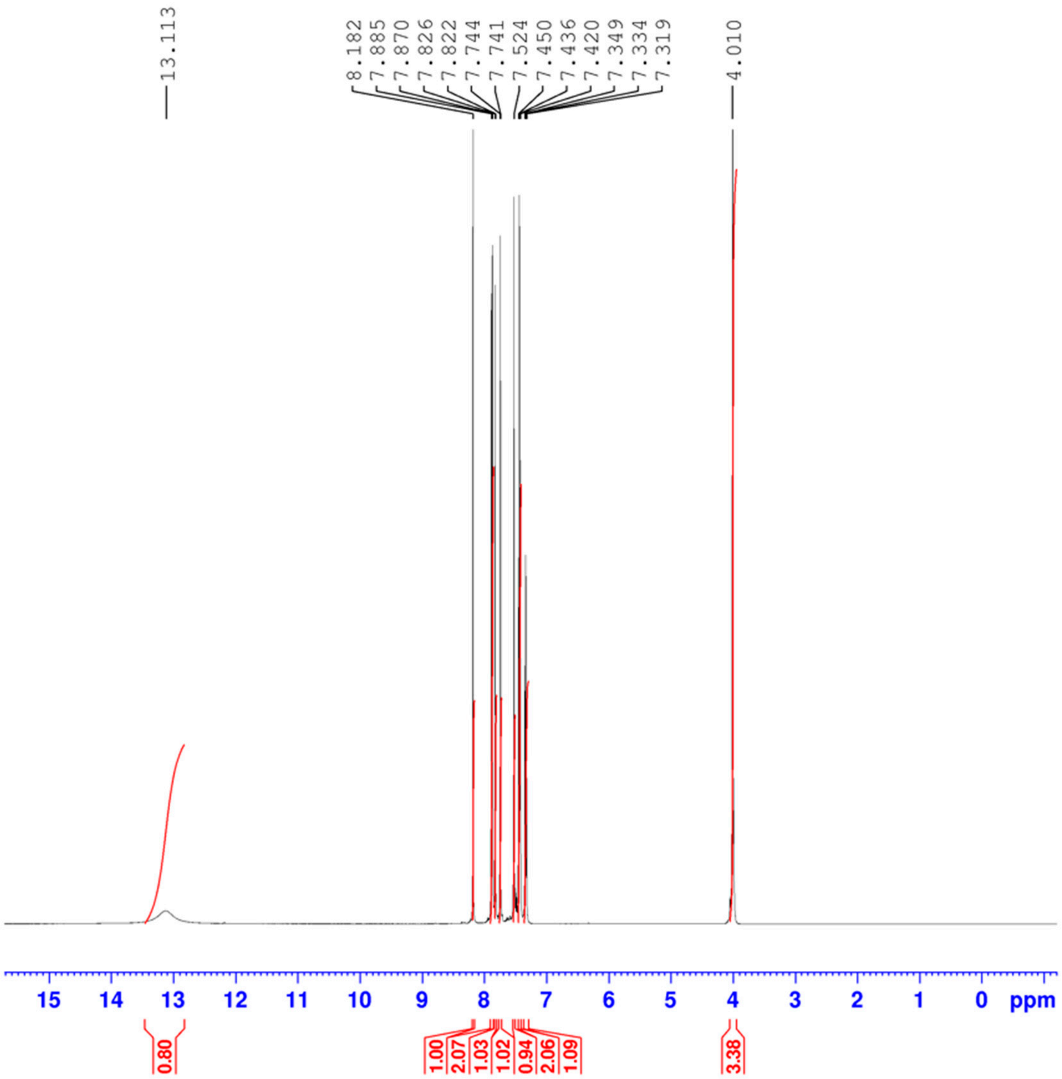

Figure S3. ^{1}H NMR Spectrum of compound **1** (0-15 ppm)

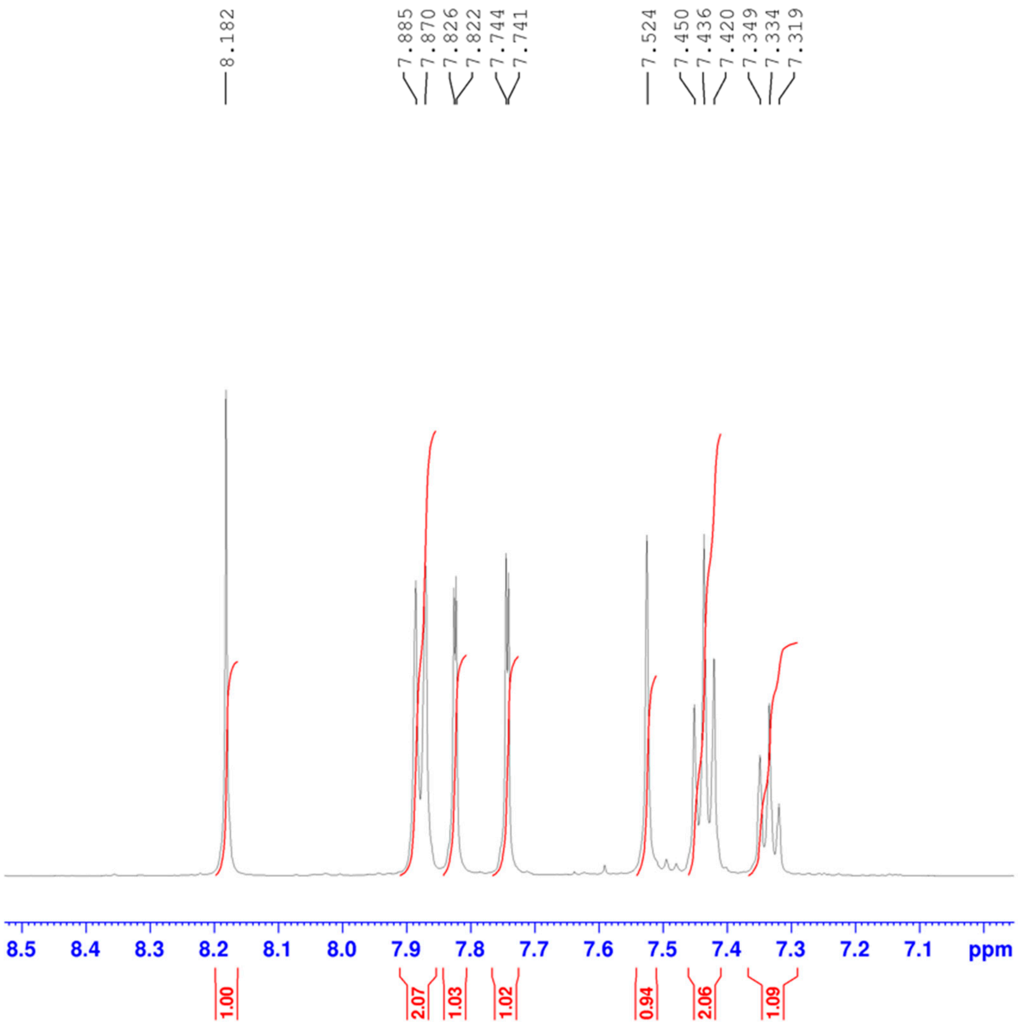

Figure S4. ^{1}H NMR Spectrum of compound **1** (7-9 ppm)

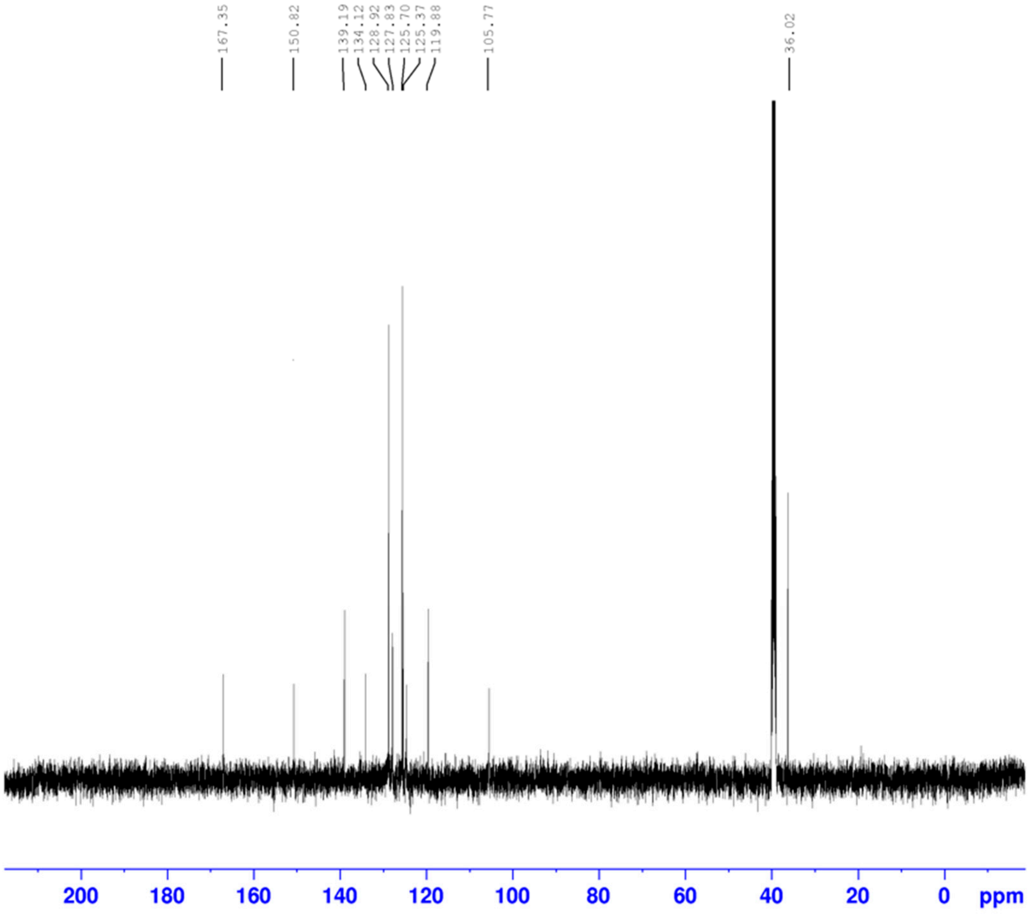

Figure S5. ^{13}C NMR Spectrum of compound **1**

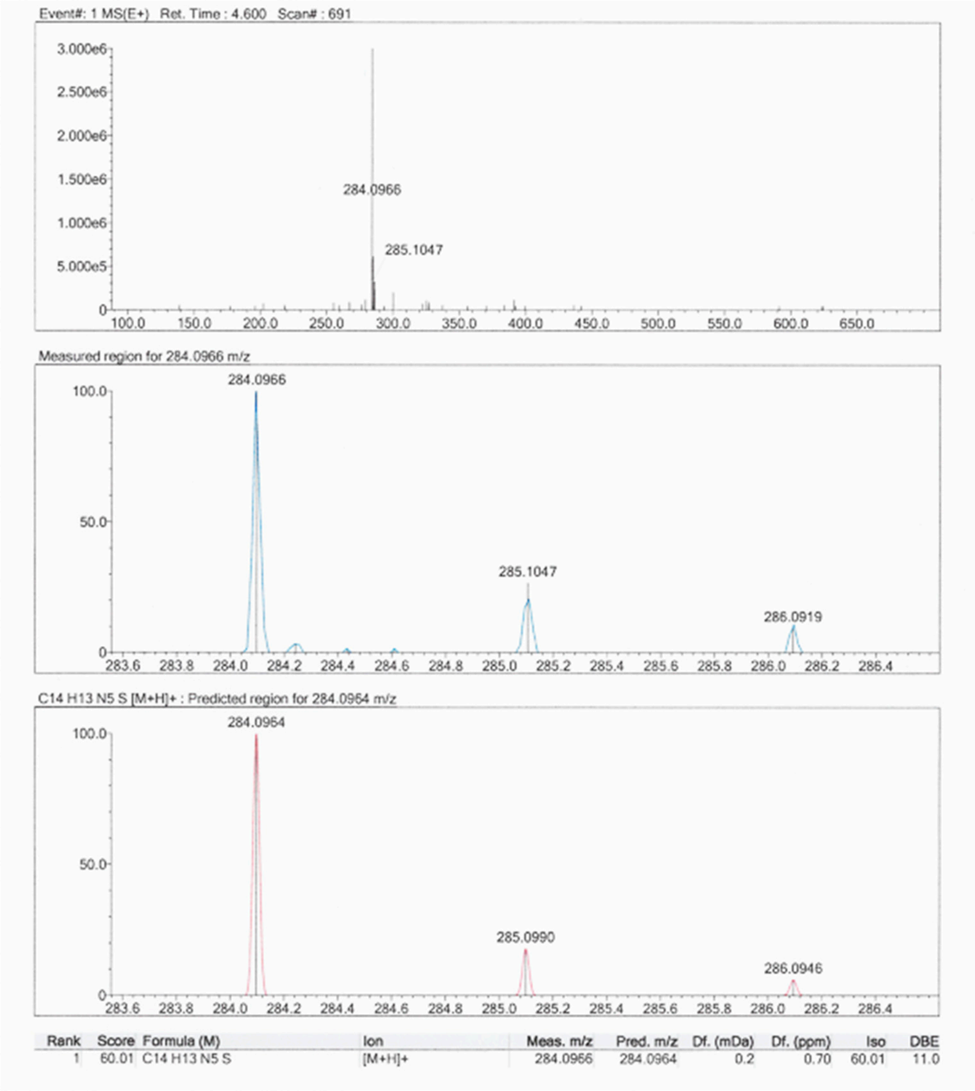

Rank	Score	Formula (M)	Ion	Meas. m/z	Pred. m/z	Df. (mDa)	Df. (ppm)	Iso	DBE
1	60.01	C14 H13 N5 S	[M+H]+	284.0966	284.0964	0.2	0.70	60.01	11.0

Figure S6. Mass Spectrum of compound **1**

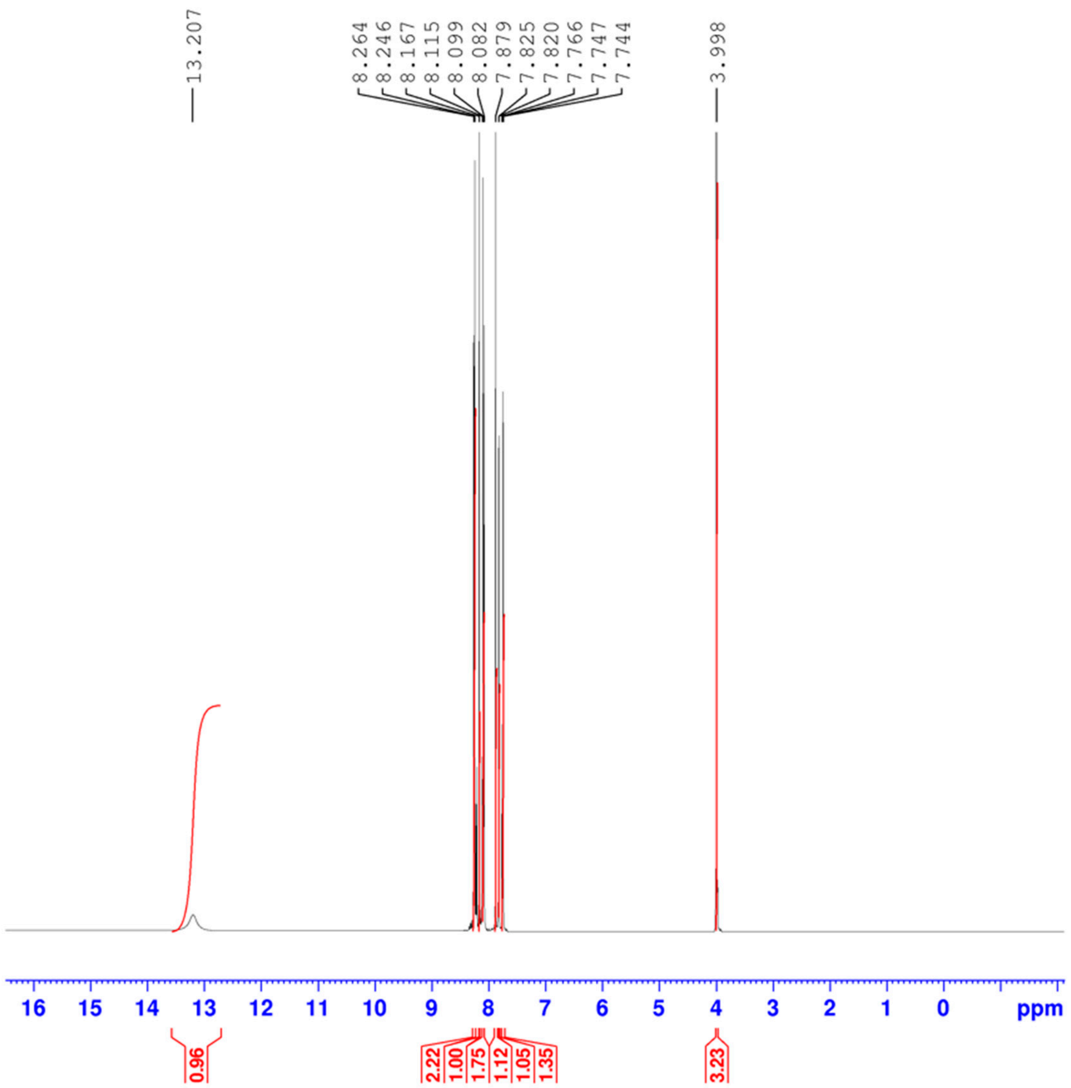

Figure S7. ^{1}H NMR Spectrum of compound **2** (0-15 ppm)

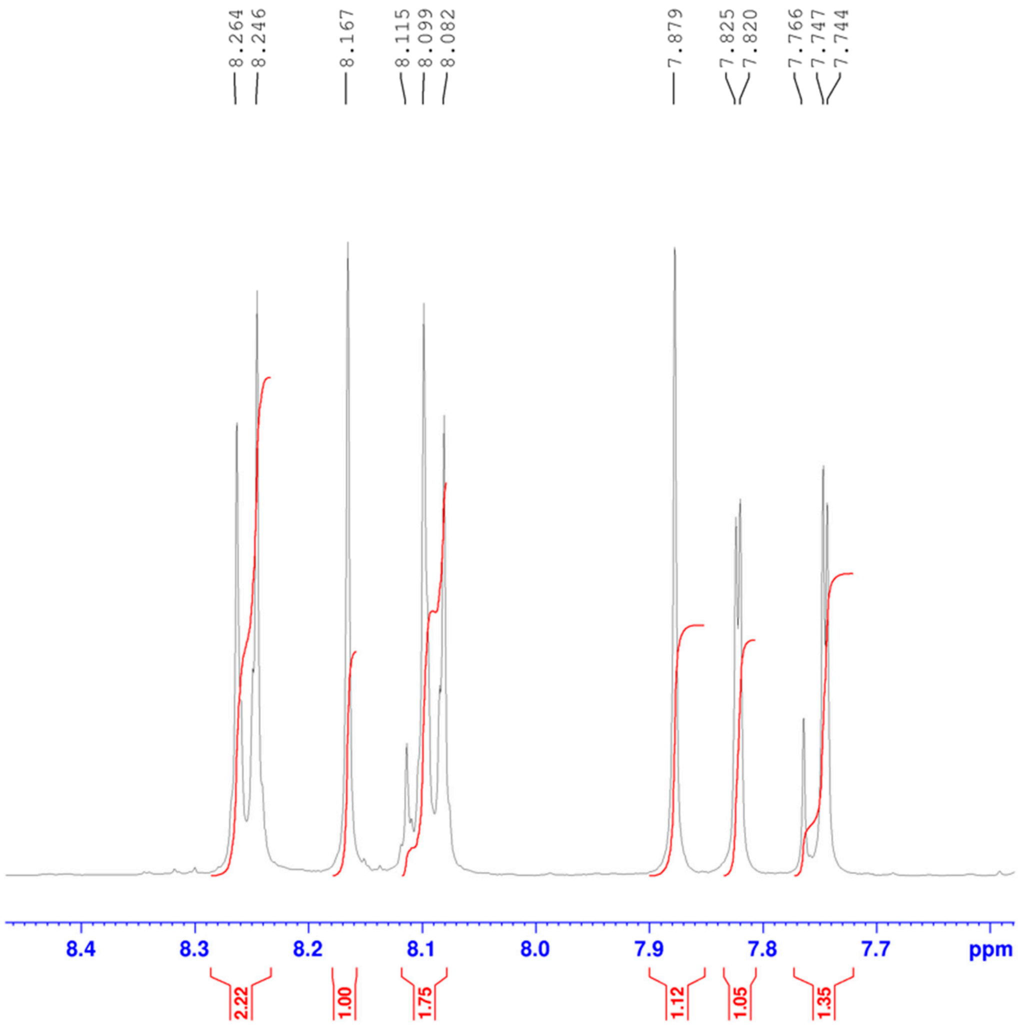

Figure S8. ^{1}H NMR Spectrum of compound **2** (7-9 ppm)

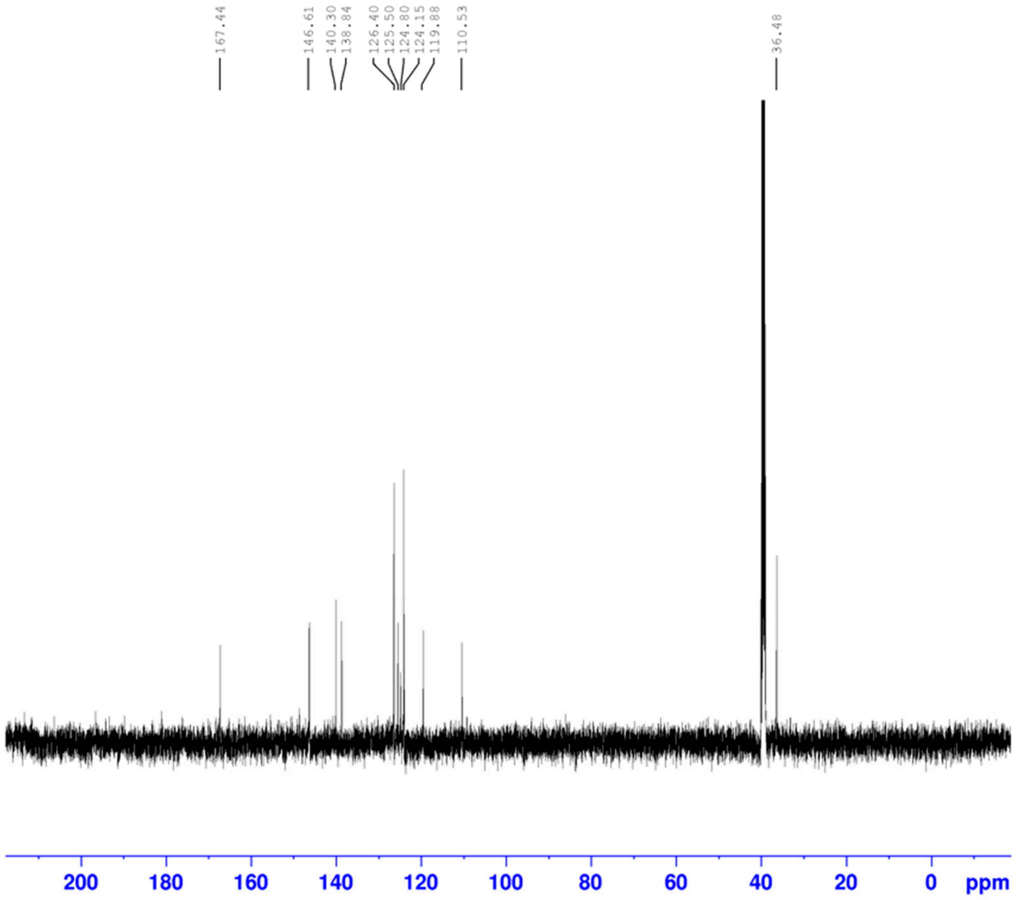

Figure S9. ^{13}C NMR Spectrum of compound **2**

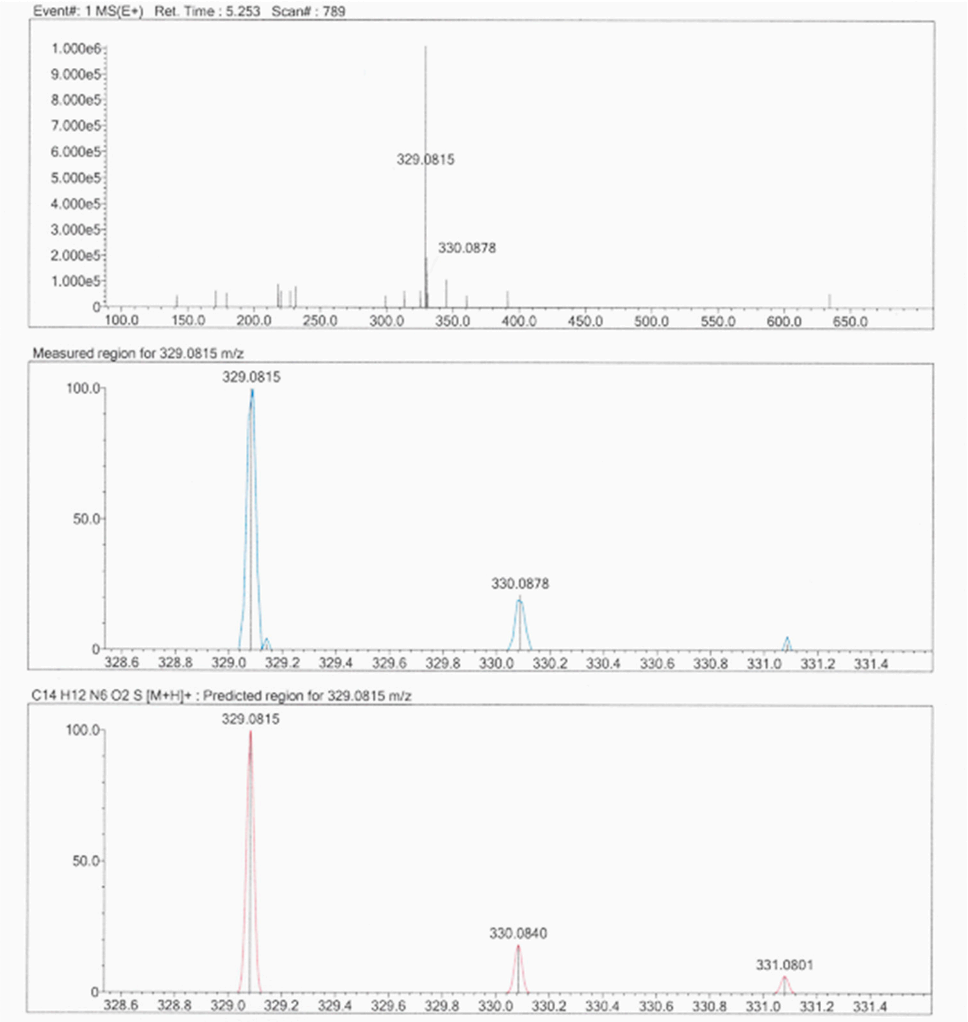

Rank	Score	Formula (M)	Ion	Meas. m/z	Pred. m/z	Df. (mDa)	Df. (ppm)	Iso	DBE
1	72.25	C14 H12 N6 O2 S	[M+H]+	329.0815	329.0815	-0.0	0.00	72.25	12.0

Figure S10. Mass Spectrum of compound **2**

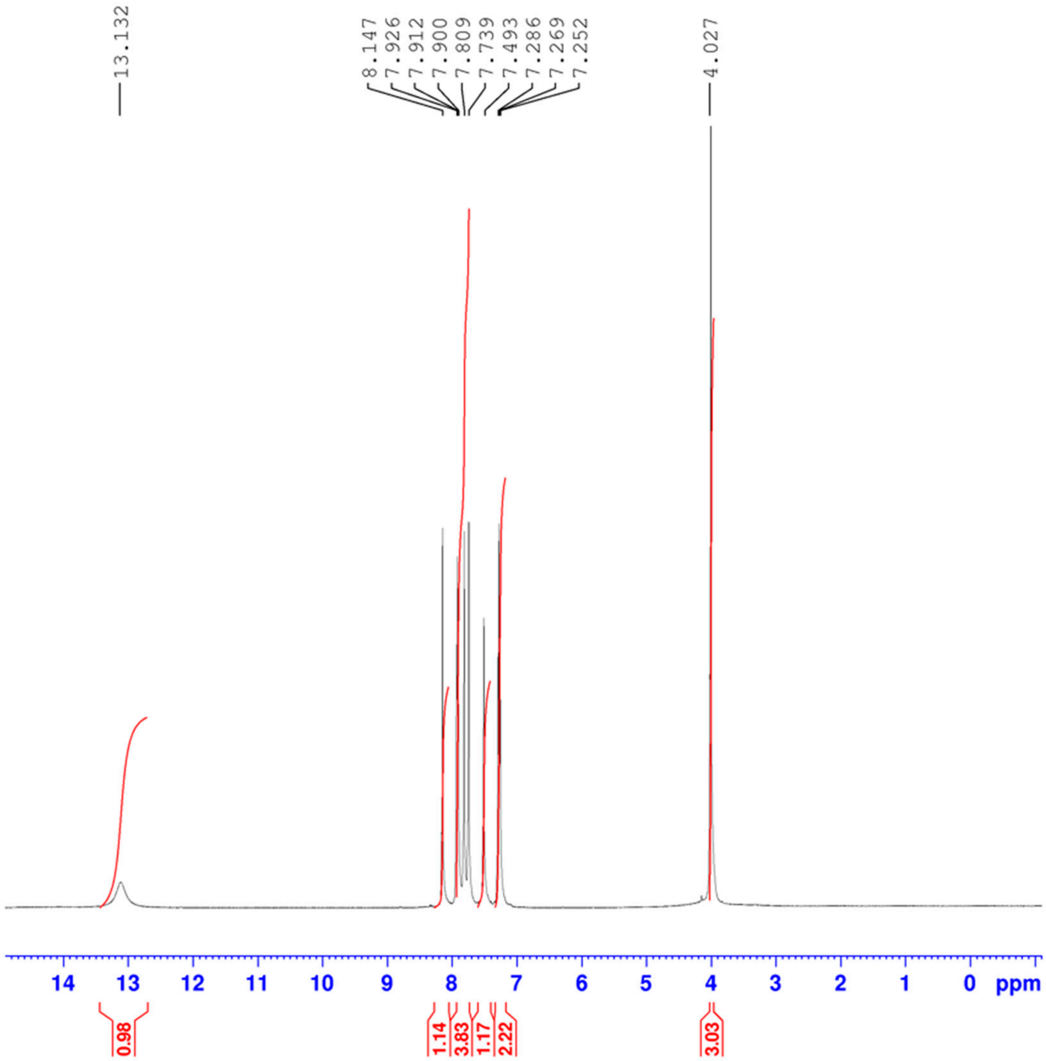

Figure S11. ^{1}H NMR Spectrum of compound **3** (0-15 ppm)

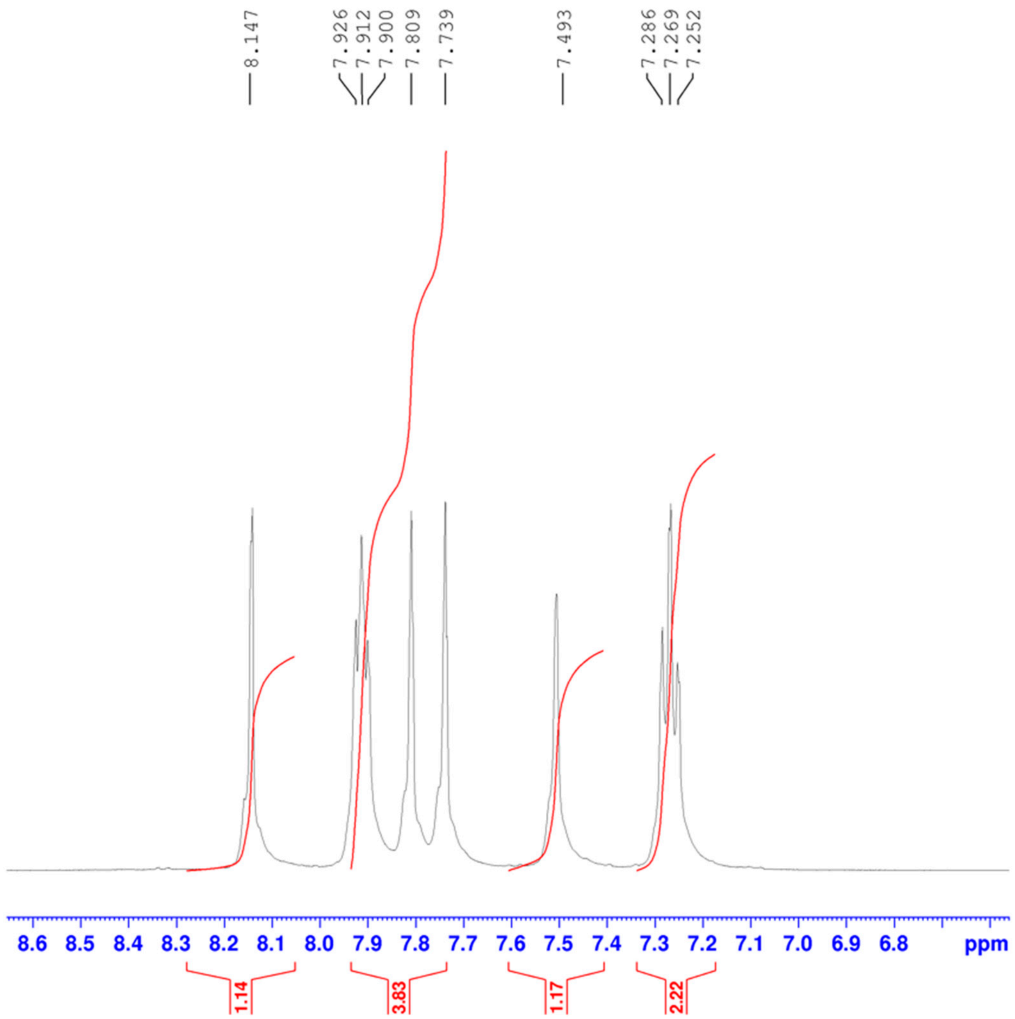

Figure S12. ^{1}H NMR Spectrum of compound **3** (7-9 ppm)

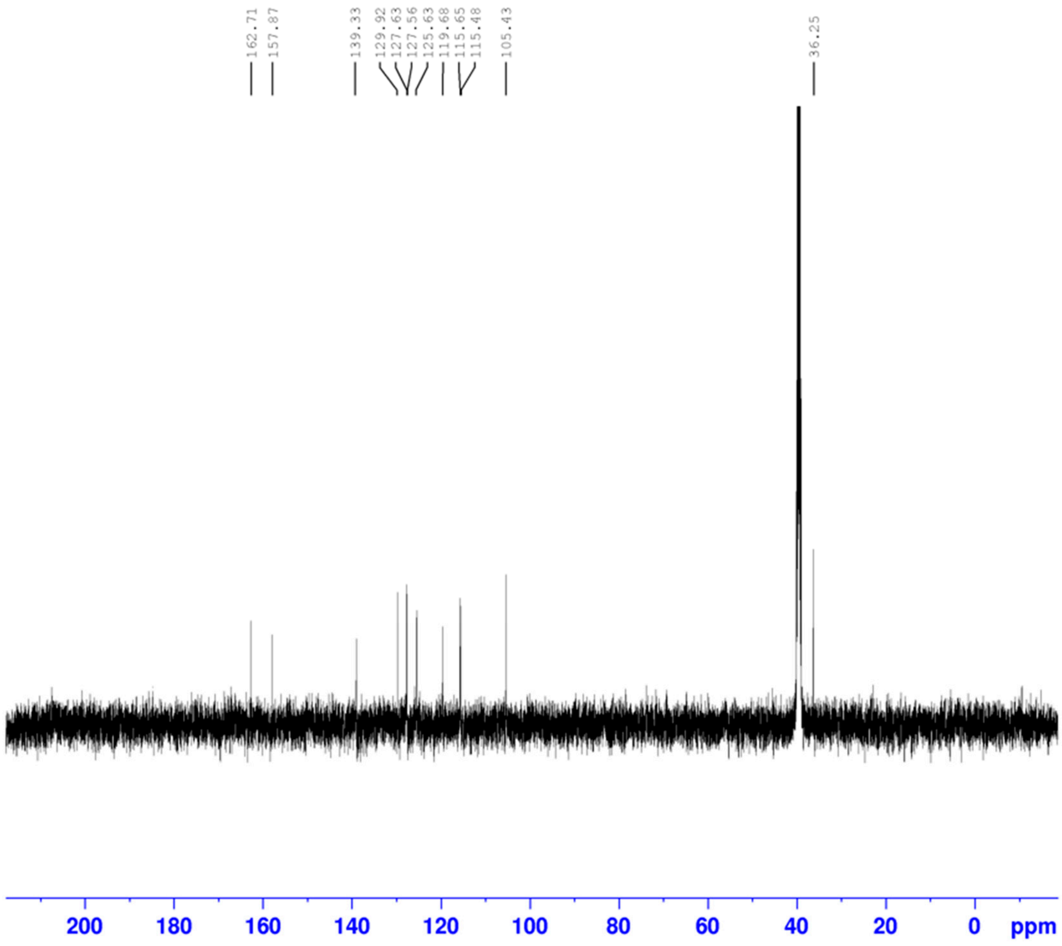

Figure S13.^{13}C NMR Spectrum of compound **3**

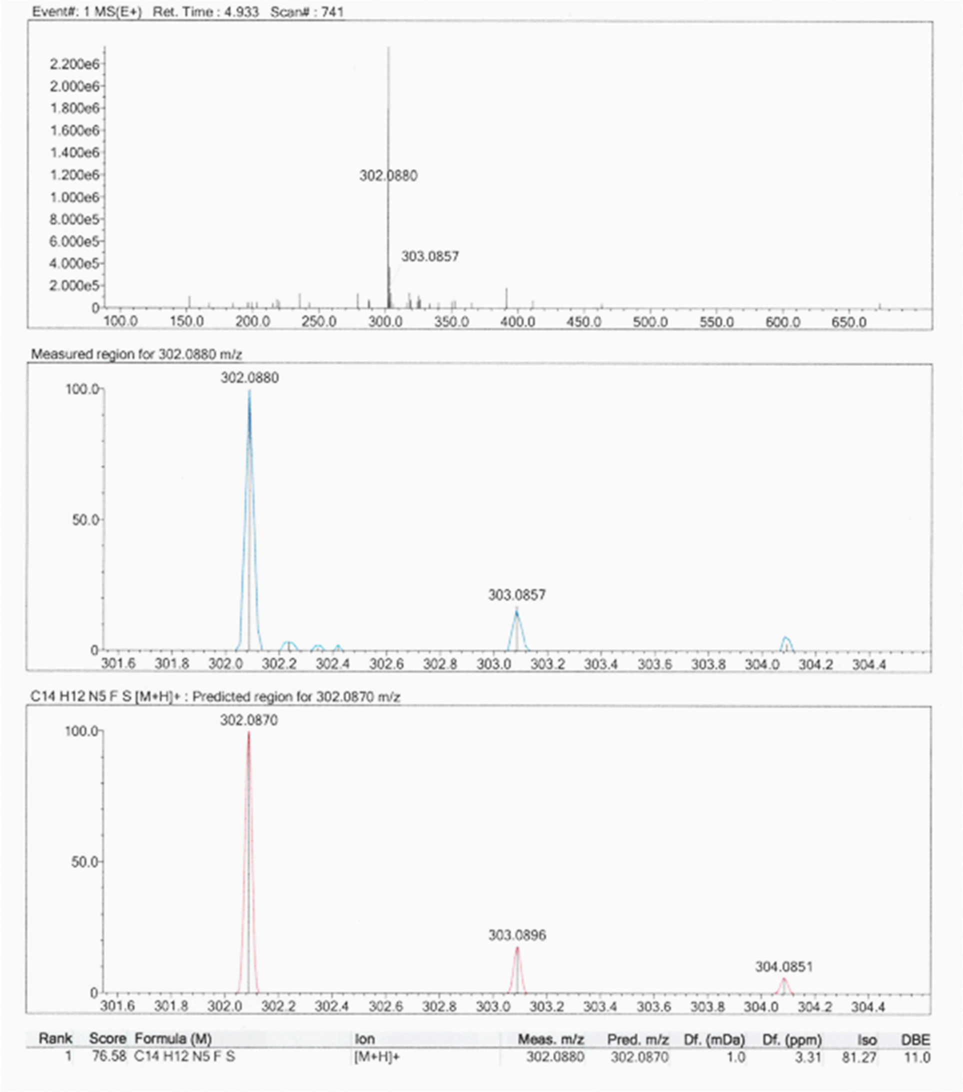

Figure S14. Mass Spectrum of compound **3**

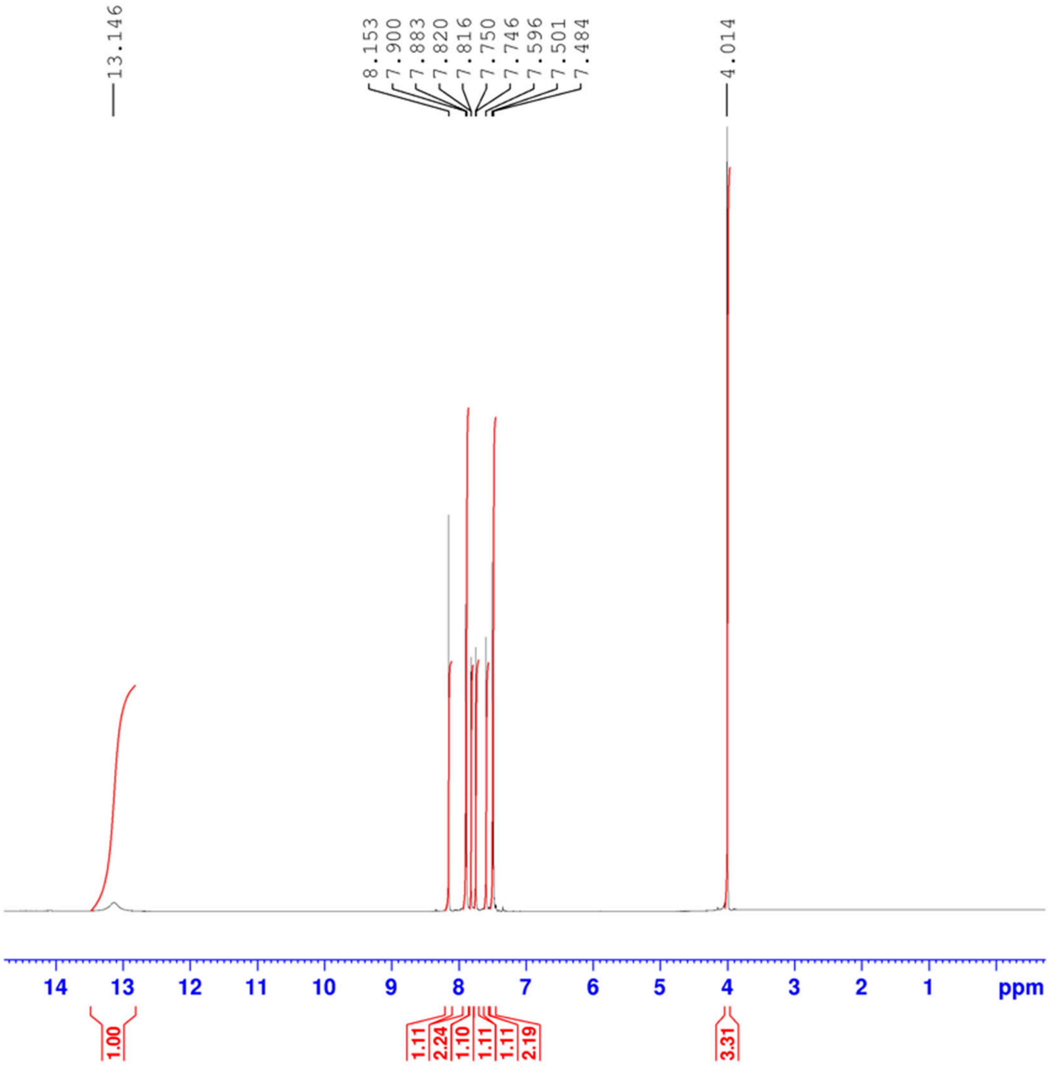

Figure S15. ^{1}H NMR Spectrum of compound **4** (0-15 ppm)

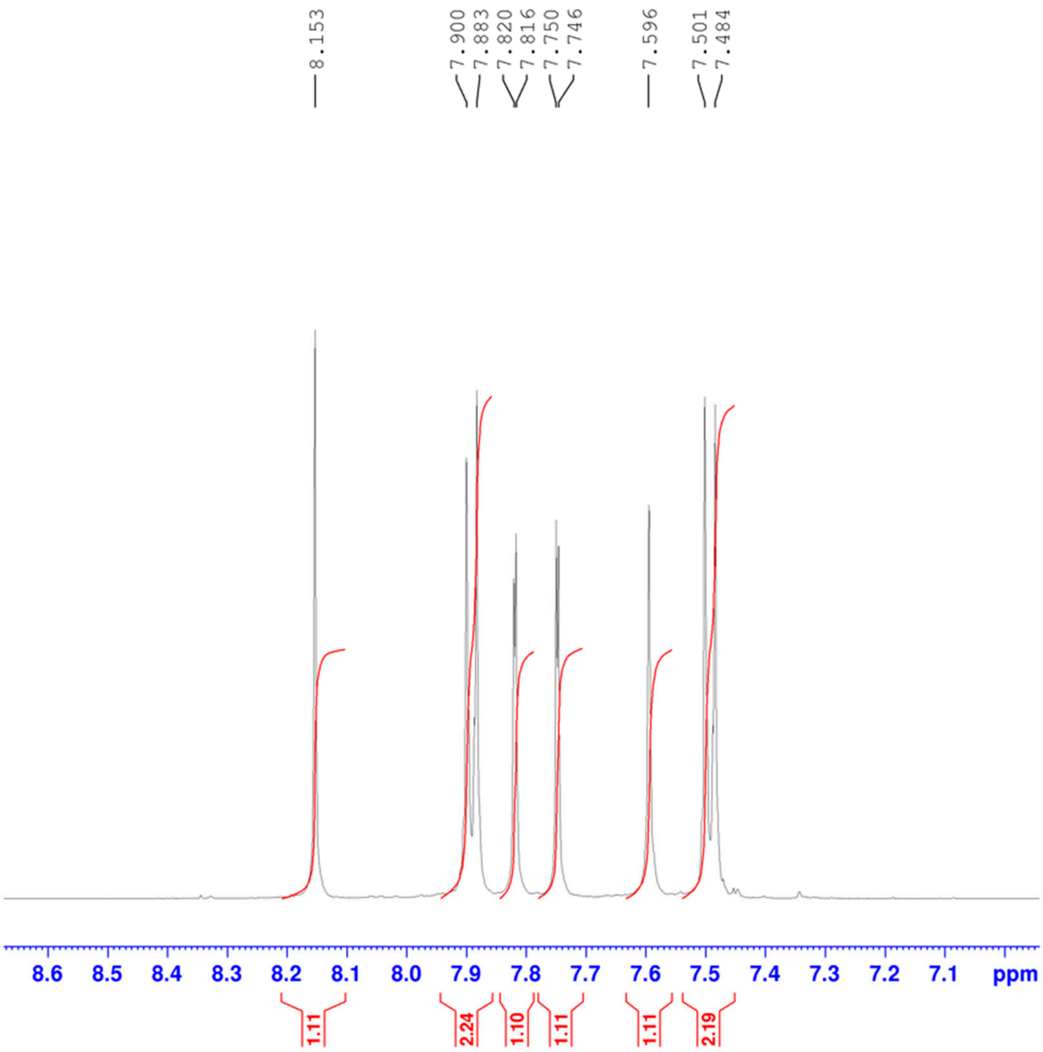

Figure S16. ^{1}H NMR Spectrum of compound **4** (7-9 ppm)

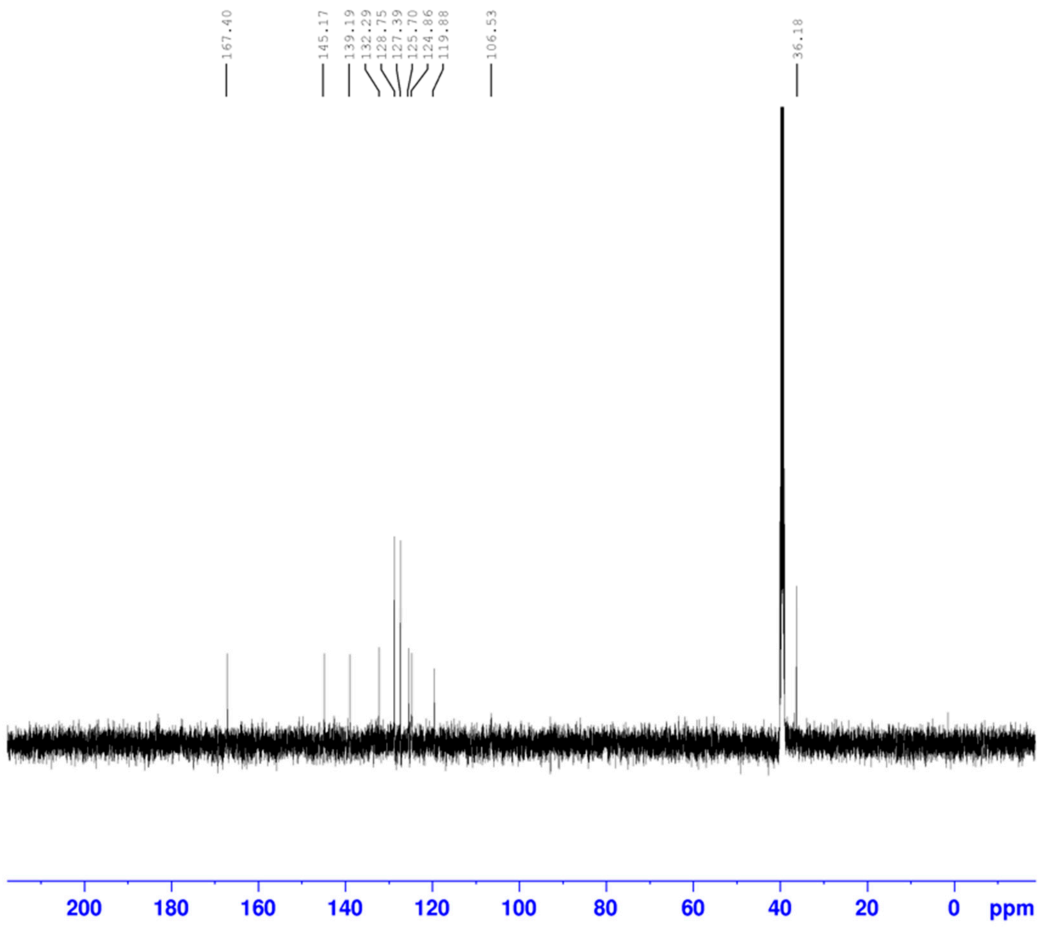

Figure S17. ^{13}C NMR Spectrum of compound **4**

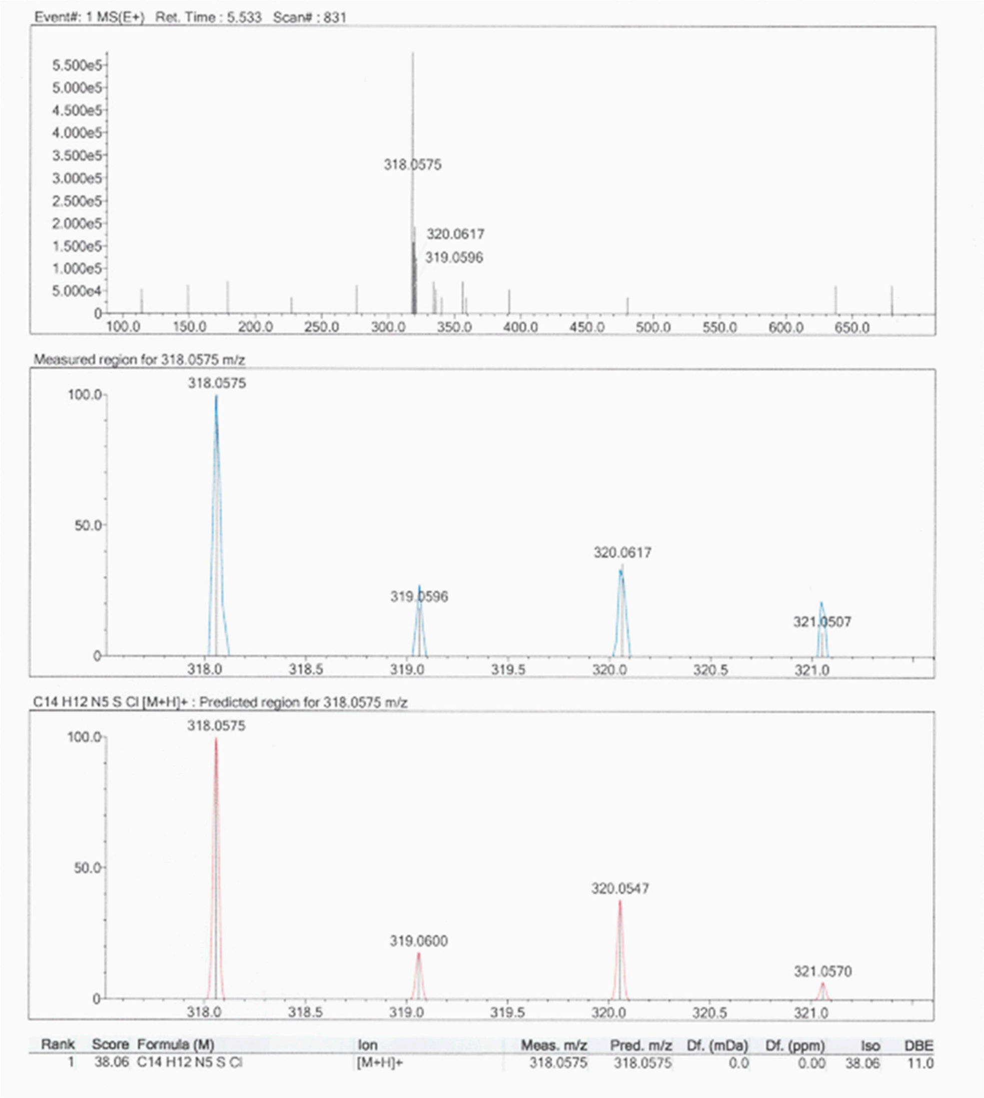

Figure S18. Mass Spectrum of compound **4**

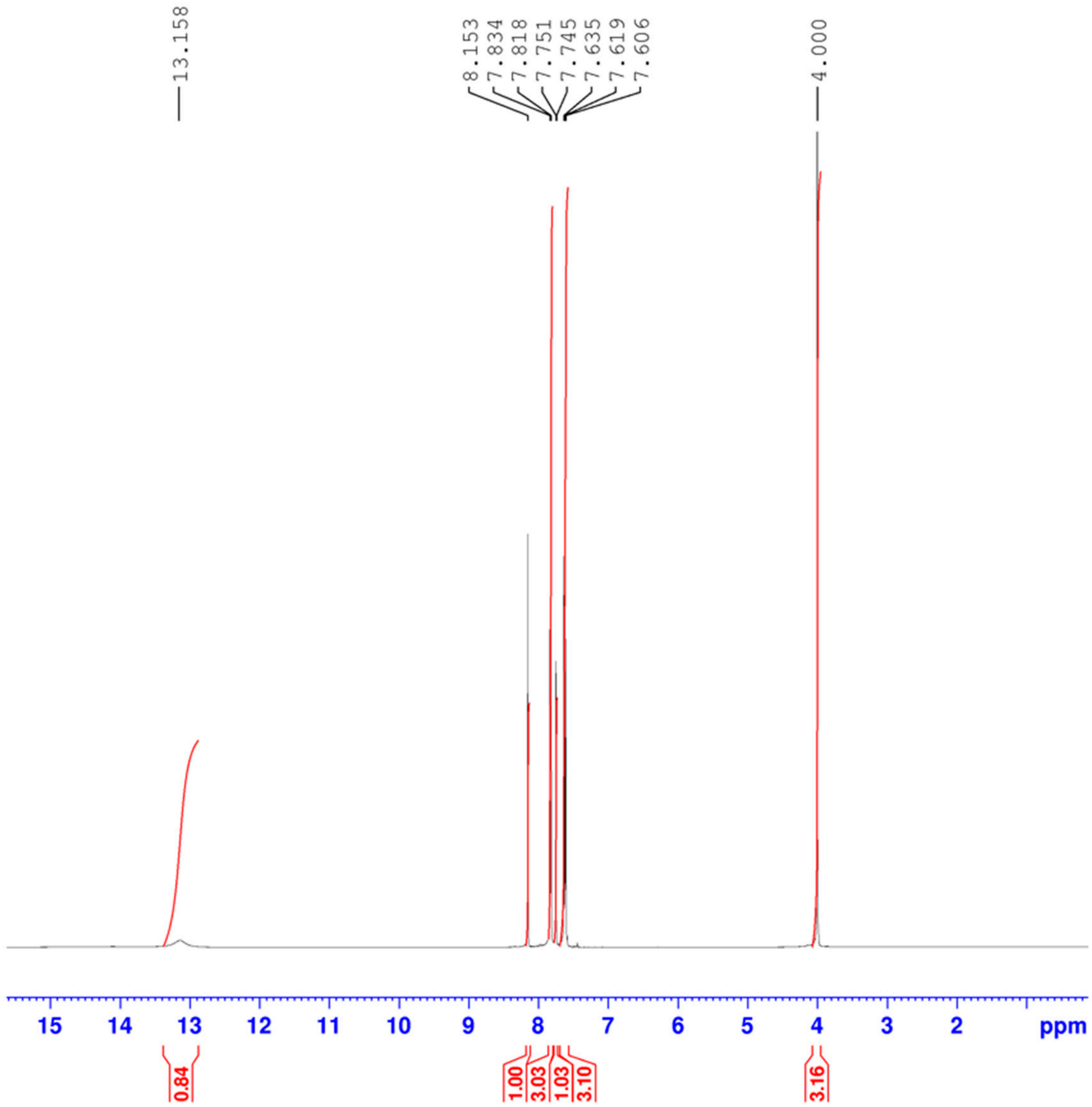

Figure S19. ^{1}H NMR Spectrum of compound **5** (0-15 ppm)

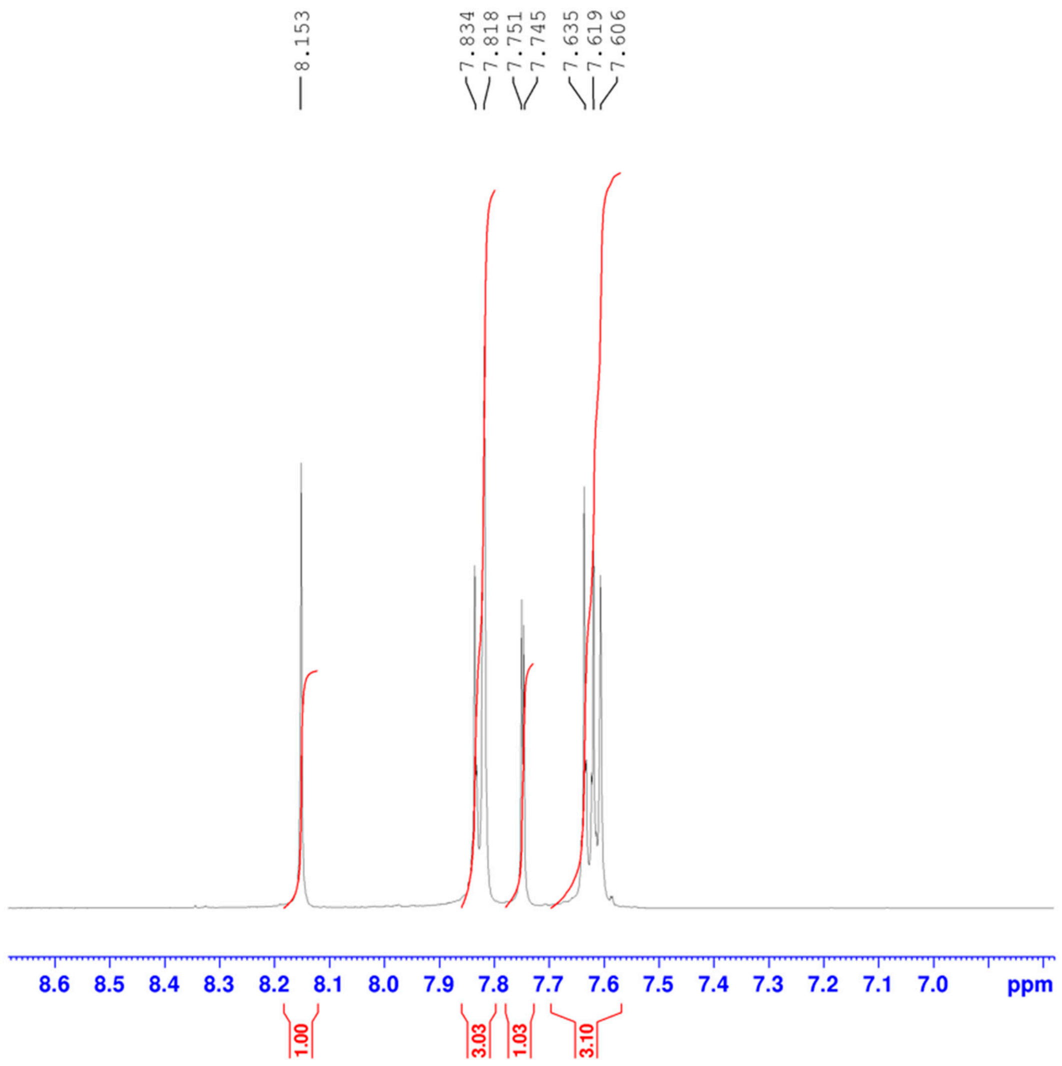

Figure S20. ^{1}H NMR Spectrum of compound **5** (7-9 ppm)

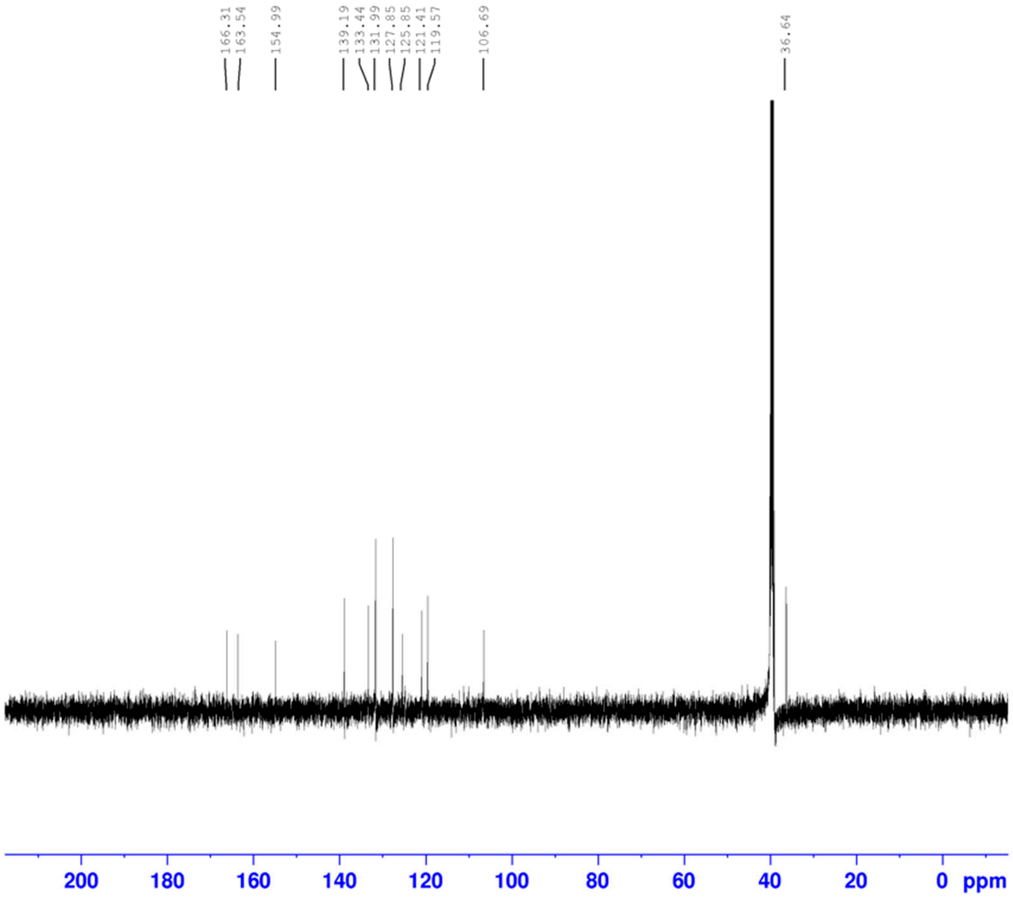

Figure S21. ^{13}C NMR Spectrum of compound **5**

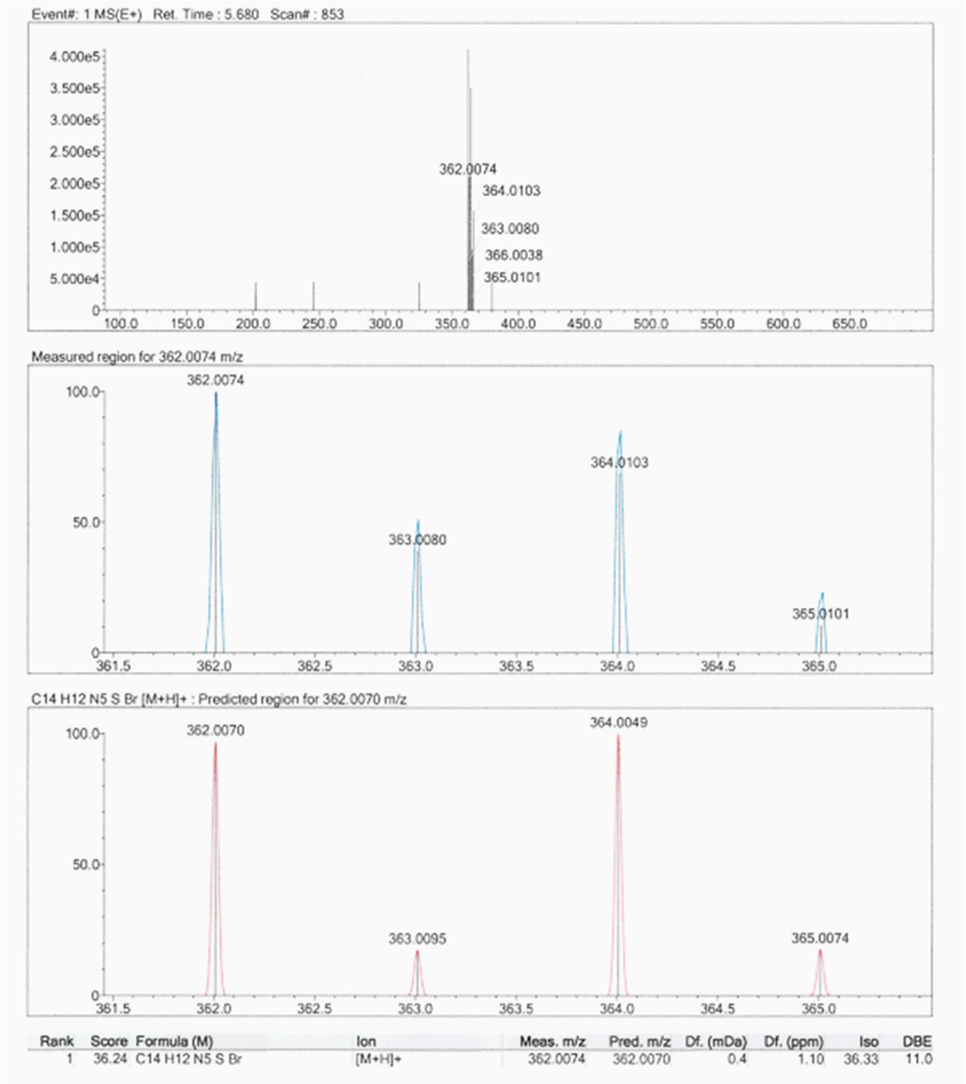

Figure S22. Mass Spectrum of compound **5**

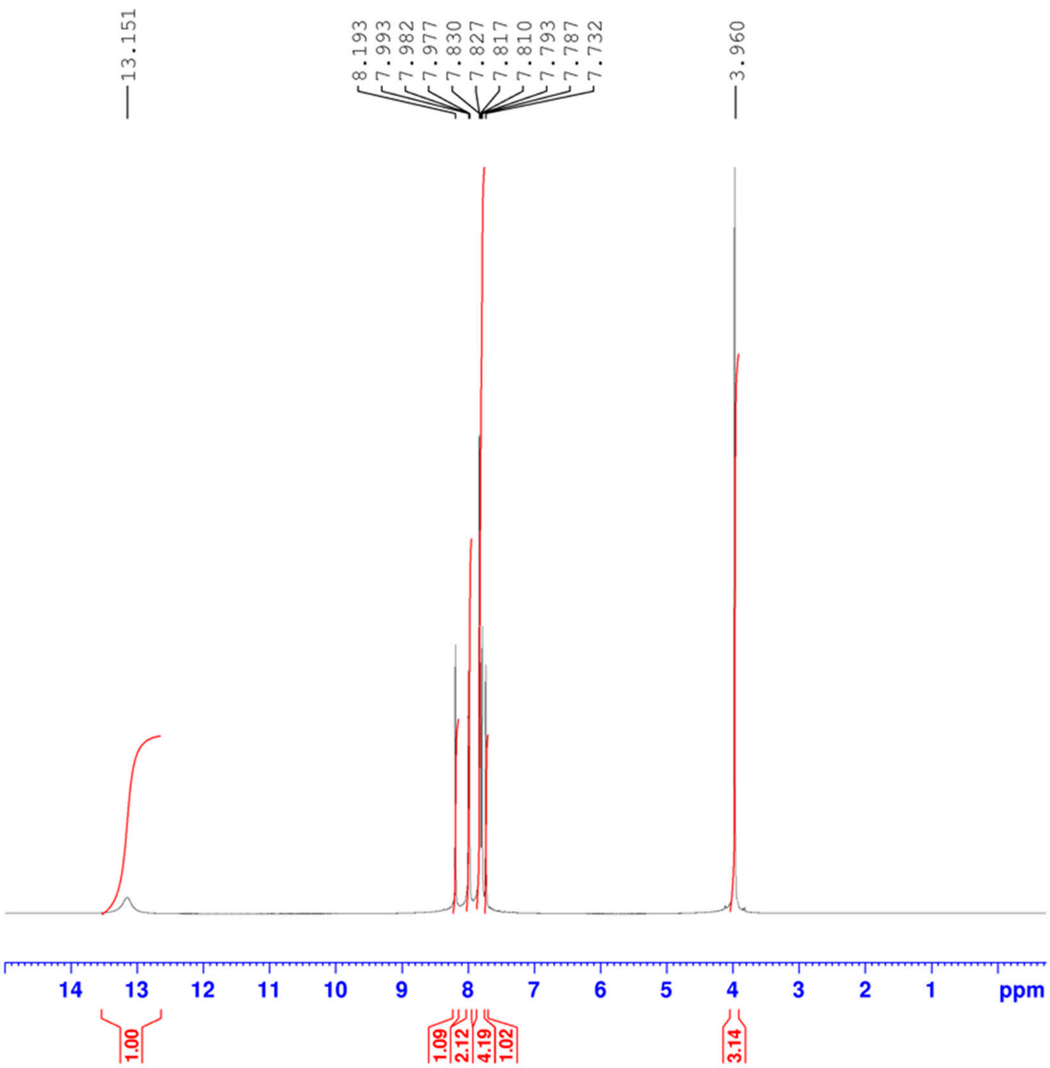

Figure S23. ^{1}H NMR Spectrum of compound **6** (0-15 ppm)

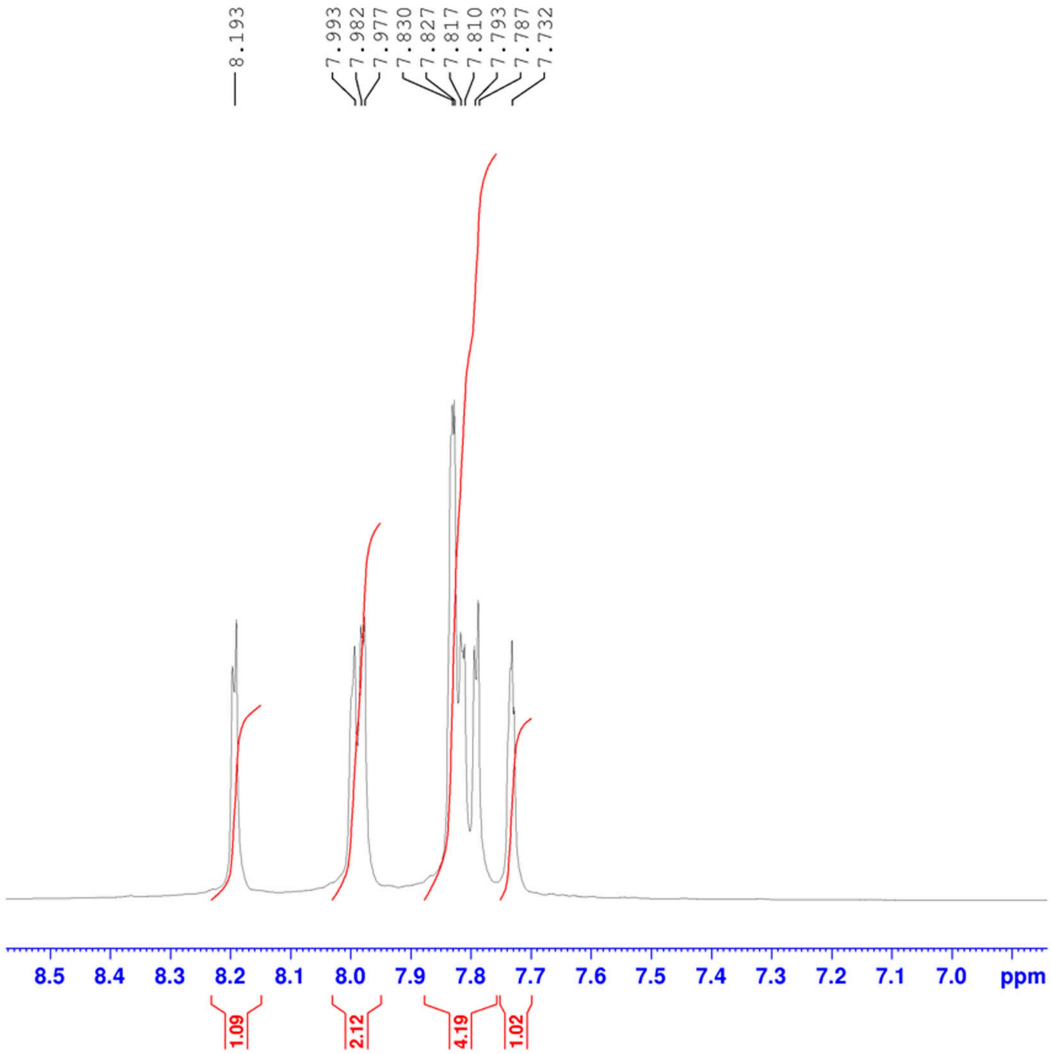

Figure S24. ^{1}H NMR Spectrum of compound **6** (7-9 ppm)

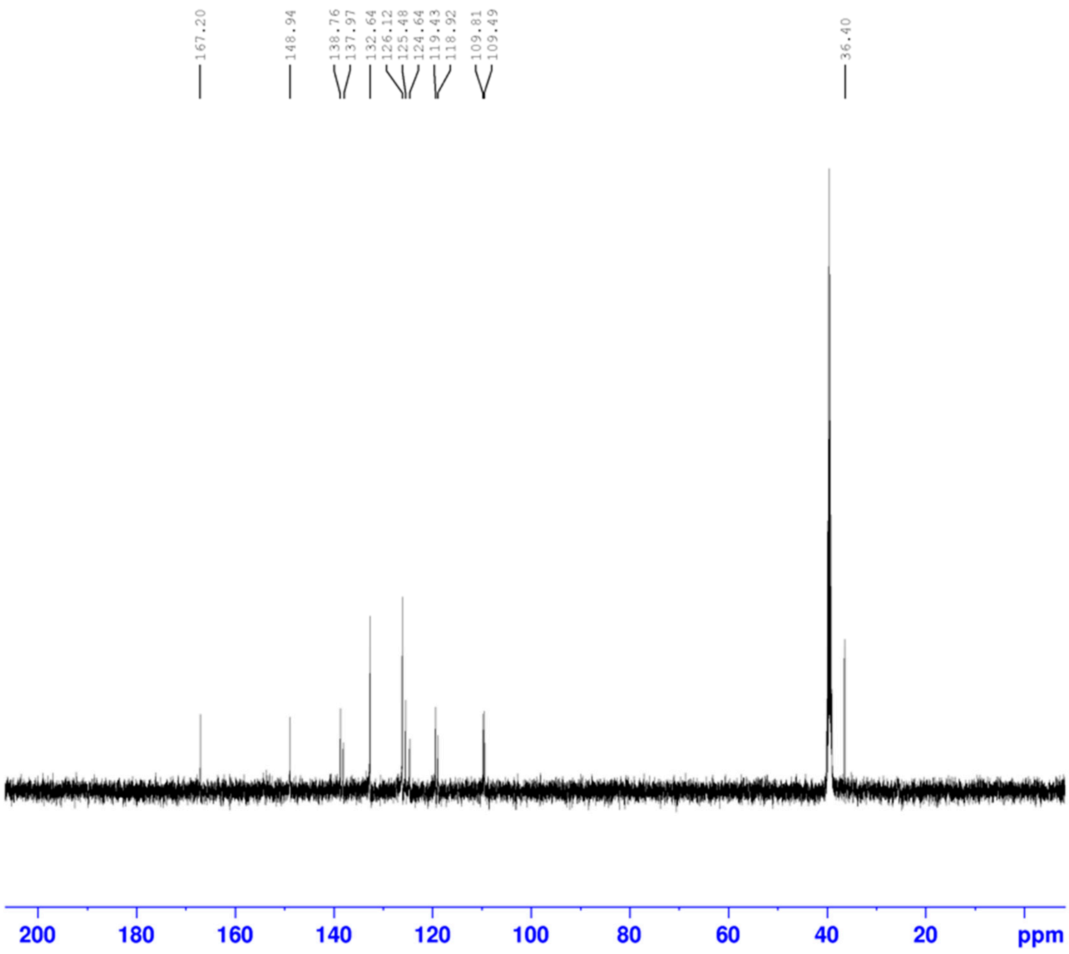

Figure S25. ^{13}C NMR Spectrum of compound **6**

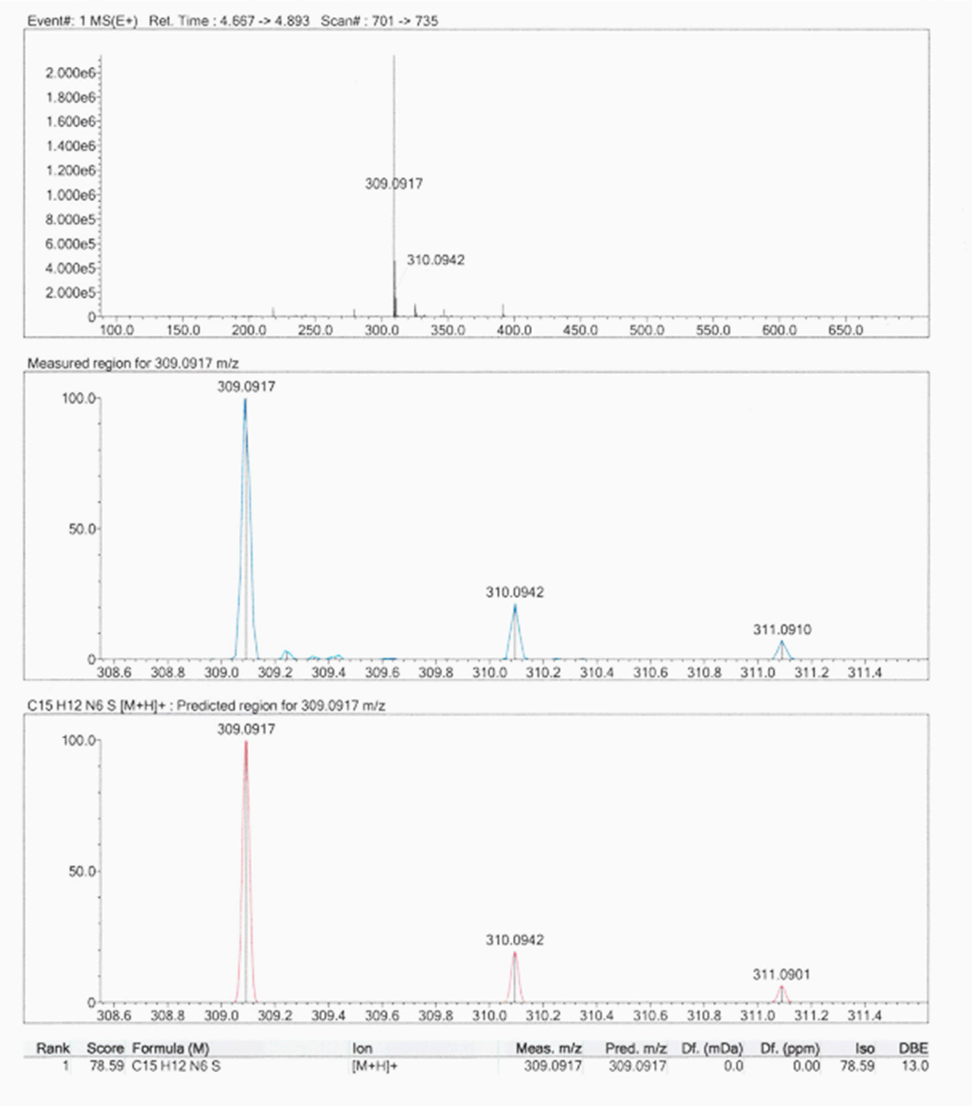

Figure S26. Mass Spectrum of compound **6**

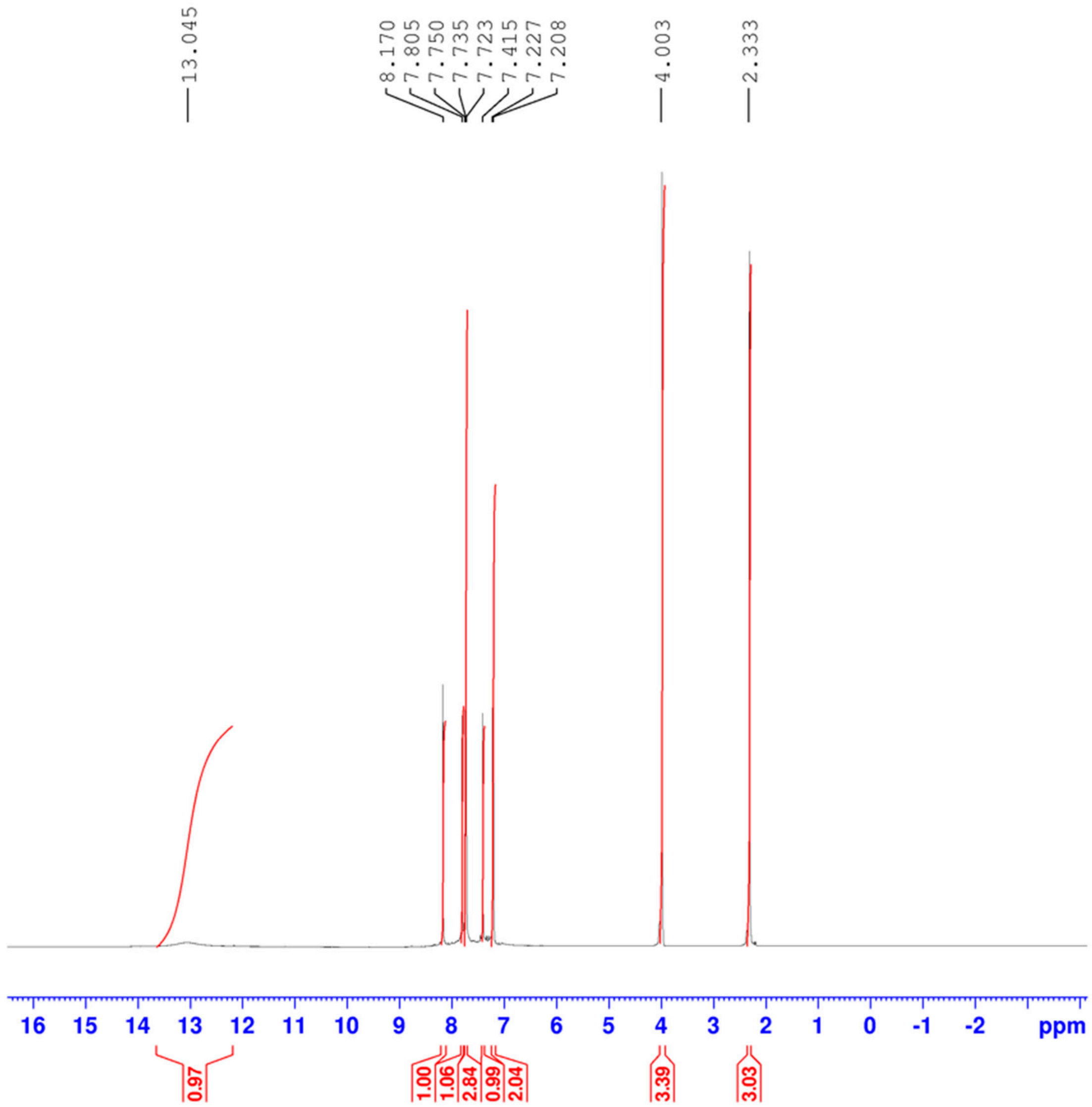

Figure S27. ^{1}H NMR Spectrum of compound **7** (0-15 ppm)

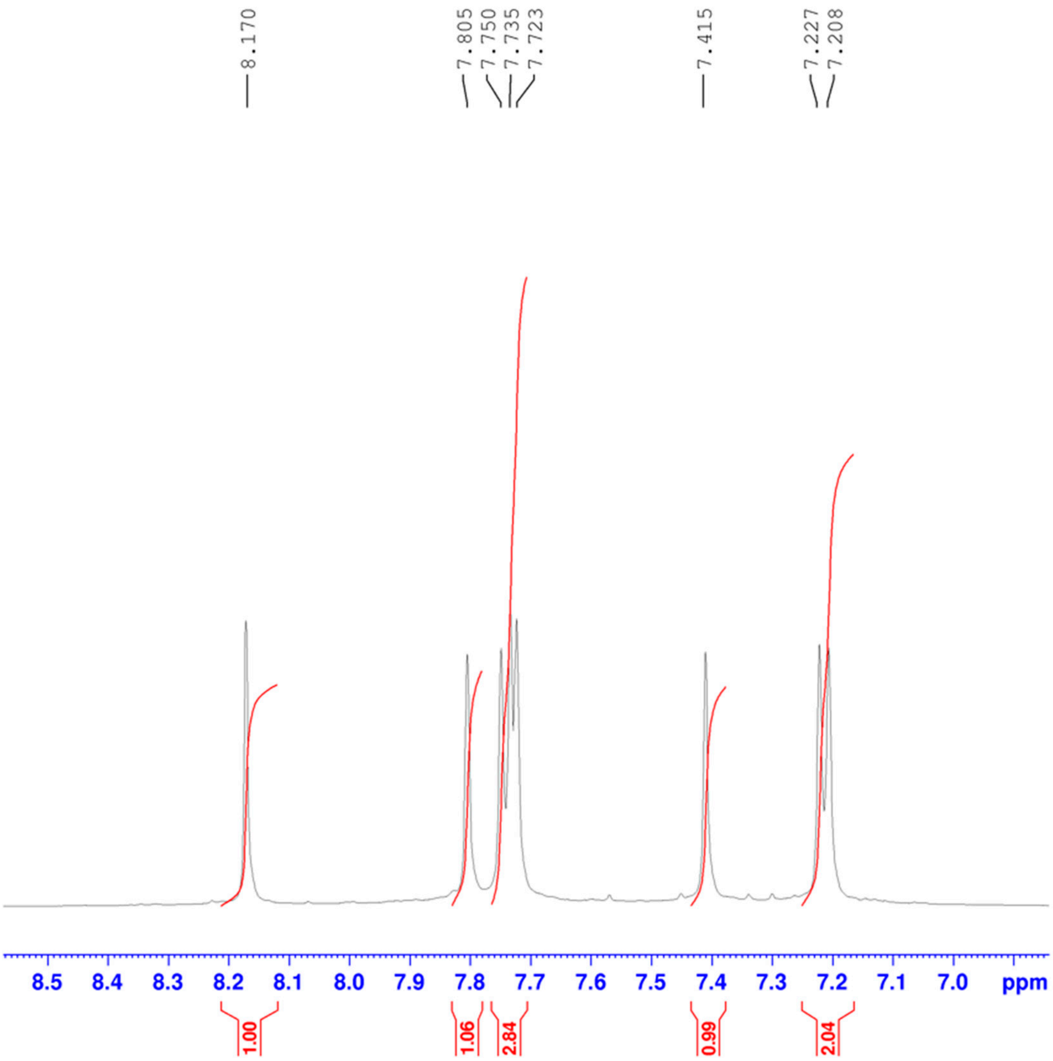

Figure S28. ^{1}H NMR Spectrum of compound **7** (7-9 ppm)

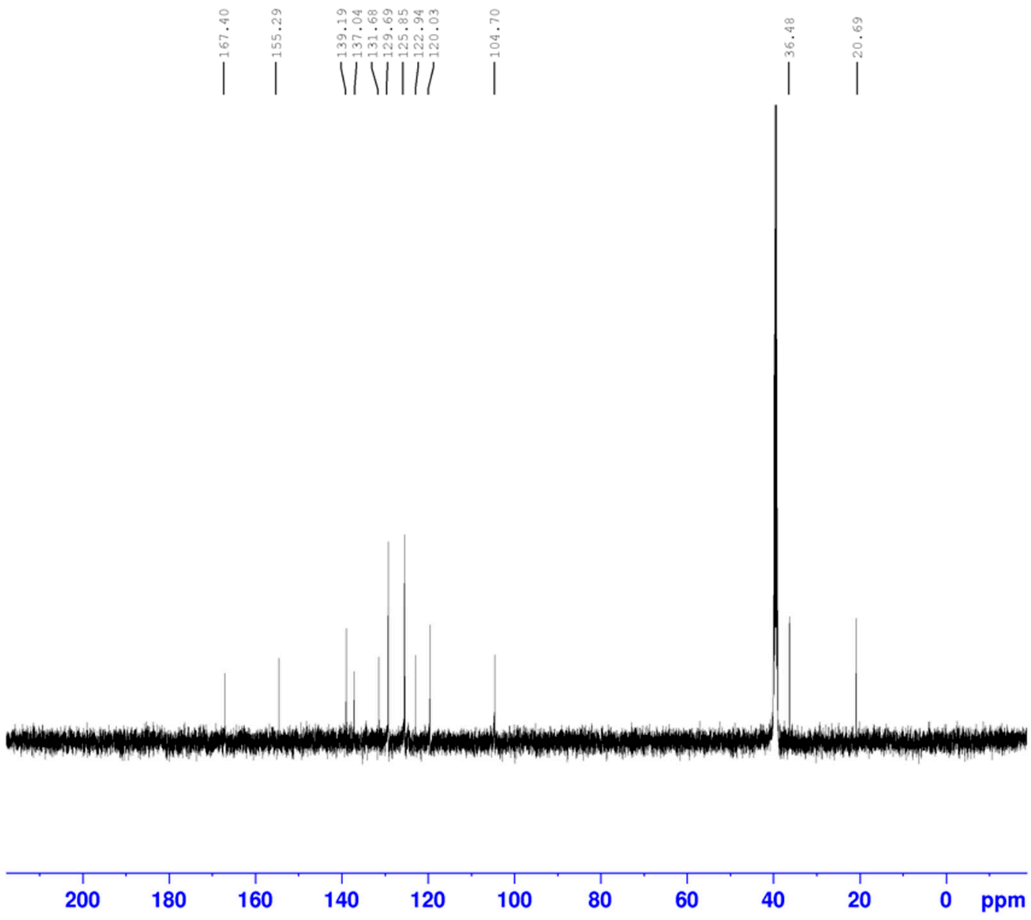

Figure S29. ^{13}C NMR Spectrum of compound **7**

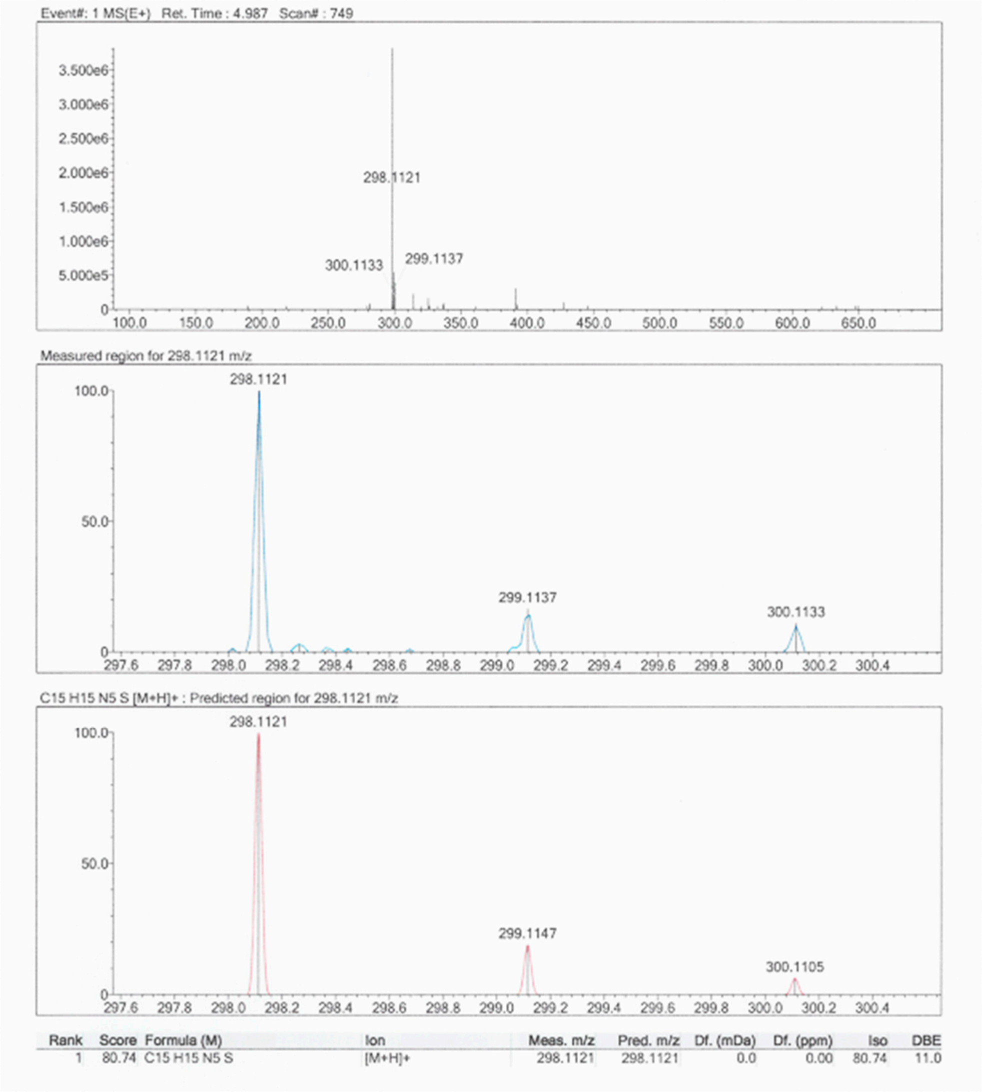

Figure S30. Mass Spectrum of compound **7**

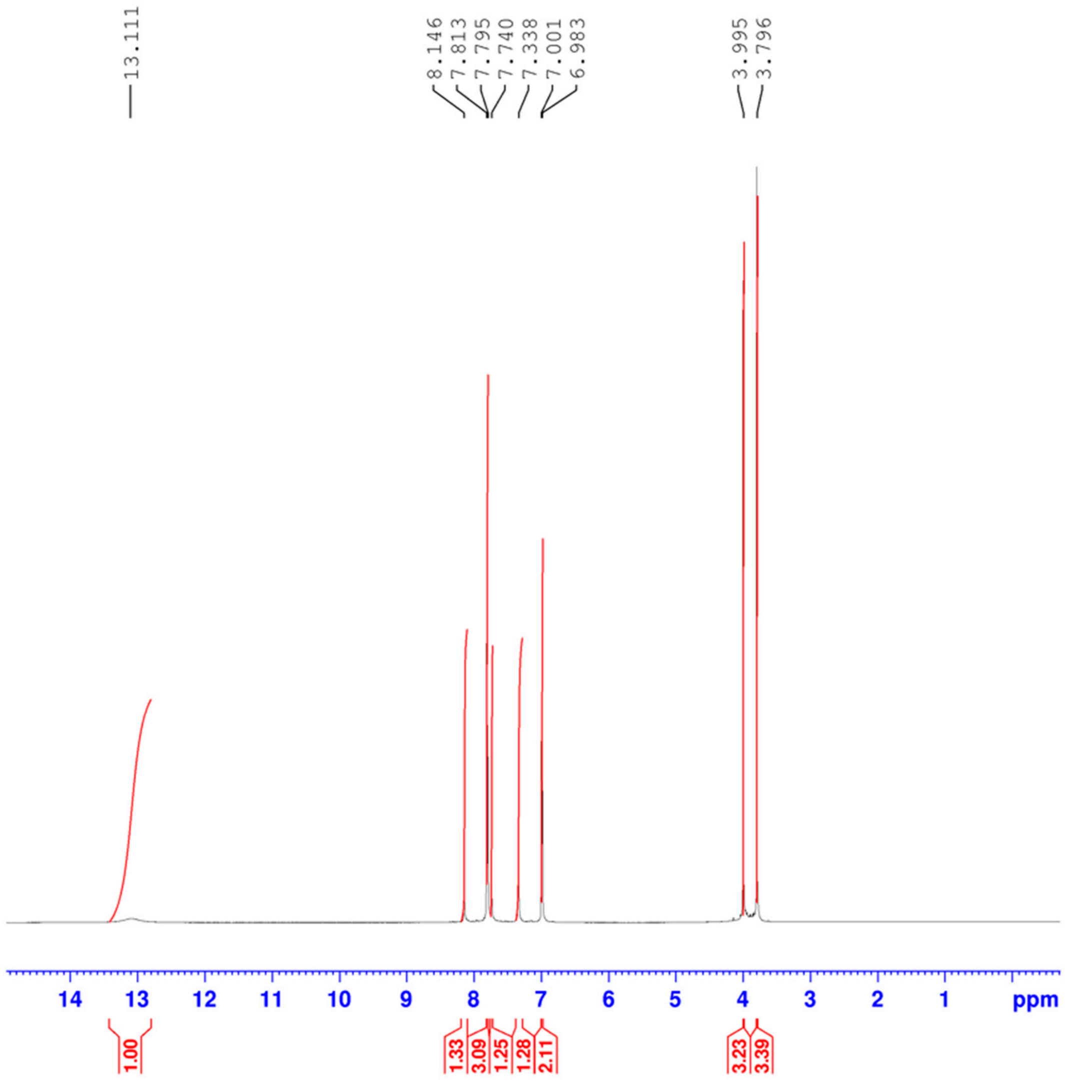

Figure S31. ^{1}H NMR Spectrum of compound **8** (0-15 ppm)

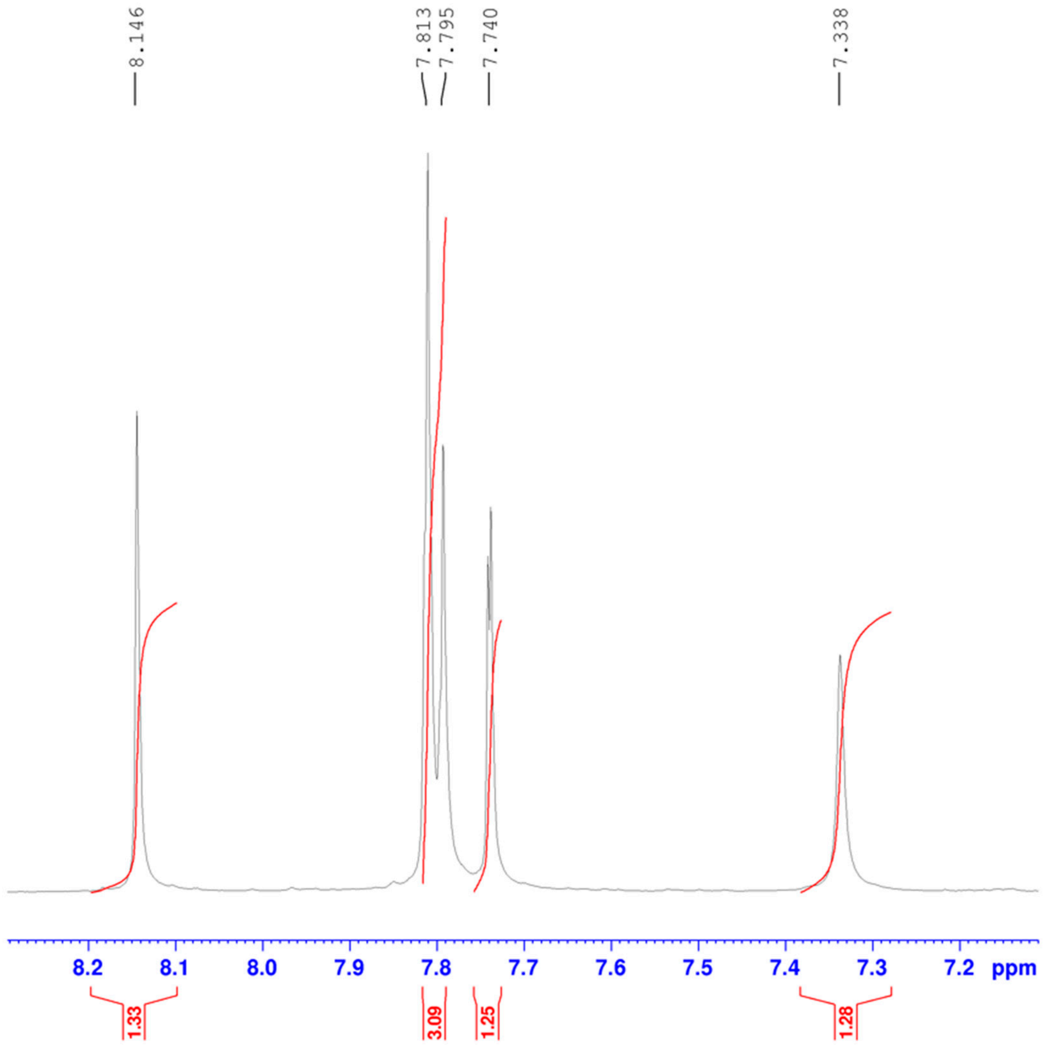

Figure S32. ^{1}H NMR Spectrum of compound **8** (7-9 ppm)

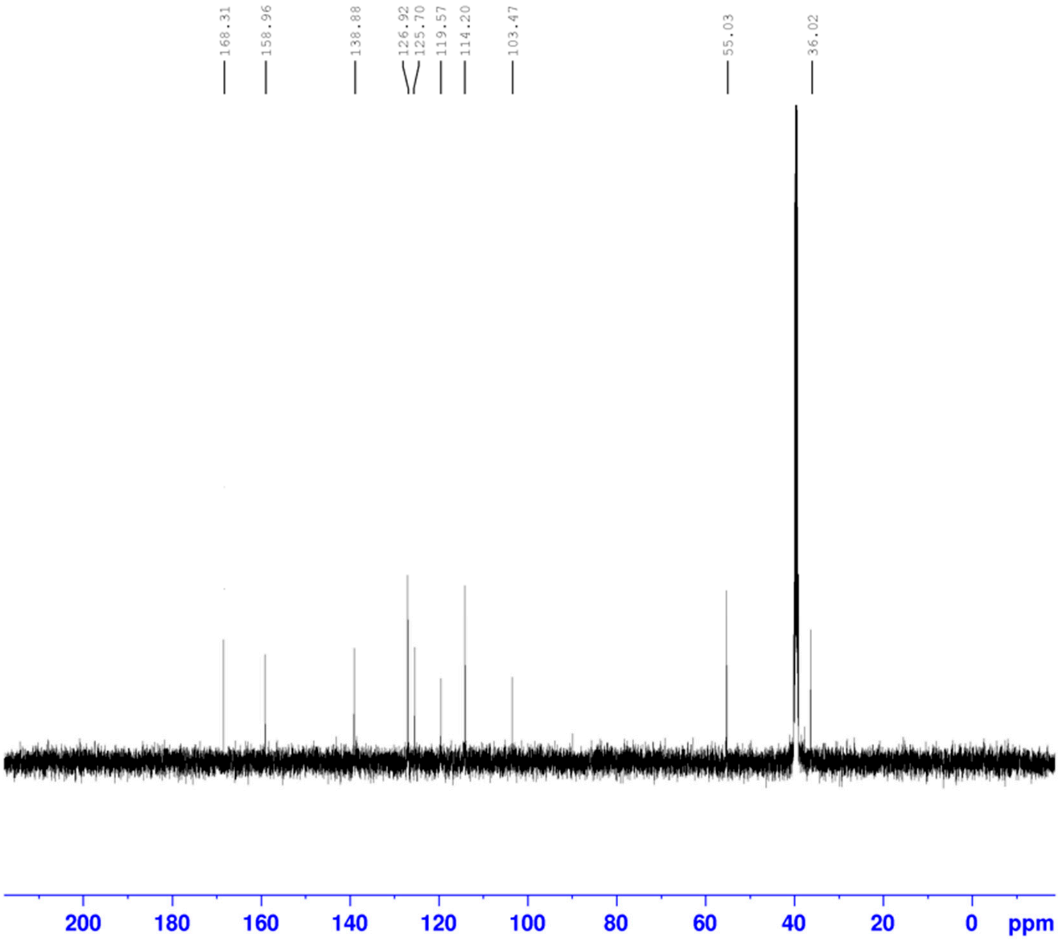

Figure S33. ^{13}C NMR Spectrum of compound **8**

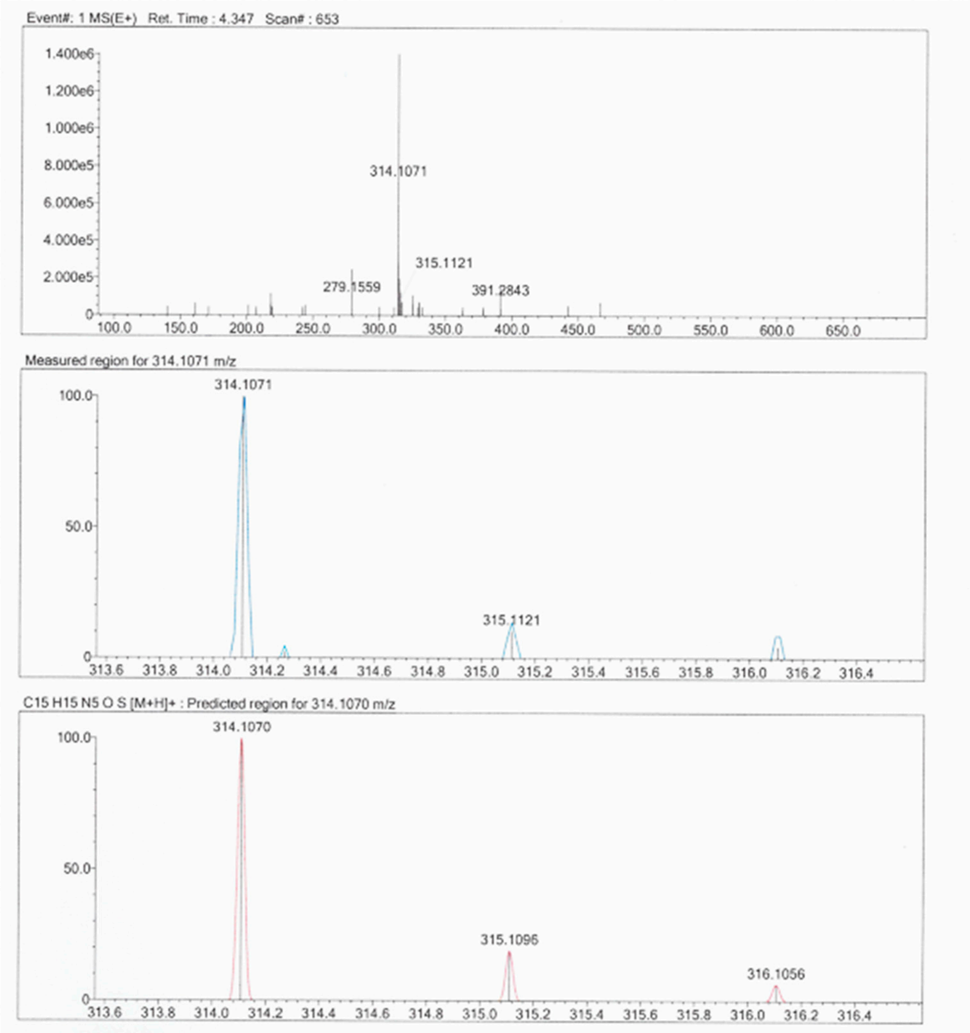

Rank	Score	Formula (M)	Ion	Meas. m/z	Pred. m/z	Df. (mDa)	Df. (ppm)	Iso	DBE
1	78.11	C15 H15 N5 O S	[M+H]+	314.1071	314.1070	0.1	0.32	78.11	11.0

Figure S34. Mass Spectrum of compound **8**

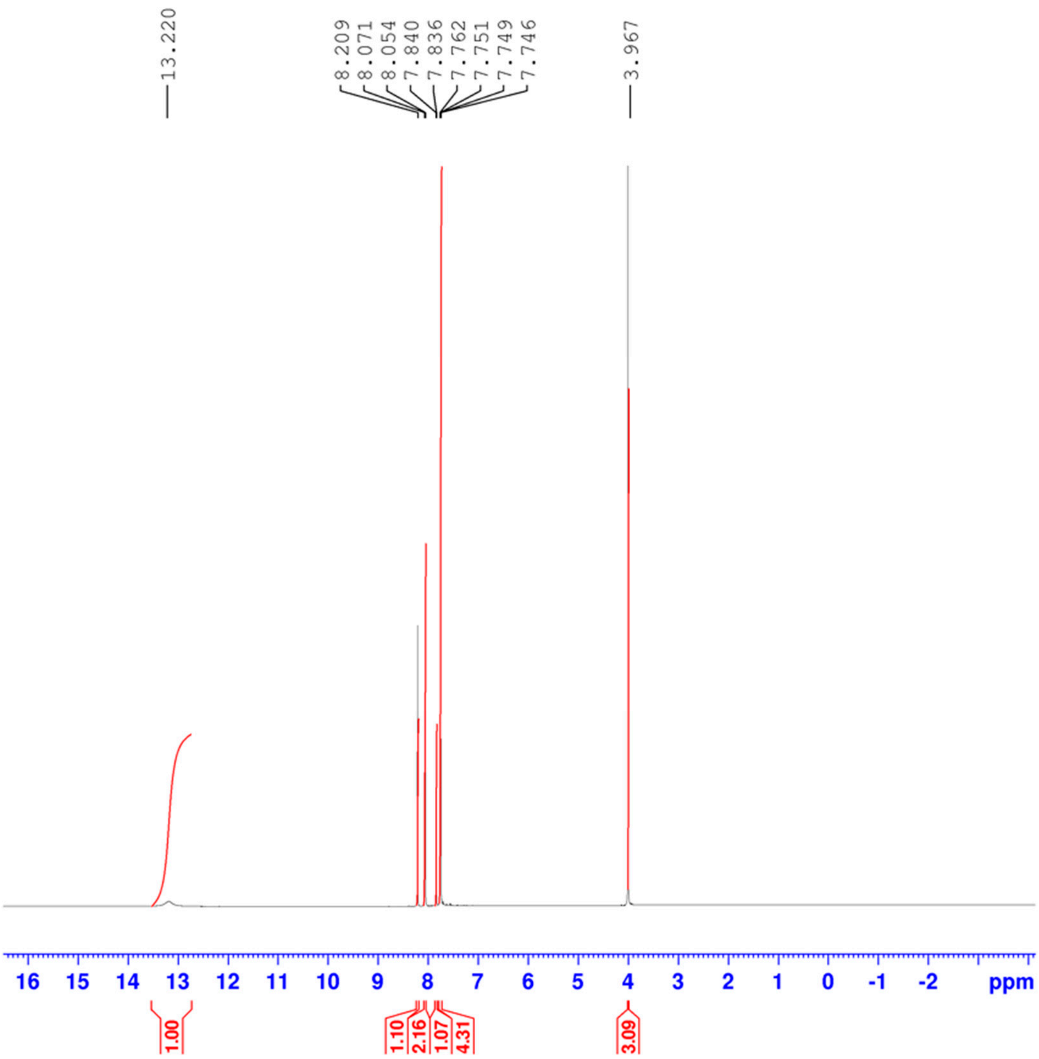

Figure S35. ^{1}H NMR Spectrum of compound **9** (0-15 ppm)

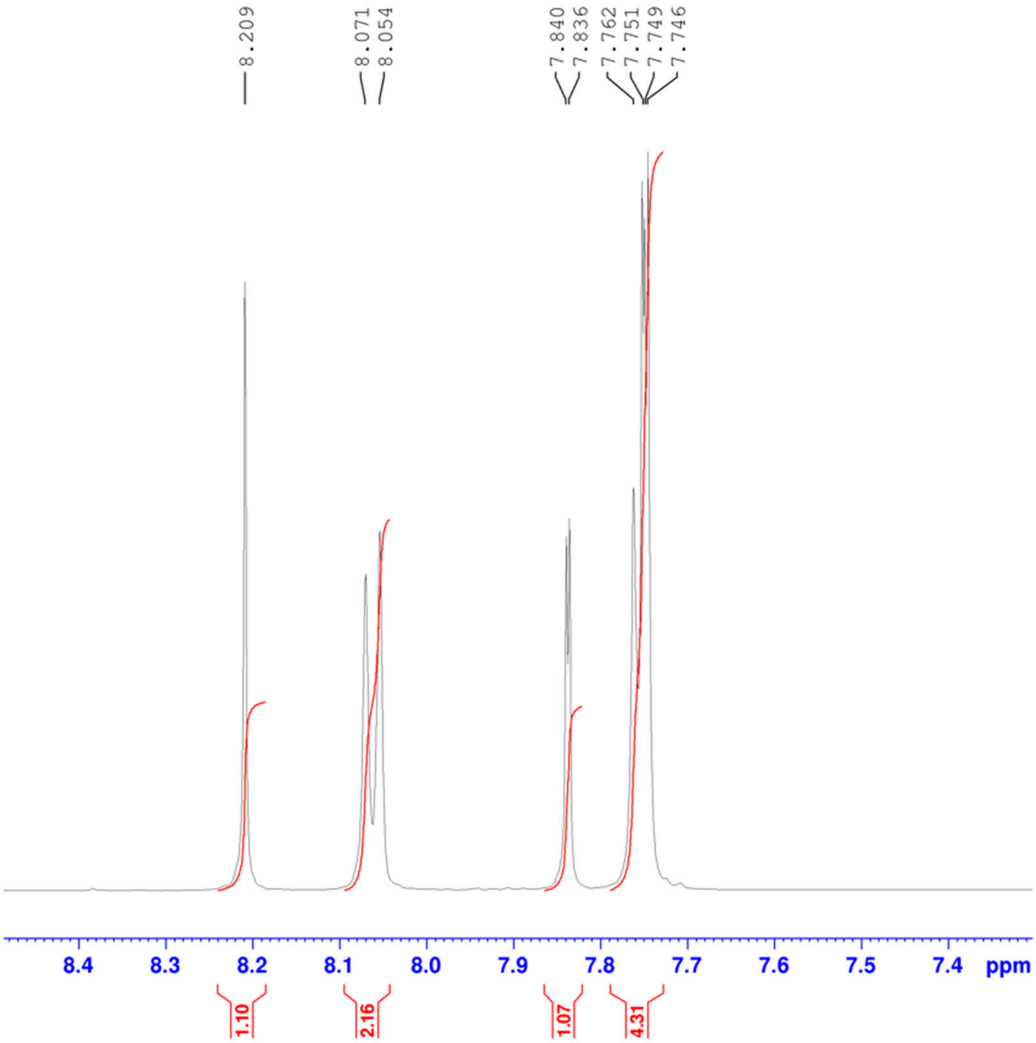

Figure S36. ^{1}H NMR Spectrum of compound **9** (7-9 ppm)

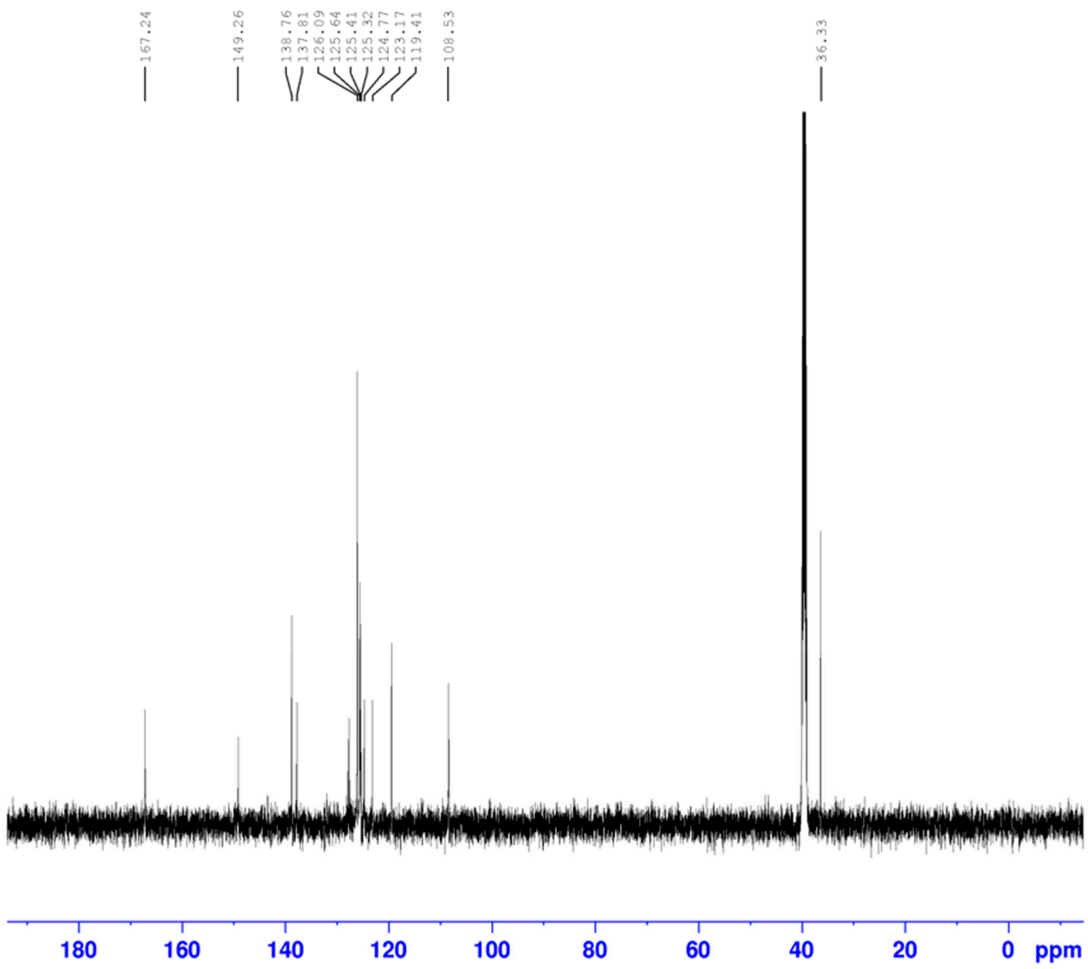

Figure S37. ^{13}C NMR Spectrum of compound **9**

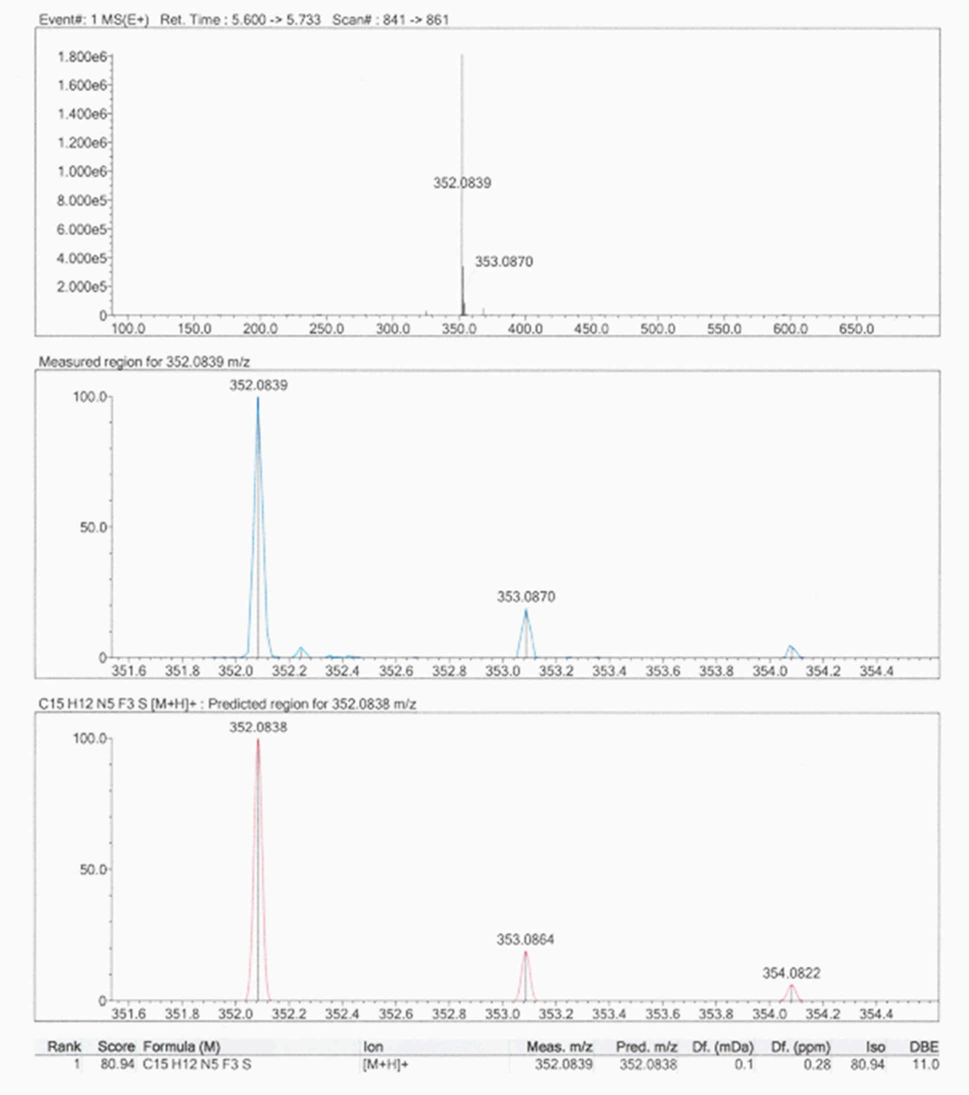

Figure S38. Mass Spectrum of compound **9**

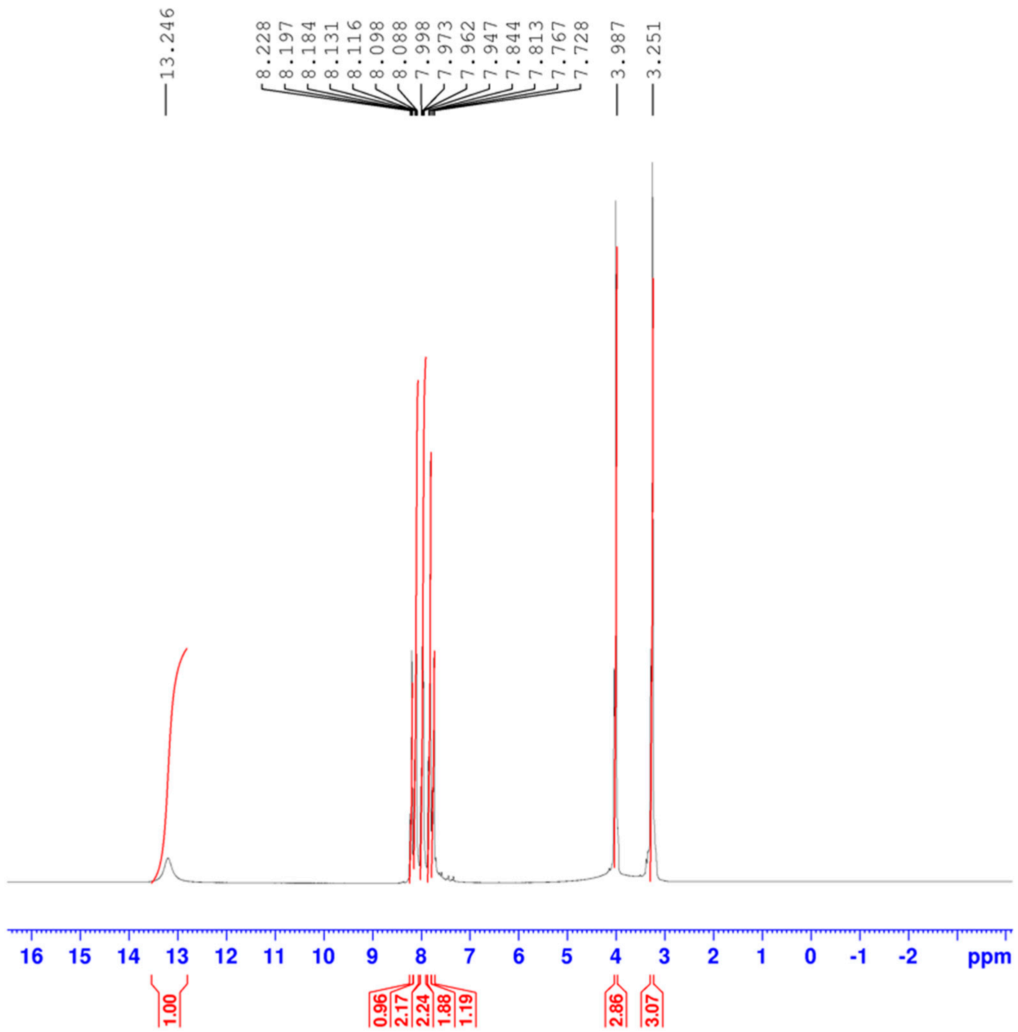

Figure S39: ^{1}H NMR Spectrum of compound **10** (0-15 ppm)

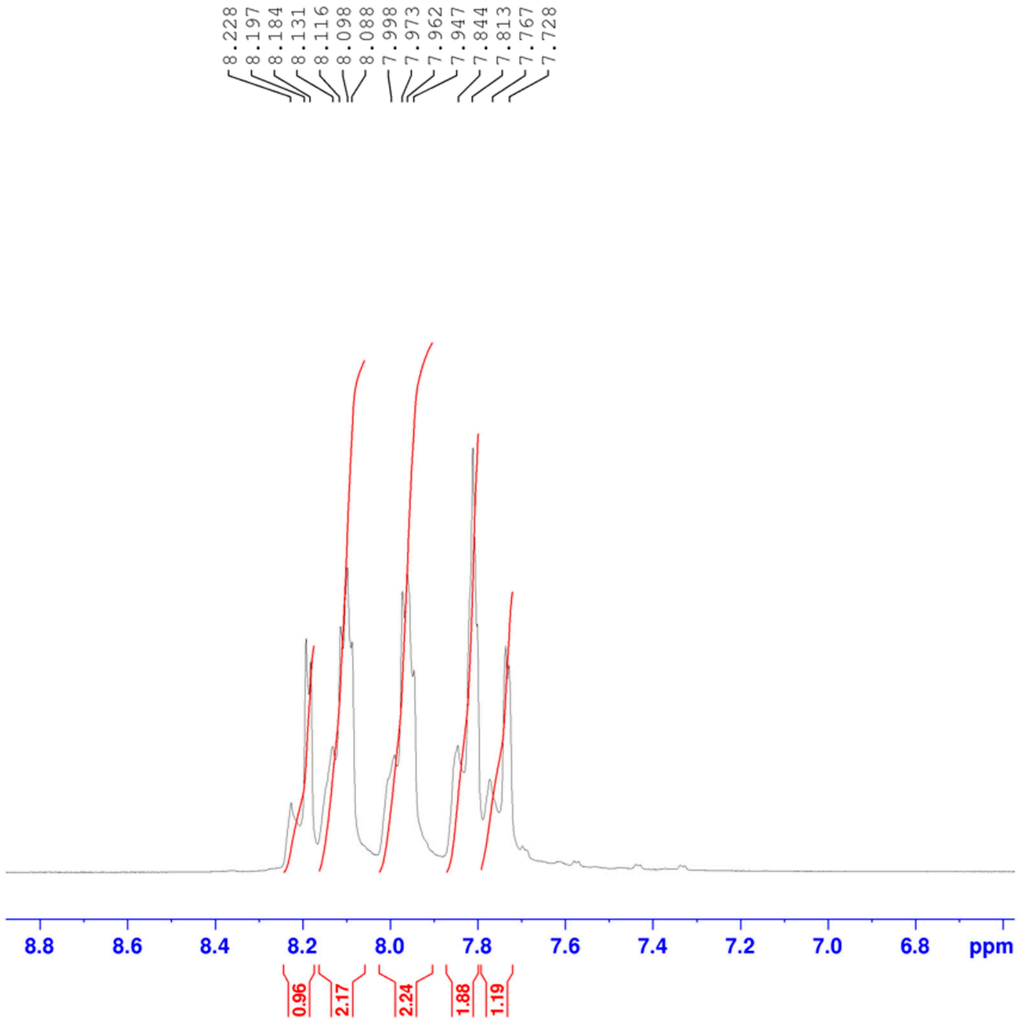

Figure S40: ^{1}H NMR Spectrum of compound **10** (7-9 ppm)

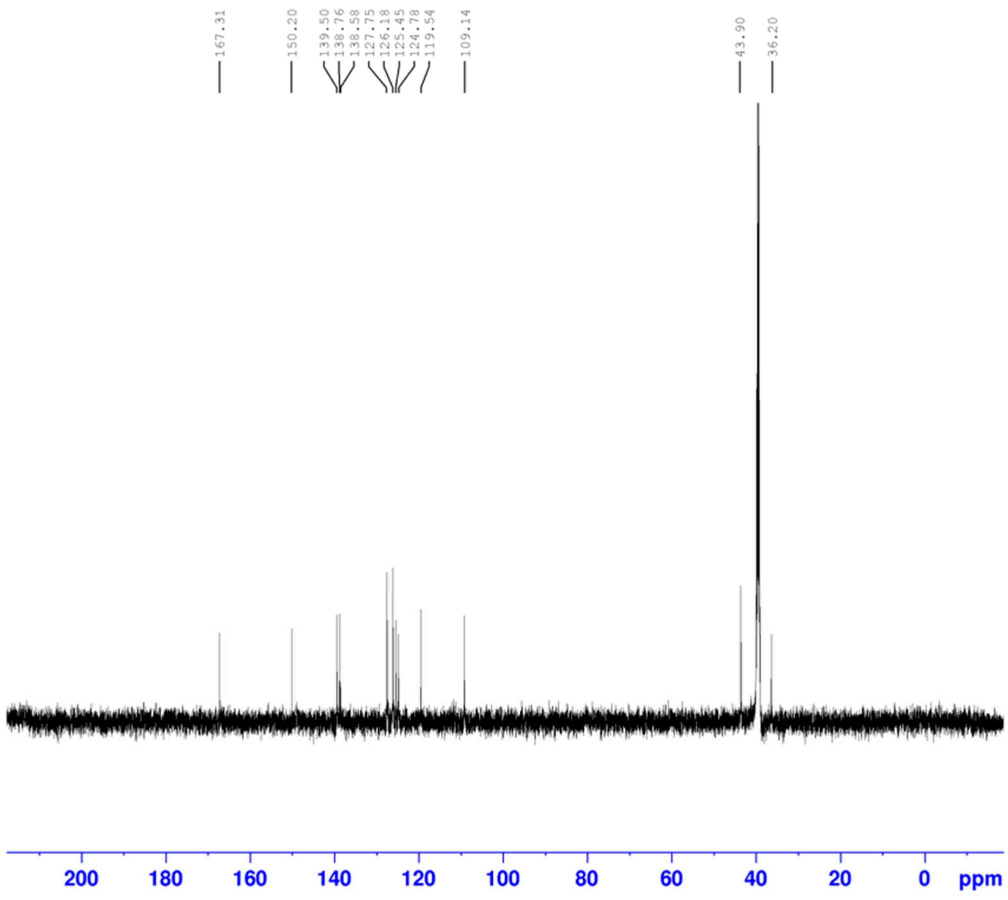

Figure S41. ^{13}C NMR Spectrum of compound **10**

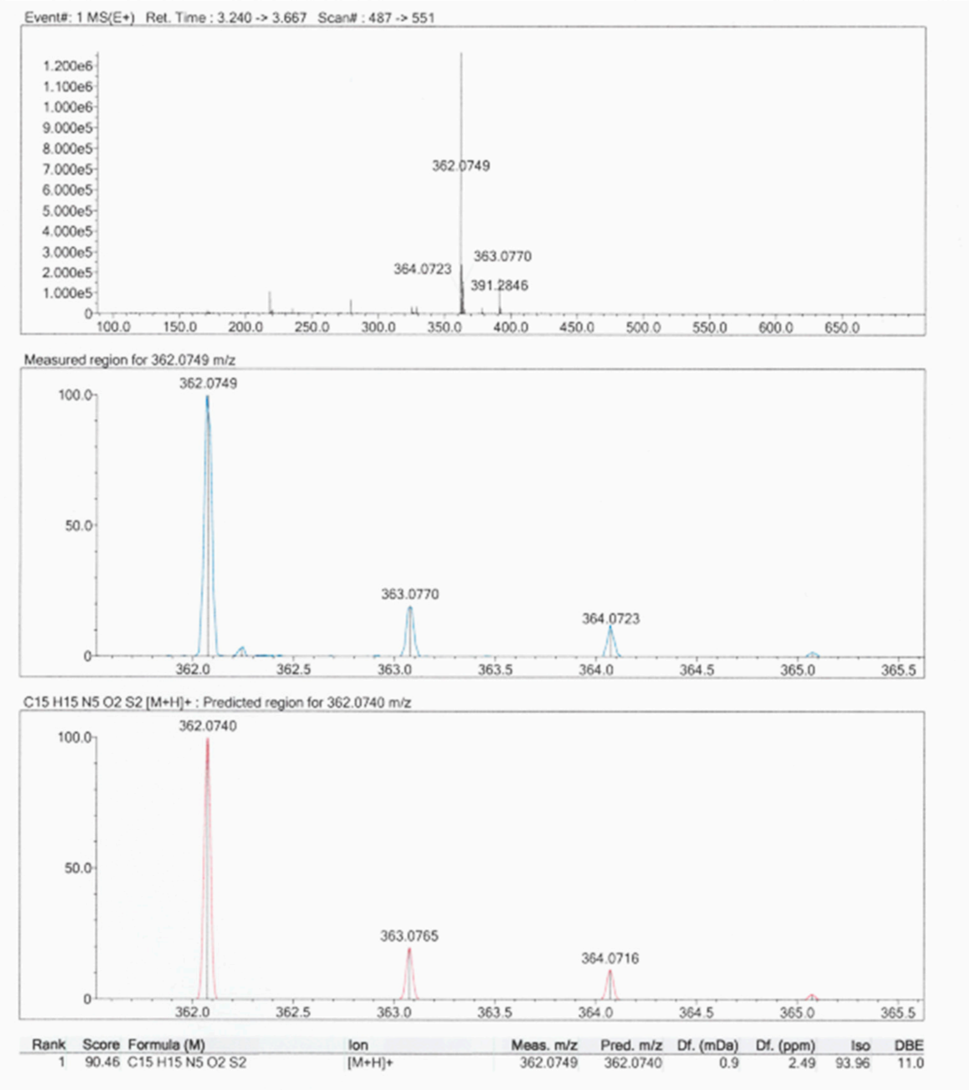

Figure S42. Mass Spectrum of compound **10**

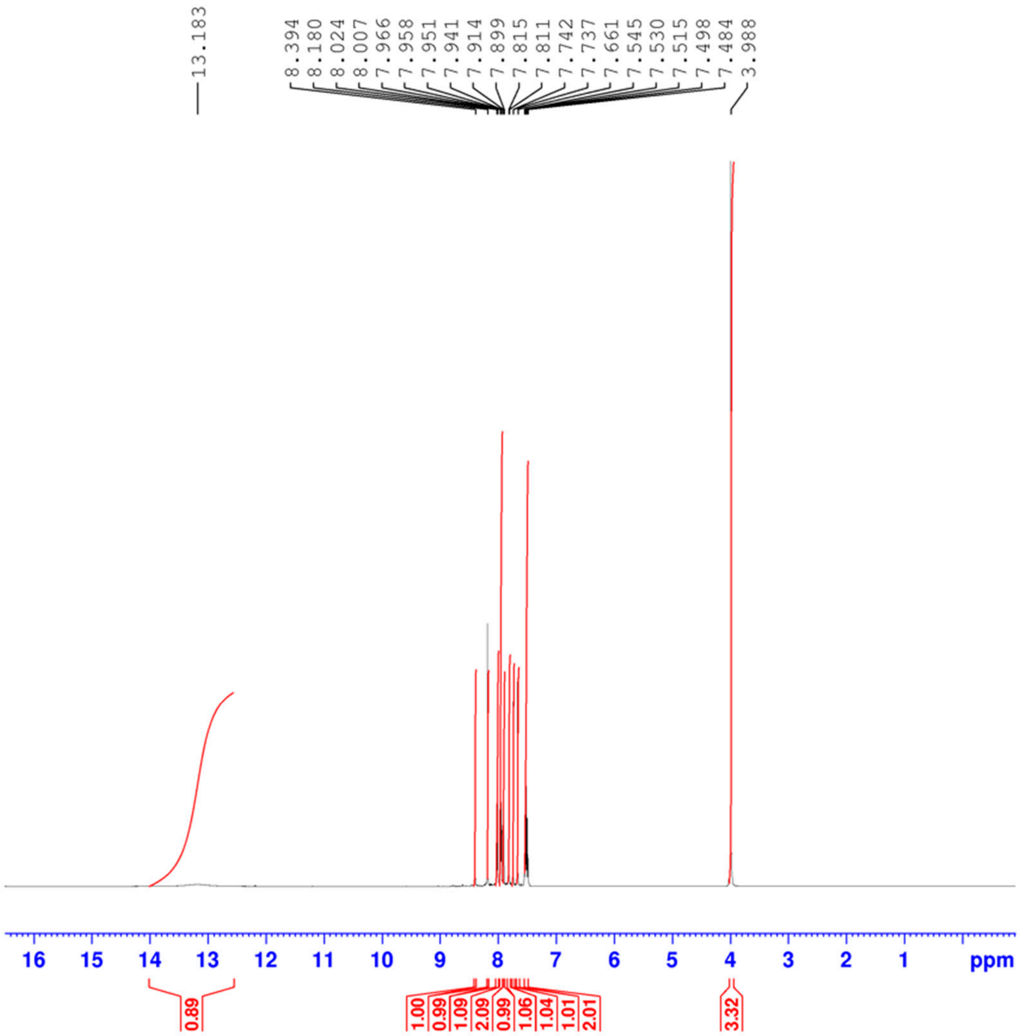

Figure S43. ^{1}H NMR Spectrum of compound **11** (0-15 ppm)

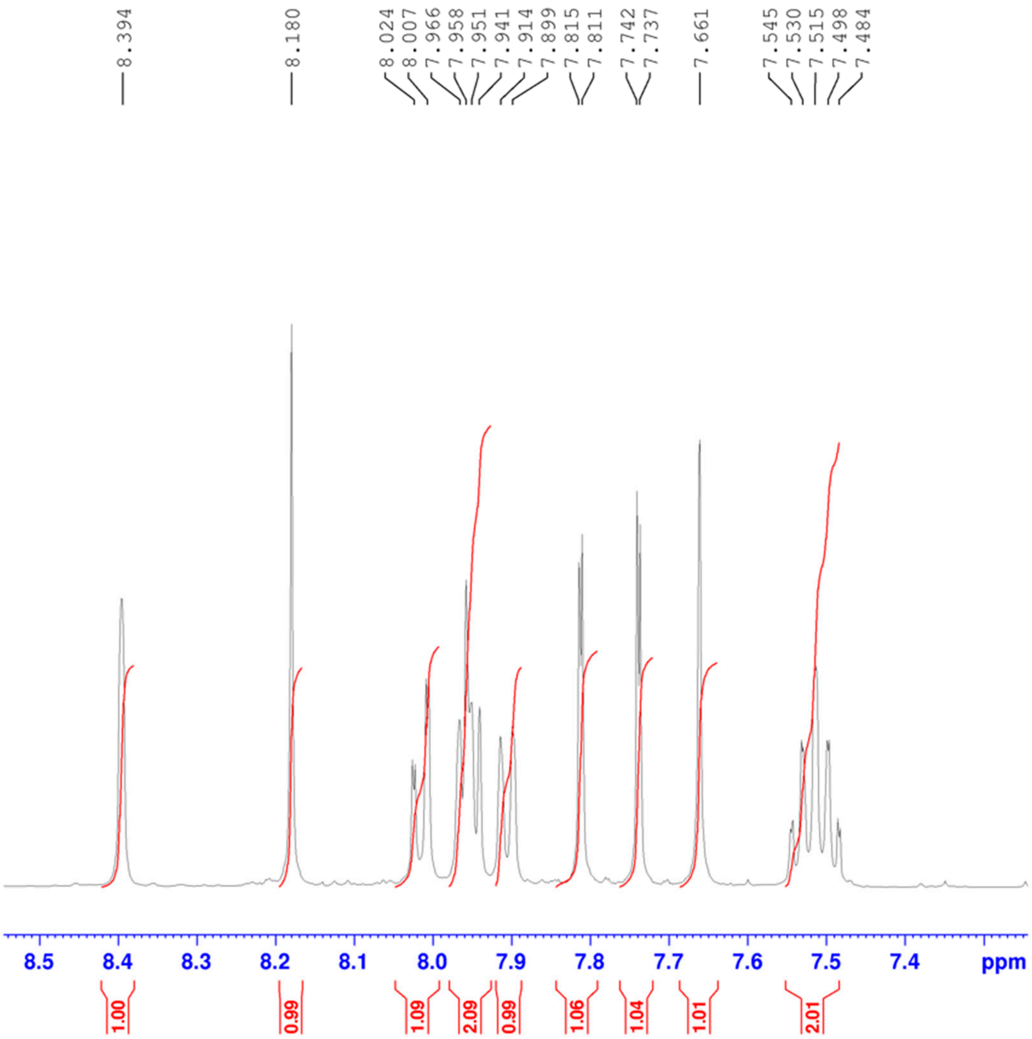

Figure S44. ^{1}H NMR Spectrum of compound **11** (7-9 ppm)

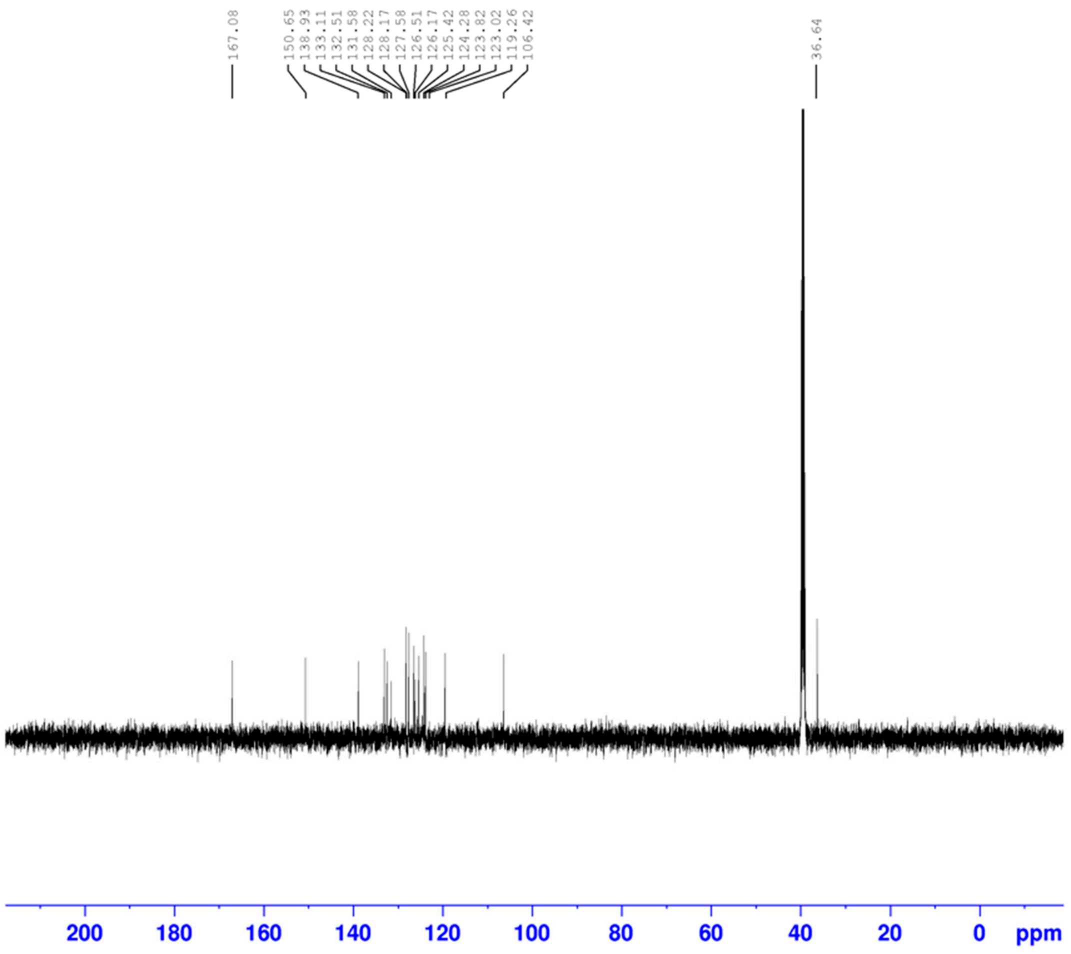

Figure S45. ^{13}C NMR Spectrum of compound **11**

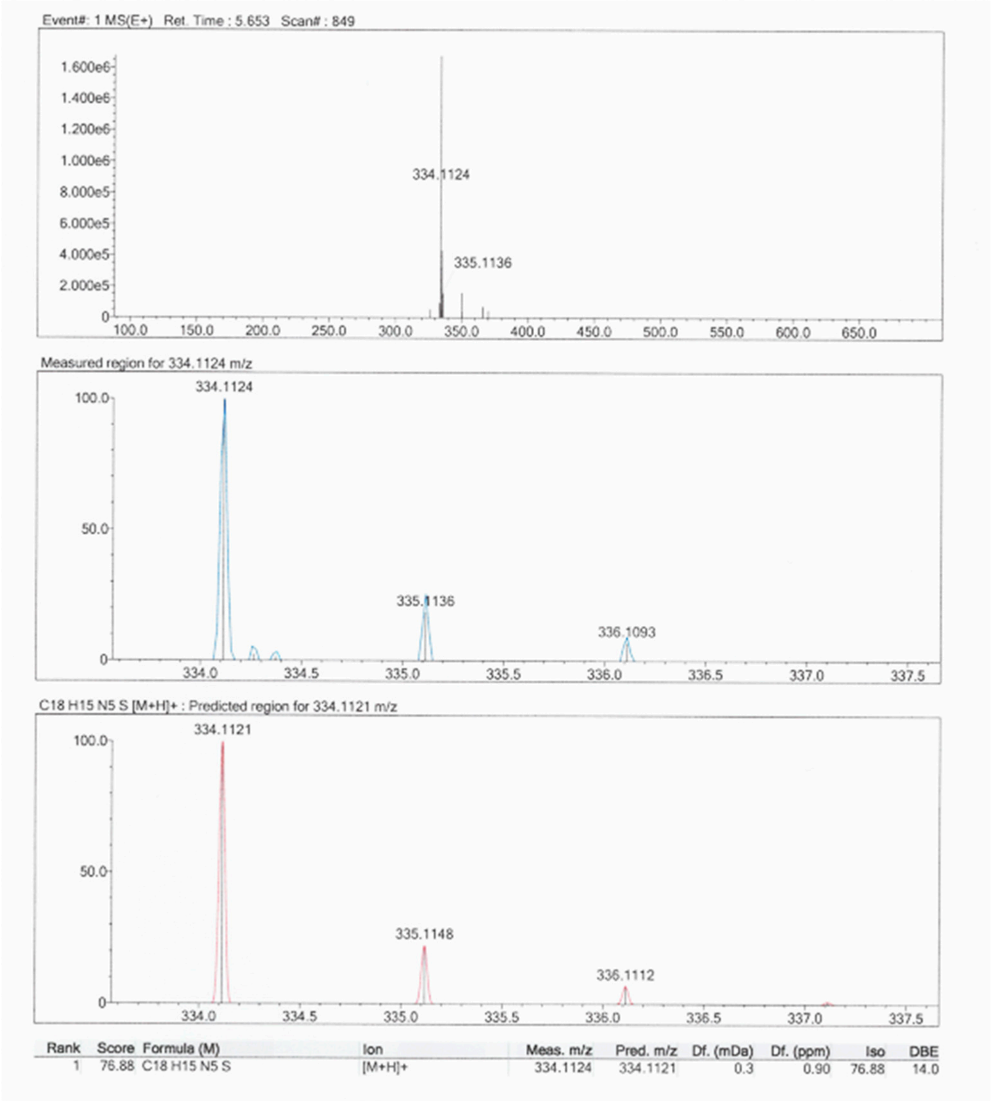

Figure S46. Mass Spectrum of compound **11**

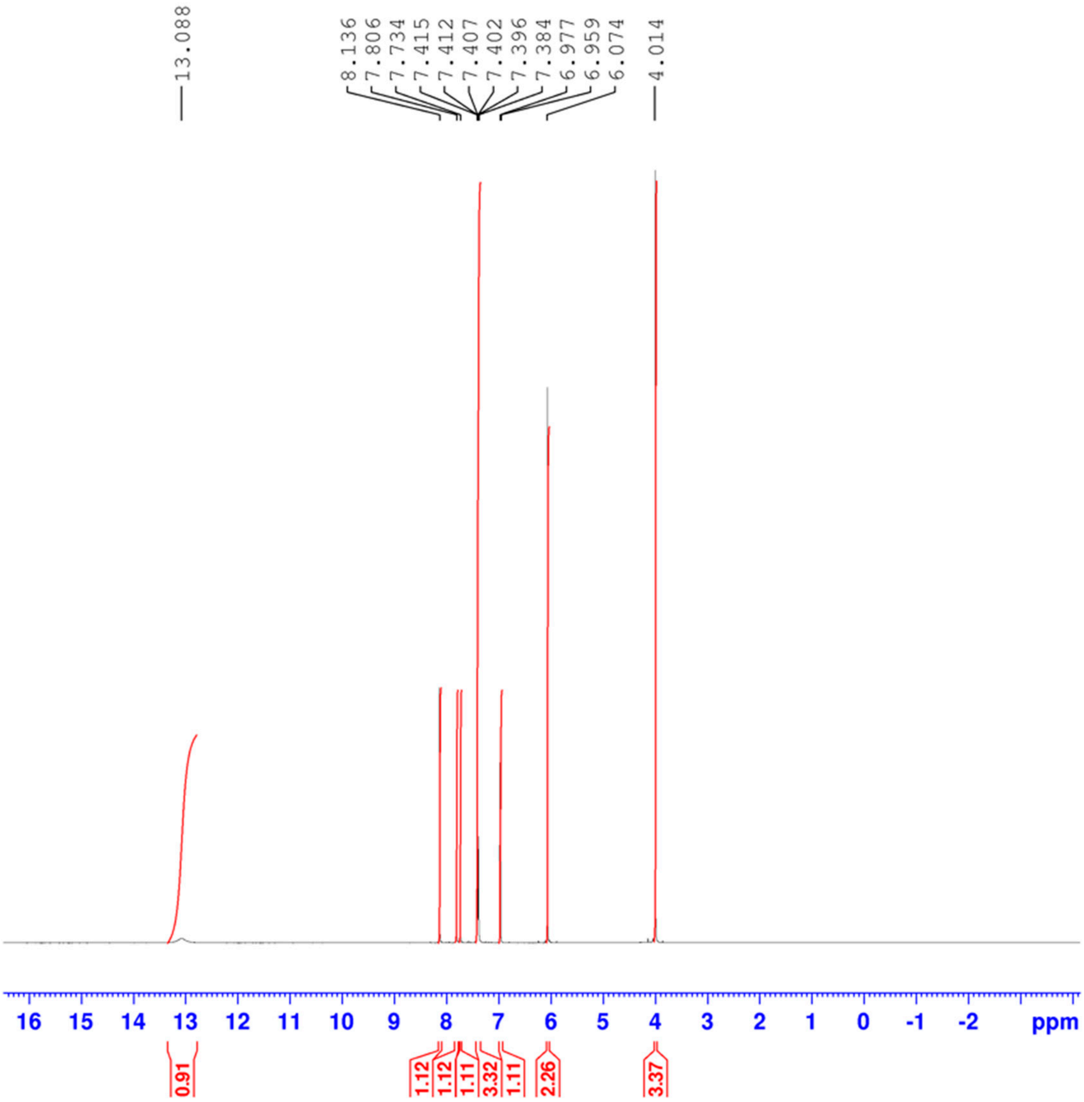

Figure S47. ^{1}H NMR Spectrum of compound **12** (0-15 ppm)

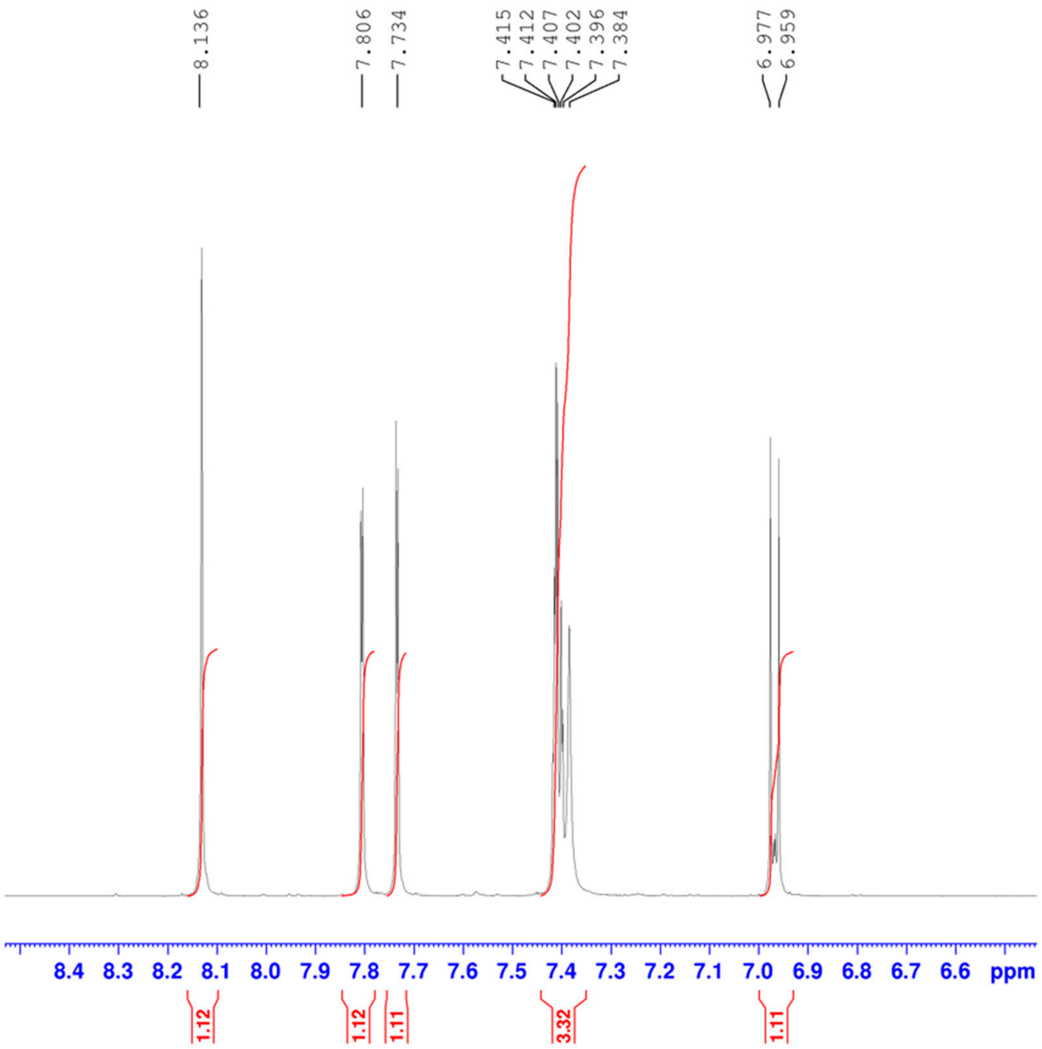

Figure S48. ^{1}H NMR Spectrum of compound **12** (7-9 ppm)

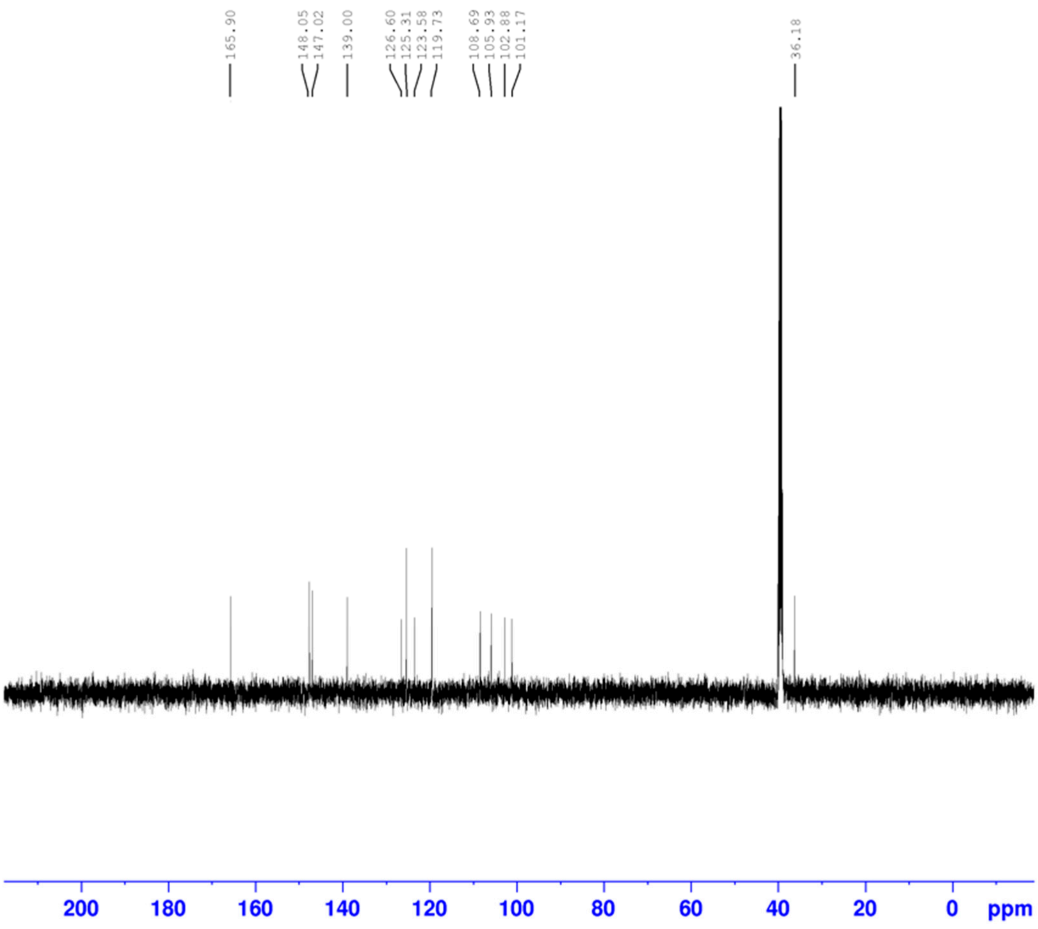

Figure S49. ^{13}C NMR Spectrum of compound **12**

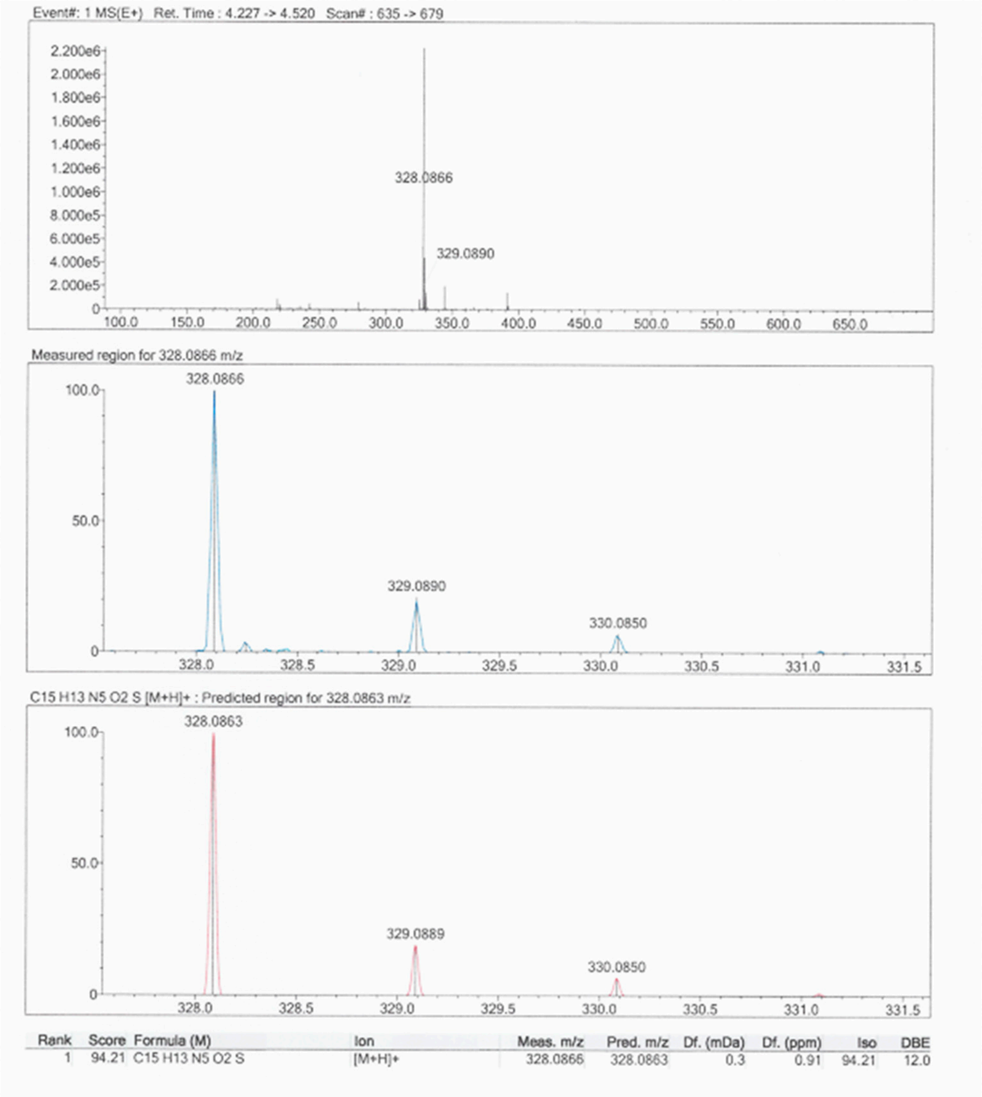

Figure S50. Mass Spectrum of compound **12**

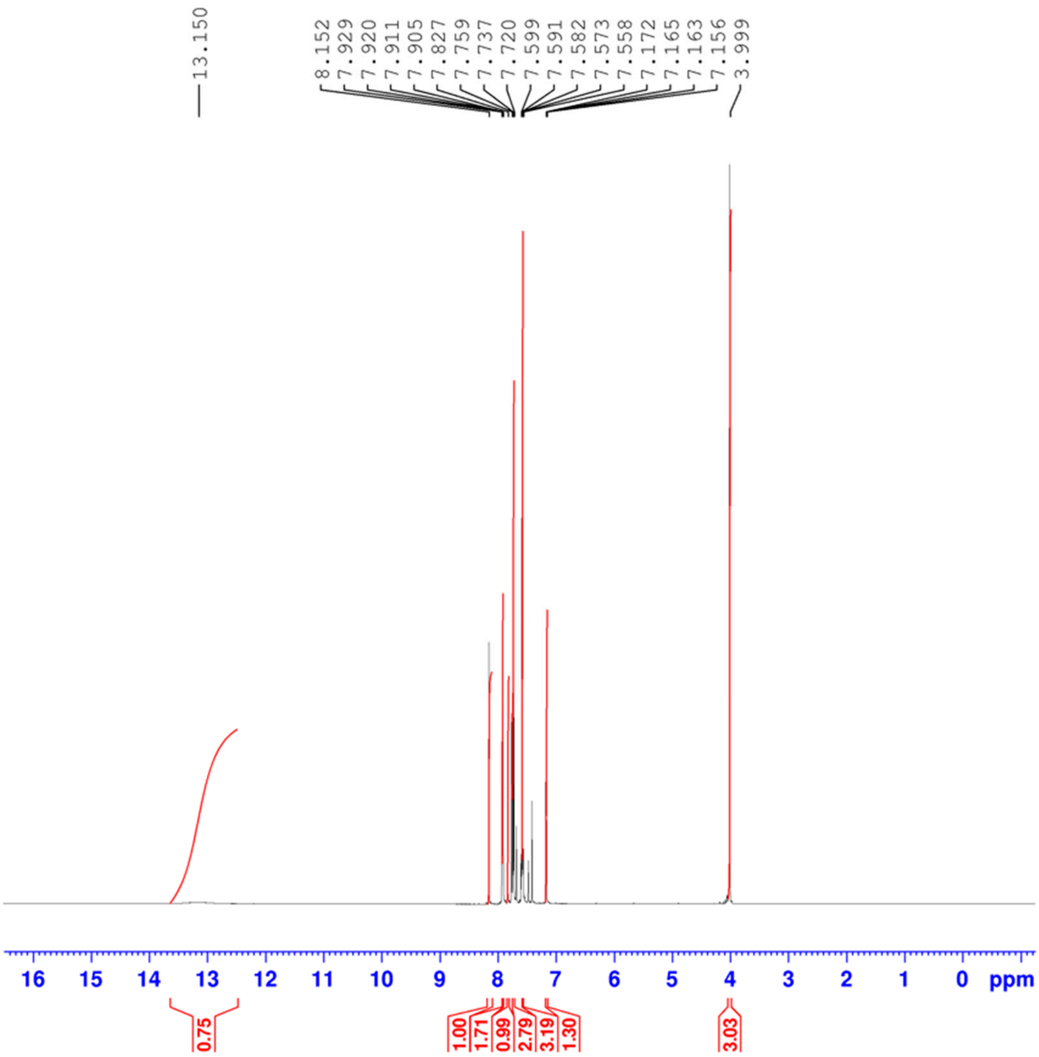

Figure S51. ^{1}H NMR Spectrum of compound **13** (0-15 ppm)

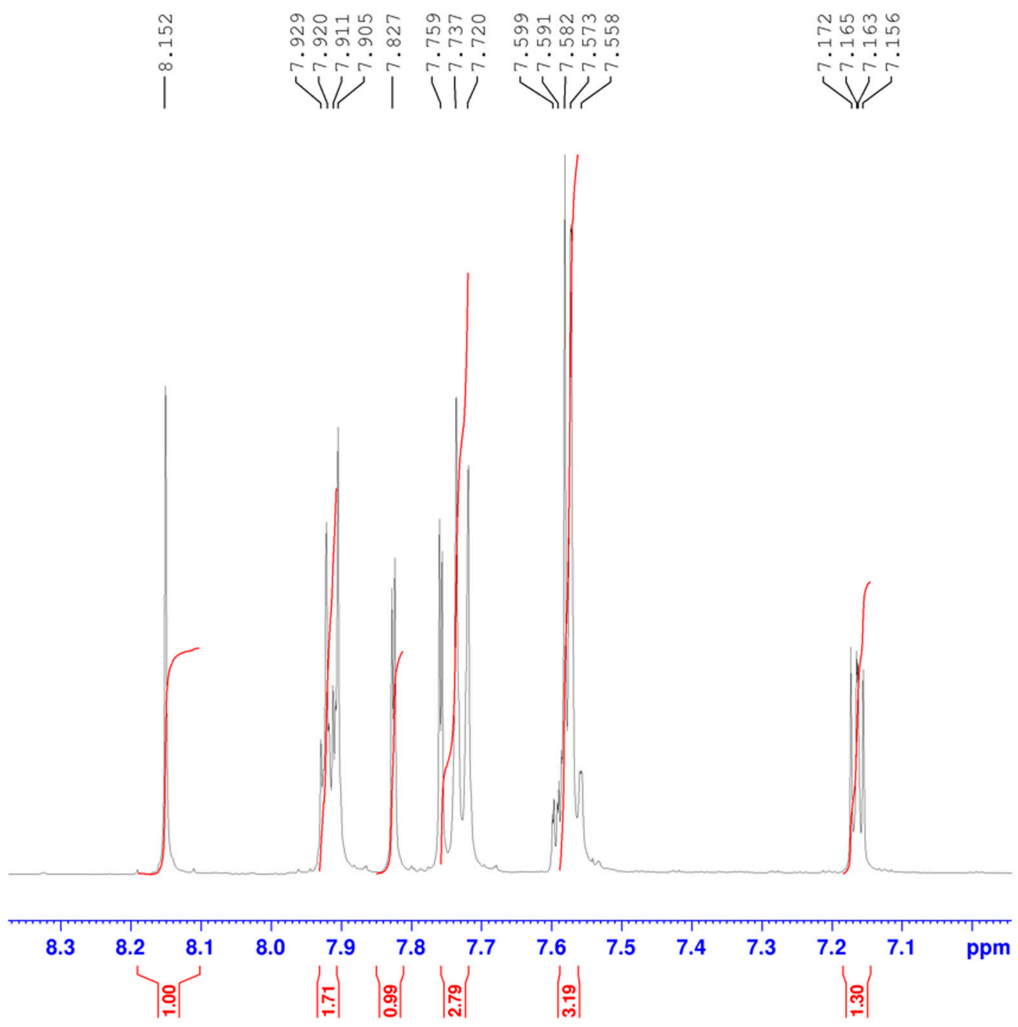

Figure S52. ^{1}H NMR Spectrum of compound **13** (7-9 ppm)

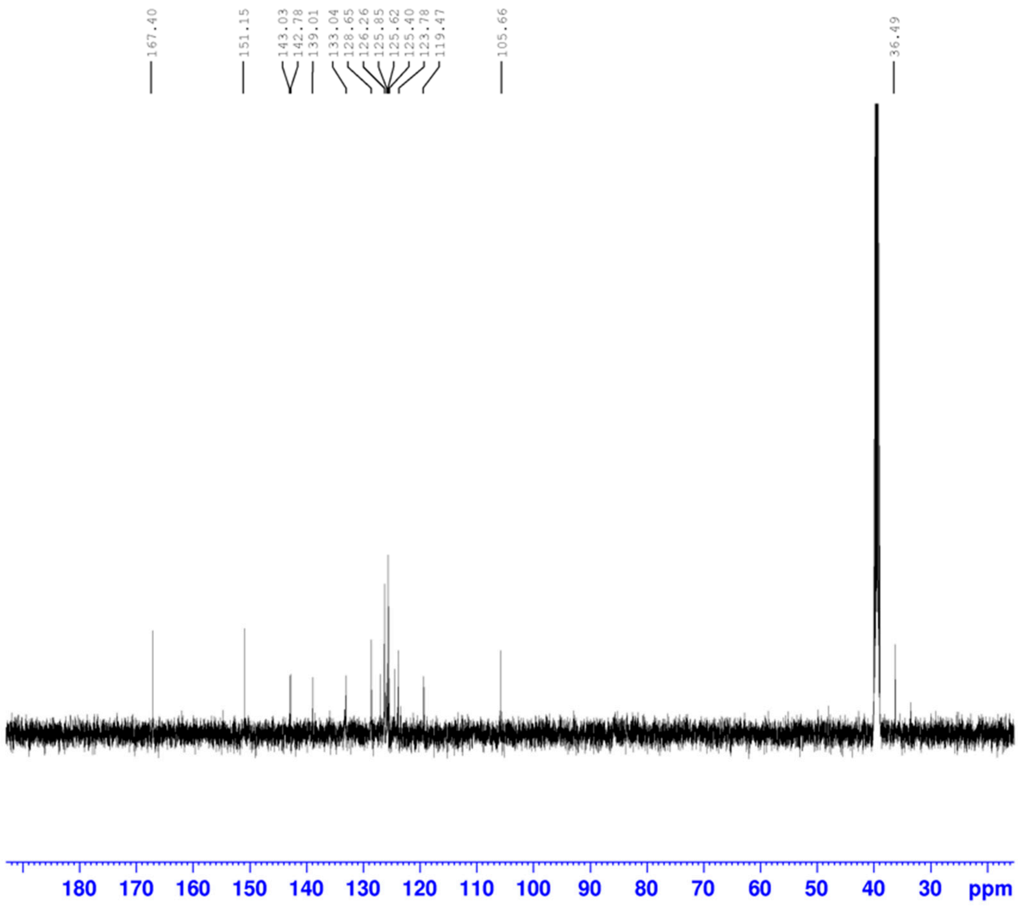

Figure S53. ^{13}C NMR Spectrum of compound **13**

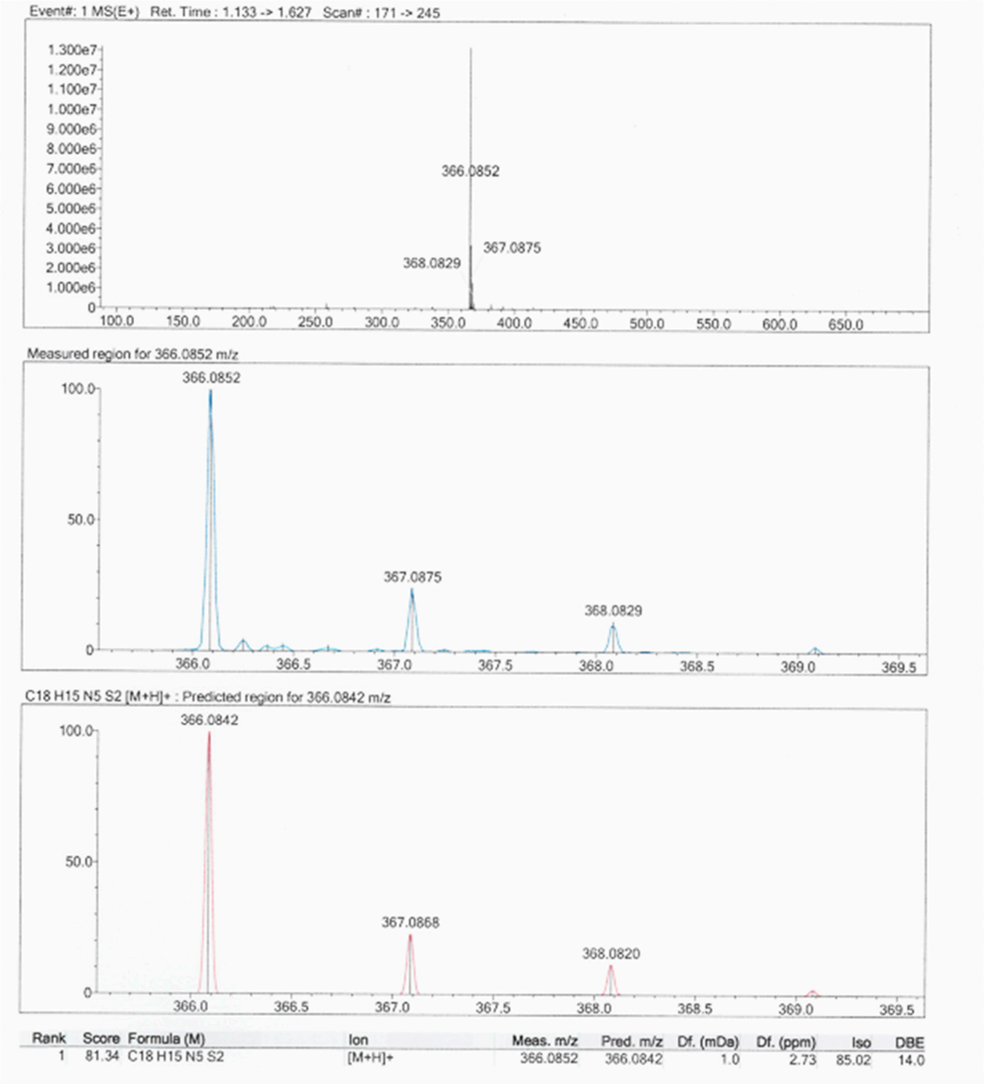

Figure S54. Mass Spectrum of compound **13**

Table S1. Prime MM-GBSA binding free energy (ΔG_{bind}) and energetic decomposition (kcal/mol) at the EGFR and AR binding sites

				EGFR				
**Compound**	**Coulomb**	**Covalent**	**Hbond**	**Lipo**	**Packing**	**$Solv_{GB}$**	**vdW**	**ΔG_{bind}**
**5**	-9.13	2.78	-1.13	-17.79	-0.88	31.25	-49.20	-44.10
**13**	-11.26	11.48	-0.15	-24.00	-0.65	34.89	-52.05	-41.75
**Tak-285**	-27.56	2.54	-2.32	-25.66	-0.26	39.36	-76.46	-90.38
				**AR**				
**Compound**	**Coulomb**	**Covalent**	**Hbond**	**Lipo**	**Packing**	**$Solv_{GB}$**	**vdW**	**ΔG_{bind}**
**5**	-11.52	10.30	-1.25	-28.61	-8.50	53.02	-49.57	-35.76
